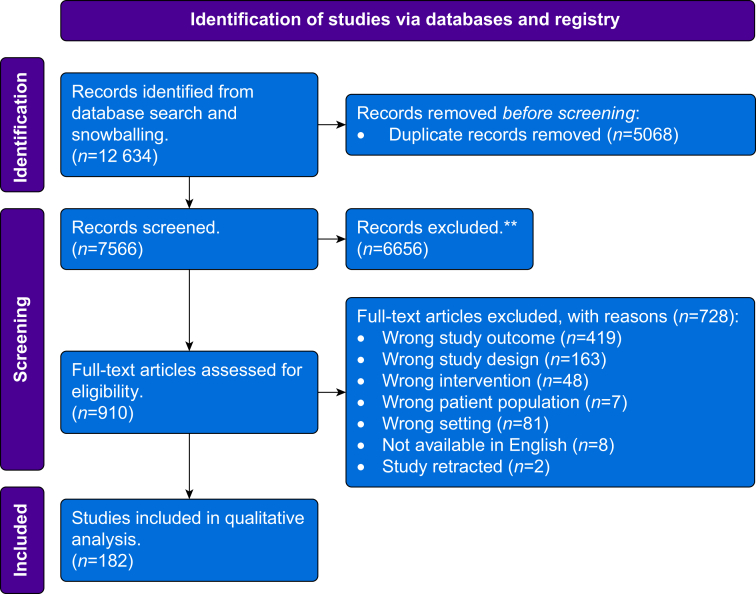# Artificial intelligence for electrocardiographic diagnosis of perioperative myocardial ischaemia: a scoping review

**DOI:** 10.1016/j.bja.2025.05.037

**Published:** 2025-07-04

**Authors:** Anne Kim, Mitchell Chatterjee, Alla Iansavitchene, Majid Komeili, Adrian D.C. Chan, Homer Yang, Jason Chui

**Affiliations:** 1 Schulich School of Medicine and Dentistry, University of Western Ontario, London, ON, Canada; 2 Department of Systems and Computer Engineering, Carleton University, Ottawa, ON, Canada; 3 London Health Science Centre, London, ON, Canada; 4 Department of Anesthesia & Perioperative Medicine, University of Western Ontario, London, ON, Canada

**Keywords:** artificial intelligence, ECG, electrocardiography, machine learning, monitoring, myocardial infarction, myocardial ischaemia, remote home monitoring

## Abstract

**Background:**

Perioperative electrocardiographic monitoring can offer immediate detection of myocardial ischaemia, yet its application in perioperative and remote monitoring settings is hampered by frequent false alarms and signal contamination. We performed a scoping review for the current state of artificial intelligence (AI) in perioperative ECG interpretation.

**Methods:**

A literature search in Ovid MEDLINE, EMBASE, Compendex, and CINAHL databases was performed from inception to May 10, 2023. All original research of ECG monitoring for myocardial ischaemia, myocardial infarction, or both was included.

**Results:**

A total of 182 original research articles published between 1991 and 2023 were included. Most studies (*n*=132) used a pre-existing ECG database to develop AI algorithms retrospectively, and the rest did not specify their sources. Processing filters were used in 58% of the studies to remove ECG noises/artifacts before AI algorithm development. Amongst the AI technologies used, ResNet demonstrated the highest median sensitivity, precision, and specificity at 98.4%, 99.8%, and 99.1%, respectively. There are only five studies with intermittent prospective ECG collection on ST-segment elevation myocardial infarction. No studies prospectively collected continuous ECG perioperatively, with frequent false alarms and signal contamination.

**Conclusions:**

AI technology can achieve high diagnostic accuracy for myocardial ischaemia detection in clean intermittent electrocardiograms. However, almost all these algorithms were developed from a few open-source clean ECG databases without testing on ‘noisy data’, which limited their clinical applicability in the perioperative setting where signal contamination is frequent. AI algorithms on perioperative electrocardiography, tested in a noisy perioperative and remote monitoring environment, including wearable devices, are needed.

**Editor’s key points**

• Artificial intelligence (AI) algorithms can detect myocardial infarction on clean ECG data with relatively high accuracy, but their performance in real-time perioperative and noisy ECG settings remains largely untested.
• This review highlights current AI applications available for ECG-based myocardial infarction detection and highlights critical gaps in perioperative use.
• Future efforts should validate AI models in real perioperative settings, which could allow integration into wearable devices for continuous monitoring.

Perioperative myocardial infarction can occur in one of 33 in-hospital patients after noncardiac surgery.^1^ Early studies of myocardial ischaemia, a preceding event of myocardial infarction (MI), noted an incidence of ischaemia in up to 41% of postoperative patients, linked to a 2.8-fold increase in all adverse cardiac outcomes and a 9.2-fold increase in the odds of any ischaemic event.^2^ The incidence of true perioperative MI in patients who underwent noncardiac surgery, according to recent studies, ranged from 3.5% to 19.1%.^3^^,^^4^ Perioperative MI is mainly caused by an imbalance of oxygen supply and demand, termed as Type 2 MI, which may be prevented with a timely restoration of the imbalance by treating with beta-blockers^5^, ^6^, ^7^, ^8^ or nitroglycerin.^5^^-^^9^ However, more than 60% of perioperative MIs are asymptomatic,^10^^-^^11^ making the timely diagnosis and intervention challenging.

Current monitoring for myocardial injury after noncardiac surgery^4^ involves daily assessment of troponin levels.^12^ Although troponins have prognostic value for perioperative and long-term outcomes, the intermittent nature of this monitoring strategy lacks an effective, timely mechanism to detect and interrupt the ischaemia cycle. In the ambulatory surgery setting, high-risk patients often face a dilemma: prolonged hospitalisation for monitoring or early discharge with potential missed complications. In contrast, continuous monitoring of ST-segment changes in ECG signals can provide immediate detection of myocardial ischaemia. Monitoring ST segments of five-lead ECG, leads II and V_5_, intraoperatively has been used to detect intraoperative myocardial ischaemia and Type 2 MI for decades.^13^ Anecdotal reports suggest that remote postoperative ECG monitoring may also allow for the timely detection of cardiac events to interrupt the ischaemia cycle and reduce postoperative MI.^14^, ^15^, ^16^ However, frequent false alarms from signal noise in remote settings can lead to alarm fatigue (desensitisation and ignoring the alarm),^17^^,^^18^ hence compromising patient safety.^17^^-^^19^

Recent studies show that artificial intelligence (AI) has improved ECG interpretations. We conducted this scoping review to map out the current state of knowledge on implementing AI technology for ECG interpretation of myocardial ischaemia and infarction in perioperative and remote settings.

## Methods

Before commencing this scoping review, we confirmed no similar articles in the JBI Database of Systematic Reviews and Implementation Reports, Cochrane Database of Systematic Reviews, or the International Prospective Register of Systematic Reviews (PROSPERO).

Our research question was: ‘What evidence supports the use of AI for ECG diagnosis of myocardial ischaemia and MI in the perioperative and remote monitoring settings?’ The population was ‘post-surgical patients, either in-hospital or discharged from the hospital’, the concept ‘AI’, and the context ‘ECG diagnosis of perioperative myocardial ischaemia or MI in noncardiac surgery patients’. Reporting followed the Preferred Reporting Items for Systems Reviews and Meta-Analyses Reporting Guideline Extension for Scoping Reviews (PRISMA-ScR) guidelines.^20^

### Search strategy

A comprehensive search strategy was developed in collaboration with a medical librarian. Upon the librarian's recommendation, the search scope included atrial fibrillation (AF) alongside MI, inclusive of any in-patient (e.g. medical patients) and out-patient (e.g. Holter) settings. This strategic decision was made to reduce the likelihood of missing relevant studies, as some studies may be indexed under AF owing to inconsistent database indexing, and to ensure comprehensive coverage of relevant literature.

Articles were systematically identified from the Ovid MEDLINE, EMBASE, Compendex, and CINAHL databases, along with snowballing of references from the included articles, conducted on May 10, 2023. There was no restriction on publication dates. We limited our search to ‘human study’ and ‘English language’. Key search terms included AI, ECG, MI, myocardial ischaemia or injury, AF, and STEMI. We also accounted for ‘older’ terminology such as bioinformatics, automated learning, computational intelligence, machine learning, and deep learning. Detailed strategies and results appear in Supplementary material 1. References were exported to COVIDENCE™ (Covidence, Melbourne, Australia) with duplications removed.

### Screening and selection

Two authors (AK and JC) independently screened titles and abstracts, followed by full-text reviews of selected articles. Disagreements were resolved through discussion; a third reviewer (HY) was available to resolve conflicts but was not needed. We included all original research articles that specifically assessed the ECG detection of myocardial ischaemia or MI using AI. Notably, we excluded data that exclusively focused on AF, as this was not our primary aim and based on our search, we had found numerous systematic reviews looking at AI in detecting AF.^21^, ^22^, ^23^ There were no restrictions on AI model type or ECG data source, including studies that explored wearable ECGs.

### Data extraction

The extracted information was categorised into three main areas: (1) general study details, including study design, publication year and country, cohort sizes, patient characteristics, ECG type, and databases used; (2) AI technology and reference standards used; and (3) the recall (or sensitivity), specificity, precision (or positive predictive value [PPV]), negative predictive value (NPV), and overall diagnostic test accuracy of the AI technology assessed.

The terminology used to describe diagnostic accuracy in engineering literature slightly differs from that used in clinical epidemiology literature. We listed the definitions of these metrics in Supplementary Table S1 for clarification.

### Data analysis

As this was a scoping review, the analysis was primarily narrative, addressing the following questions:

1. What AI algorithms have been developed for perioperative MI/ischaemia ECG diagnosis?
2. How accurate are AI-based algorithms compared with physician interpretation?
3. How do different AI models (e.g. machine learning, deep learning) or specific patient subsets affect diagnostic accuracy?
4. What are the key limitations and knowledge gaps in perioperative and remote monitoring AI applications?

Because of the heterogeneity of the study’s aims and limited reporting qualities across studies (e.g. sample sizes were missing in most studies), quantitative meta-analysis was not feasible. Instead, we adopted a descriptive analysis approach, primarily summarising available data with median (interquartile range [IQR]) and counts (frequency), while acknowledging its inherent limitations.

## Results

Our initial systematic search yielded 12 634 records. After removal of 5068 duplications, we first screened the titles and abstracts of 7566 records and followed with a full-text review of 910 articles. We extracted data from 182 studies to construct our results (see Supplementary material 2 for full citations). We excluded reports lacking clear, quantifiable components or deviating from our predefined outcomes and methods (see Appendix 1). Of note, we identified 355 studies on AI-assisted AF interpretation that were excluded as they were out of scope for this review.

### Study characteristics

The 182 studies included in this review span from 1991 to 2023, in which the number of studies increased exponentially in the past 5 yr (Supplementary Fig. S1). These studies originated from various countries, and notably, the leading countries were China and India (*n*=47 and *n*=28, respectively).

### ECG data source

The majority of these studies are of retrospective design (*n*=177) and used existing open-source ECG databases to construct their AI algorithms; specifically, the PTB/PTB-XL database^24^ was used in 91 studies, the European Society of Cardiology ST-T database^25^ was used in 22 studies, and MIT Physionet/MIT-BIH databases were used in nine studies.^26^ A summary of the characteristics of the commonly used open-source ECG databases is listed in Supplementary Table S2. A substantial number of studies (*n*=54) did not specify the sources of their ECGs. In all included studies, both the training and validation datasets were derived from the same ECG database; none used an external validation dataset. Case reports and series were excluded from the analysis, except for those involving wearables, which were included in the narrative review to explore their application in remote ECG monitoring. The study characteristics of all included studies are listed in Supplementary Table S3.

### Reference standards

Among the 177 included studies, the most used reference standard was ECG interpretations manually annotated by cardiologists (*n*=171). Two studies relied on ECG interpretation by attending physicians, whereas four studies used coronary angiography as the confirmatory diagnostic method. The remaining studies did not specify the reference standard.

### Types of artificial intelligence techniques used

AI techniques investigated in the primary studies were broadly categorised into algorithm based (*n*=51), traditional machine learning (*n*=27), signal processing (*n*=2), neural network (*n*=90), and hybrid network (*n*=12) (Table 1 and Supplementary Table S4). Historically, AI algorithms for detecting MI or myocardial ischaemia have evolved from expert systems to classical machine learning, and more recently, to deep learning, reflecting the increasing sophistication of AI algorithms.

**Table 1:** Summary of AI technology. AI, artificial intelligence; STEMI, ST-segment elevation myocardial infarction.

Type of AI	Description	Detection of ECG changes	Advantages	Limitations
Rule-based algorithms	Operate on predefined rules or conditions set by the study	Rely on predefined fixed thresholds in ECG parameters to determine what is called an ST-segment change (elevation, depression, duration, or amplitude) to determine as MI	• Highly interpretable; logic is transparent and easy to audit • Customisable for specific study points: sensitivity to ST depressions *vs* STEMI	• Lack flexibility • Performance not consistent when applied to real-world ECGs with atypical/noisy features • Do not learn from data; cannot improve with input over time
Traditional machine learning (i.e. support vector machine, decision trees)	Trained with relevant features and clinical data to build predictive models by using specifically extracted ECG features (ST slopes, QRS duration)	Use manually engineered features from clinical ECG data (with ECG changes or control) to identify ischaemic or MI changes and classify as normal *vs* ischaemic	• Better generalisation ability • Simple to implement and validate	• Unable to capture more complex signals • Require expertise for initial feature extraction (subject to human bias) • Unable to perform well in raw high-noise environments
Artificial neural networks	Learns intricate patterns and non-linear relationships from data, and interconnects these layers via weighted sums and non-linear transformations	Can adapt to variations and noise to capture more complex ECG signals. Can detect ischaemic patterns from non-linear relationships across features	• Feedforward neural network, complex information features can be extracted and predicted • Flexible: can handle structured and unstructured data	• Not architecturally optimised for spatial or temporal data, such as ECG • Requires more data to generate network
Convolutional neural networks	Designed for grid-like data; incorporates convolutional layers adept at discerning spatial patterns, can apply specialised filters or kernels to each layer	Filters applied to extract specific ECG spatial patterns, i.e. ST-segment elevation/depression; more targeted for grid-like data (ECG)	• Highly effective in ECG signal processing: for classification • Fast training process • Automatically learns features without manual input	• Limited in depth: confined to tens–hundreds of convolutional layers, cannot incorporate more • Cannot capture long range temporal patterns • Performance degrades in highly noisy environments
Residual Networks (ResNet)	Encompass hundreds of layers, can incorporate skip connections (residual blocks) to circumvent/bypass intermediate layers or amalgamate layers	More complex ECG signals captured owing to its ability to encompass more specialised layers, such as deep hierarchical representations. Can identify subtle changes in ECG spanning longer sequences (i.e. ischaemia)	• Can support networks with hundreds of layers without training degradation • High accuracy in detecting complex ECG patterns • Better generalisation to real-world variability in ECG	• Not as well studied for use of ECG changes, especially in real-time perioperative contexts, despite being most complex/promising • Computationally expensive

Expert systems (AI algorithms) in ECG interpretation typically rely on predefined rules or established thresholds to identify ischaemic changes, such as ST-segment deviations.^27^ For instance, a rule-based system might detect an ST-segment elevation or depression exceeding a set amplitude or duration as myocardial ischaemia. Such expert systems, although effective in controlled settings, may struggle with complex or noisy ECG signals that fall outside predefined parameters.

Traditional machine learning approaches, such as support vector machines (SVMs) and decision trees, involve manual feature engineering, where relevant ECG characteristics (such as ST segment and T wave) are manually selected to train and build predictive models for identifying ischaemic events in new ECG data.^28^ Although effective for simpler tasks, these methods may not fully capture complex, non-linear relationships inherent in ECG data.

Deep neural networks (DNNs) address this by learning intricate patterns and non-linear relationships directly from raw ECG signals without manual feature extraction.^29^ Amongst these, convolutional neural networks (CNNs) use convolutional layers adept at discerning spatial patterns within input ECG data to apply specialised filters or kernels to ECG signals and capture nuanced features such as ST segments.^28^ ^29^ By interconnecting all detection-specific layers, CNNs can learn and interpret higher-level representations and make more accurate predictions than more general artificial neural networks (ANNs), as CNNs are targeted to interpret grid-like data, such as ECGs.^29^

The use of Residual Networks^30^ and Densely Connected Convolutional Networks (DenseNet) marks a significant advancement in CNN architectures, capable of delving deeper into data complexities. ResNet, with its capacity to encompass hundreds of layers compared with CNN's tens to hundreds, uses residual blocks to incorporate shortcut connections, known as skip connections, circumventing one or more layers and seamlessly amalgamating all layers’ learnings into an outcome^30^. DenseNet, by contrast, relies on dense connections between layers, facilitating efficient gradient flow throughout the network and mitigating challenges such as vanishing gradients or data overload often encountered in traditional CNNs.

Hybrid networks such as the CNN-LSTM hybrid network harness Long Short-Term Memory (LSTM) networks’ capabilities to address the vanishing gradient problem and capture temporal dynamics within data. This hybrid approach empowers CNNs to extract intricate features from ECG signals while LSTM networks adeptly detect temporal patterns over time, augmenting the algorithm's efficacy in MI detection scenarios.

A more detailed description of the AI technologies is provided in Supplementary material 3.

### Overall diagnostic performance of artificial intelligence techniques

AI algorithms exhibit strong overall diagnostic performance, with median sensitivity and specificity of 89.0% (82.7–95.9%) and 93.3% (84.9–97.7%), respectively. The PPV and NPV were 85.0% (63.9–93.4%) and 96.3% (91.8–99.2%), and the accuracy and area-under-curve (AUC) were 91.6% (82.5–96.5%) and 91.9% (89.2–97.0%) (Table 2 and Supplementary Table S5).

**Table 2:** Summary of diagnostic values of AI algorithms in different settings. Data are presented as median (interquartile range). Hybrid network refers to a combination of convolutional neural network (CNN), CNN-Long Short-Term Memory (LSTM), and Residual Network (ResNet) methods. AI, artificial intelligence; AUC, area-under-curve; PPV, positive predictive value; NPV, negative predictive value.

	Recall (sensitivity)	Specificity	Precision (PPV)	NPV	Accuracy	AUC
Overall (*n*=182)	94 (87.9–98.5)	95.2 (88.2–98.9)	92.1 (75.4–98.4)	97.6 (92.6–99.5)	96.4 (89.5–99.0)	96.6 (92.4–99)
Specific AI technique						
Algorithm based (*n*=51)	89.0 (82.7–95)	93.3 (84.9–97.7)	85 (63.1–93.4)	96.3 (91.8–99.2)	91.6 (82.5–96.5)	97.4 (89.2–95)
Traditional machine learning (*n*=27)	94.1 (91.8–98.9)	96.7 (90.7–99.5)	90.3 (70–96.11)	95 (93.1–97.5)	98.2 (91.2–99)	99.8 (98.7–99.9)
Signal processing (*n*=2)	78.9 (73.6–84.3)	-	79.6 (77.3–82)	-	-	-
Neural network (*n*=90)	95.4 (89.8–98.5)	96.2 (88.8–99.1)	95.8 (88.6–99.3)	97.1 (95.4–99.4)	96.2 (91.5–99)	96.7 (94.9–98.9)
Hybrid network (*n*=12)	97.1 (91.7–98.8)	93.3 (90–98.5)	97.2 (76.8–99.3)	99.4	97.7 (95.4–99.3)	97.4 (94.5–98.3)
Type of ECG						
12-lead (*n*=149)	94.2 (88–98.2)	95.1 (88.1–99)	91.3 (76.9–98.4)	96 (91.3–99)	95 (89.1–98.8)	97.1 (92.6–99.1)
Two-lead (*n*=13)	91.7 (83.5–95.6)	97.5 (93.1–98.6)	87.5 (80.3–94.7)	99.78	99 (98.9–99)	94 (91–97)
One-lead (*n*=11)	99.5 (95.5–99.9)	98.2 (94.3–99.6)	99.7 (99.5–99.9)	-	99.6 (99.1–99.9)	96.7
ECG sources						
In-patient (*n*=174)	94.1 (88.6–98.5)	97.8 (88.3–99.1)	90.3 (74.9–98.4)	97.1 (94.3–99.5)	95.8 (89.9–99)	96.7 (92–98.9)
Out-patient (*n*=5)	94.9 (88.4–99.7)	98.6 (92.2–99.9)	-	-	99.4 (98.9–99.9)	-
Smart watch (*n*=3)	93 (85.4–100.0)	95 (82.4–100.0)	-	-	-	-
Pre-processed ECG						
No pre-processed ECG (*n*=76)	91.9 (87.5–98.2)	94.6 (89.9–99.1)	89.5 (72.8–98.5)	98.3 (94.6–99.6)	96.5 (90.6–99)	99.3 (97.5–99.7)
Pre-processed ECG (*n*=106)	93.9 (89–98.6)	95 (86.3–98.6)	93 (76.9–98.1)	96 (90–99.3)	96.1 (89.5–98.9)	95.5 (91.9–97.9)

Neural networks were the most used AI technique (*n*=95), achieving consistently strong performance, with a median (IQR) sensitivity, precision, and specificity of 95.4% (89.8–98.5%), 95.8% (88.6–99.3%), and 96.2% (88.8–99.1%), respectively. Traditional machine learning-based techniques, although still effective, appear to have slightly lower precision with median (IQR) sensitivity, precision, and specificity of 94.1% (91.8–98.9%), 90.3% (70–96.1%), and 96.7% (90.7–99.5%), respectively (Table 2). Fewer studies examined hybrid and signal processing-based methods, reflecting their emerging status. Detailed diagnostic values are provided in Table 2.

### Diagnostic performances within neural networks

Within the neural network family, performance improved progressively from ANN, CNN, to the more advanced ResNet, highlighted in Table 2. The median (IQR) recall (sensitivity), precision, and specificity of the ANN technique, including ANN Bayesian, ANN Hermite representation, and ANN Glasgow techniques, are 88.6% (83.9–96.7%), 87.9% (77.7–93.0%), and 89.2% (78.4–96.2%). The CNN technique showed improved performance with a recall (sensitivity), precision, and specificity of 94.2% (87–97.1%), 89.8% (82.2–96.6%), and 93.2% (90–97.7%), respectively. The most advanced, ResNet, demonstrated the highest diagnostic accuracy with recall (sensitivity), precision, and specificity of 98.4% (96.2–99.0%), 99.8% (99.1–99.8%), and 99.1% (97.6–99.5%), respectively.

### Diagnostic performance between different ECG leads

Most studies used intermittent ECG strips composed of 12-lead ECG records (*n*=156), followed by two-lead ECG records (*n*=13) and one-lead ECG records (*n*=11). Other formats (three-, six-, eight-, nine-, and 15-leads) were used in nine studies combined. The median (IQR) recall (sensitivity), precision, and specificity for 12-lead ECG were 94.2% (88.0–98.2%), 91.3% (76.9–98.4%), and 95.1% (88.1–99.0%), respectively. The diagnostic performances in two-lead ECG and one-lead ECG are comparable and are listed in Table 2.

### Diagnostic performances between different settings

Most studies were retrospective studies using in-patient data. Only six studies prospectively collected ECG data; however, they had small samples (median=204 patients), with the smallest being a case report of one patient.^31^ ECG data were collected via smart watch in three studies: one case report of ‘inconclusive’ single-lead ECG despite 3 days of chest pain, and two studies with patients repositioning the device to simulate 12-lead ECGs. The patients in these studies were symptomatic or suspected of acute coronary syndromes, in or en-route to the emergency department or cardiac critical care unit, with mostly intermittent ECG strips, and none were collected as continuous perioperative ECGs.

When evaluating different sources of ECG data collected using in-patient, out-patient, and smart watch data, the comparison resulted in median (IQR) recall (sensitivity) values of 94.1% (88.6–98.5%), 94.9% (88.4–99.7%), and 93.0% (85.4–100.0%), and specificity values of 95.8% (88.3–99.1%), 98.6% (92.2–99.9%), and 95% (82.4–100.0%), respectively.

### Diagnostic performances without signal processing techniques

More than half of the studies (*n*=109) used pre-filtered ECG segments (i.e. only clean ECG segments were used to construct the AI algorithms). Notably, many AI algorithms were applied to the data after pre-processing filtering. If studies that used pre-processed data are excluded, the median (IQR) recall (sensitivity), precision, and specificity of AI algorithms are 91.9% (87.5–98.2%), 89.5% (72.8–98.5%), and 94.6% (89.9–99.1%), respectively. Pre-processed data have median (IQR) recall (sensitivity), precision, and specificity of 93.9% (89.0–98.6%), 93.0% (76.9–98.1%), and 95.0% (86.3–98.6%), respectively, suggesting comparable diagnostic performance between studies with and without a pre-processing filter.

## Discussion

This scoping review outlines the current landscape of AI algorithms for diagnosing MI and myocardial ischaemia. Whereas there has been robust exploration in developing AI for MI detection, real-time perioperative applications, particularly for ST depressions/non-ST-segment elevation myocardial infarction (NSTEMI), remain limited. Research largely follows two paths: (1) developing AI algorithms to improve diagnostic accuracy, albeit without taking the crucial next step of integrating these AI algorithms into perioperative practice, and (2) exploring wearables with embedded AI algorithms for ST-segment elevation myocardial infarction (STEMI) detection in ambulatory settings.

Most studies focus on engineering AI algorithms based on open-source ECG databases containing both normal and abnormal ECGs (e.g. ST elevation). Most of these studies applied a very similar methodology: databases are split into model derivation, validation, and testing datasets, followed by developing a new AI algorithm. The performance of these algorithms (test) is then compared against physician interpretation of ECGs, typically by two cardiologists, serving as the reference standard. Few studies used the diagnosis of coronary angiogram as the reference standard (Supplementary Table S4). Only five studies were prospective, but with methodological concerns: poorly defined inclusion criteria, small sample sizes (i.e. one study included only one patient, another with two patients), and underrepresentation of women (13%). The selection of the validation cohort in these prospective studies was also unclear. For instance, the validation cohort in the study of Chen colleagues^32^ included only 10 patients without clear inclusion or exclusion criteria. The target condition in all these prospective studies was STEMI, with none addressing ST depression/NSTEMI. Importantly, no algorithms were tested intraoperatively or after surgery, either during the hospital stay or after discharge as part of a remote home monitoring programme.

Among ambulatory wearables, the smart watch emerged as a popular choice recently in several studies (*n*=3). However, a significant limitation hindering its perioperative use is its ability to detect only a single-lead ECG.^33^ To overcome this limitation, study designs aimed to enhance lead detection by adjusting the smart watch's positioning using the Einthoven triangle as a guide. For instance, the study of Avila^34^ focused on obtaining Lead 1 as intended, whereas Leads 2 and 3 were achieved by having the patient hold the left or right thumb on the crown of the watch and placing the watch's back against the mid-abdomen. One study used an Apple Watch to detect pre-diagnosed STEMI and NSTEMI in 54 and 27 patients; sensitivity and specificity for STEMI were reported as 93% and 95%, and for NSTEMI, as 94% and 92%.^31^ The other reports primarily served as a demonstration of feasibility with small subject numbers (Avila^34^: *n*=2, Stark and colleagues^35^: *n*=1). These studies aimed to demonstrate the accuracy of STEMI detection on the Apple Watch against expert cardiologist readings on traditional ECGs. Challenges such as variability in wearable positioning for reliable lead detection highlight the need for further advancements in wearables and algorithm development in perioperative settings.

### Interpretation

Although recent AI algorithms show strong diagnostic performance, caution is needed when interpreting these high values, especially when they are applied in perioperative settings.

Real-time ECG data often contain noise and artifacts that can challenge the accuracy of AI algorithms. Many studies used pre-processing filters to try to eliminate these, but diagnostic performances remained relatively unchanged between pre-processed and non-pre-processed data. This observed phenomenon could be attributed to several factors. Firstly, many pre-existing databases are often meticulously curated, providing clean and well-annotated data. Consequently, the addition of pre-processing filters may not yield substantial changes in performance metrics as the data are already of high quality. Filtering may also have been unhelpful because the hardware instrumentation used to collect data often contain inherent hardware filters, making the additional digital filtering redundant. Furthermore, some studies deliberately chose not to incorporate extensive pre-processing filters. This decision might stem from the inherent robustness of their algorithms to noise and variations in input data. By focusing on algorithmic designs that inherently handle such challenges, these studies may have achieved consistent performance metrics without relying heavily on pre-processing techniques. In essence, the combination of high-quality training data from pre-existing databases and algorithmic robustness to data variations could explain the limited impact of pre-processing filters on the diagnostic performances in these studies. Ultimately, although algorithms trained on pre-processed data may perform well in controlled settings, their accuracies in real-time, noisy ECG data in perioperative settings with frequent artifacts remains uncertain.

Moreover, concurrent arrhythmias or ECG abnormalities (e.g. electrolyte imbalances, left bundle branch block) may limit AI interpretation accuracy. Most studies failed to address these major confounders in their ECG datasets, restricting their real-life applicability.

Additionally, there was no discernible indication that either the type of ECG lead or the collection setting significantly influenced AI diagnostic performance—although interpretation is limited by the small number of out-patient (*n*=6) and smart watch (*n*=3) data compared with in-patient data (*n*=178).

Deep learning models, although potentially offering improved performance, require extensive datasets owing to their complexity. Current data availability is constrained by labelling errors (e.g. a 1.86% error rate in the PTB-XL dataset^36^) and the lack of labelled records, which impact model training and evaluation. Data augmentation and self-supervised learning (SSL) may help overcome these limitations by enabling pattern recognition without extensive labelling; however, they may risk amplifying any biases that pre-exist in datasets^37^.

### Current limitations and knowledge gaps

The current limitations and knowledge gaps are summarised as follows in Table 3. Firstly, most AI algorithms were trained on curated, open-source ECGs, limiting their generalisability to high-risk surgical populations and noisy perioperative environments. Secondly, perioperative myocardial ischaemia often results from oxygen supply–demand mismatch, producing ECG patterns distinct from acute MI in non-surgical settings, which complicates algorithm applicability. Thirdly, most studies used physician ECG interpretation as the reference standard rather than definitive diagnosis of myocardial ischaemia or MI, potentially introducing bias. Lastly, integrating AI-based diagnostic tools into perioperative workflows presents technical challenges. Wearable devices often require deliberate positioning, restricting practicality in perioperative settings. With more than 60% of perioperative MI cases being asymptomatic^10^ and frequently occurring at night,^14^ more advanced, clinically validated AI algorithms are required for critical perioperative applications.

**Table 3:** Current limitations and knowledge gaps in implementing AI for ECG interpretation of myocardial ischaemia and infarction in perioperative and remote home monitoring settings. AI, artificial intelligence; NSTEMI, non-ST-segment elevation myocardial infarction; STEMI, ST-segment elevation myocardial infarction.

Limitations and knowledge gaps	Explanation
Limited or unclear ECG database for AI algorithm development	Most existing AI algorithms were developed using a few open-source ECG databases, resulting in limited external validity. Additionally, many studies do not specify the ECG sources used for their AI algorithm creation
Lack of ECG sources from perioperative and remote home monitoring settings	None of the open-source ECG databases contain ECGs from perioperative or remote home monitoring settings. Moreover, these ECGs are typically pre-processed to remove all artifacts and contain only one diagnosis per ECG strip. Consequently, AI algorithms may struggle to diagnose myocardial ischaemia or infarction in noisy environments or in patients with co-existing conditions that affect ECG waveforms, such as electrolyte imbalances, potentially leading to false alarms
Unclear and unaligned target conditions	Many AI algorithms do not clearly define their target conditions. Most studies (*n*=58) list myocardial infarction as the target condition, some specify STEMI (*n*=16), whereas others vaguely mention ischaemic changes (*n*=11) or ST changes (*n*=7). Only one study specifically targets NSTEMI.
	For perioperative and remote home monitoring after surgery, the target conditions should include any myocardial ischaemic changes or myocardial infarction, typically presenting as ST depression or T wave inversions in the lateral ventricular wall. In contrast, AI algorithms designed for MI detection in cardiology or emergency medicine often focus on anterior, inferior, and posterior wall MI. Because the target conditions in previous studies may not align with the perioperative use, the reported diagnostic accuracies are uncertain
Unaligned ECG lead monitoring configuration	The most common data type used in these studies was 12-lead ECG records (*n*=67), followed by two-lead ECG records (*n*=13) and one-lead ECG records (*n*=6). Other ECG records included three-lead, 15-lead, nine-lead, and six-lead configurations (combined *n*=7). In perioperative settings, where myocardial ischaemia and infarction commonly occur in the lateral ventricular wall, the standard monitoring strategy is two-lead ECG (leads II and V5). Therefore, diagnostic accuracy from the literature may not be directly transferable to the perioperative setting
Unaligned reference standards	Most studies used physician interpretation of the same ECG strip as the reference standard. Consequently, the reported diagnostic accuracies reflect how well the AI algorithm mimics physician interpretation, not the accurate diagnosis of MI. Thus, the translation of ECG interpretation accuracy to the accurate diagnosis of MI in patients is uncertain. Importantly, because most studies use existing ECG databases for development, they often lack actual event rates of myocardial ischaemia or infarction in their datasets
Static ECG interpretation	Existing AI algorithms are designed to interpret a single ECG strip. However, in clinical practice, physicians typically compare the current ECG with previous ones and make diagnoses based on patient history, pre-existing risk factors, and the surgical course. AI has the potential to integrate all this information for dynamic interpretation throughout the patient's journey, but this integration has yet to be developed
Lack of clinical validation	None of the AI algorithms have been tested in clinical settings, so their actual performance remains unknown

### Future directions

AI has garnered significant interest in perioperative medicine, extending beyond ECG monitoring. In a systematic review by Bellini and colleagues,^38^ AI was highlighted across various perioperative fields, including predicting risk of perioperative mortality,^39^ cardiovascular complications,^40^, ^41^, ^42^ and acute kidney injury after major surgeries, such as cardiac surgery or total knee arthroplasty.^43^^,^^44^ Many of these studies relied on preoperative variables, including patient characteristics, medical history, and laboratory values, to estimate risks. Notably, six studies explored real-time prediction using real-time data feeding into AI algorithms for early warning systems, specifically targeting overly deep sedation, hypotension, hypoxemia, and bradycardia.^38^ These studies used machine learning techniques such as gradient boosting, random forest, and logistic regression, with less frequent use of neural networks such as ANNs or SVMs. None of the real-time studies utilised advanced AI methods such as CNNs or ResNet, commonly used for ECG interpretation. This gap emphasises the need to develop high-accuracy AI tools tailored to real-time, noisy perioperative ECGs across intraoperative, postoperative, or remote settings. Such advancements could enhance monitoring capabilities and potentially improve patient outcomes.

### Limitations

Primarily, our interpretation of the results does not include quantitative meta-analysis because of insufficient reporting of the primary studies. However, we can identify patterns and provide context to the existing literature. The systematic review on non-ECG studies by Bellini and colleagues^38^ reinforces our sentiments, concluding that the current research landscape is heterogeneous in terms of settings and the algorithms evaluated, and this diversity makes uniform evaluation and conducting a meta-analysis impossible. Additionally, the practical aspects of ECG reporting, such as the number of segments, number of subjects, use of pre-processing filters, and the optimal number of ECG leads, varied significantly across studies. These inconsistencies may have also impacted how findings were incorporated into our review. The variability in reference standards may also contribute to heterogeneity in model development and performance.

Furthermore, another limitation is the limited inclusion of grey literature and proprietary datasets commonly found in technology and industry domains, despite our comprehensive search across databases in four areas including clinical, engineering, and allied health. It is likely that additional relevant sources remain inaccessible because of commercial restrictions or unpublished status. This highlights the importance of fostering a multidisciplinary approach to AI integration in healthcare. While medical leadership must continue to guide AI tool development, evaluation, and implementation, the meaningful inclusion of engineering expertise and industry resources are essential to fully realise the potential of AI in perioperative care. Equally important is the adoption of AI-specific reporting frameworks such as the DECIDE-AI guideline^45^ for early-stage clinical evaluation of AI-based decision support systems. Future ECG-AI studies should align with these AI-specific guidelines to improve transparency, reproducibility, and credibility.

### Conclusions

This scoping review identifies a translational gap in AI algorithm development in its ability to be used in a perioperative context, noticed by (1) a lack of a perioperative ECG database to develop and test AI algorithms for perioperative and remote home monitoring applications, (2) a lack of clinical testing in a more realistic noisy ECG collection environment, and (3) a lack of development and integration of AI algorithms into the wearable device for remote home monitoring setting. By addressing these knowledge gaps, we believe a more dependable perioperative and remote MI monitoring strategy for surgical patients can be developed that might enable early hospital discharge.

## Authors’ contributions

Study design: AK, MC, MK, ADCC, HY, JC

Data collection: AK, AI, JC

Data analysis: AK, JC

Writing of the first draft of the manuscript: AK, JC

Editing of the manuscript: MC, MK, ADCC, HY

Systematic search: AI

## Funding

Canadian Anesthesia Research Foundation (CARF); University of Western Ontario.

## Declaration of interest

The authors declare that they have no conflicts of interest.

## Figures and Tables

**Figure:**