# Supplementary material for: Artificial intelligence for electrocardiographic diagnosis of perioperative myocardial ischaemia: a scoping review
Source: Br J Anaesth. 2025 Jul 4;135(3):561–70. doi: 10.1016/j.bja.2025.05.037 (PMC12489347; doi:10.1016/j.bja.2025.05.037)
Supplement: Multimedia component 1 [file mmc1.pdf]

Artificial Intelligence for ECG diagnosis of Perioperative Myocardial Ischemia: A Scoping Review

Supplementary Information

Content

Content	Page
Supplementary Figure 1. The number of publications related to AI uses for ECG interpretation from 1995 to 2023	3
Supplementary Table 1. Comparison of definitions of diagnostic performance metrics used in Computer/Engineering literature and Clinical Epidemiology literature	4
Supplementary Table 2. Summary of commonly used open-source ECG databases utilized for generating AI algorithms for myocardial ischemia detection	5
Supplementary Table 3. Study Characteristics	6
Supplementary Table 4. Detailed characteristics of AI algorithms and comparison used in primary studies	17
Supplementary Table 5. Detailed Diagnostic Test Accuracy of AI algorithms of primary studies	26
Supplementary Information 1. Search strategy	34
Supplementary Information 2. Citations of all included studies	41
Supplementary Information 3. Detailed Description of AI Technology used in ECG Interpretation.	51

Supplementary Figure 1. The number of publications related to AI uses for ECG interpretation from 1995 to 2023

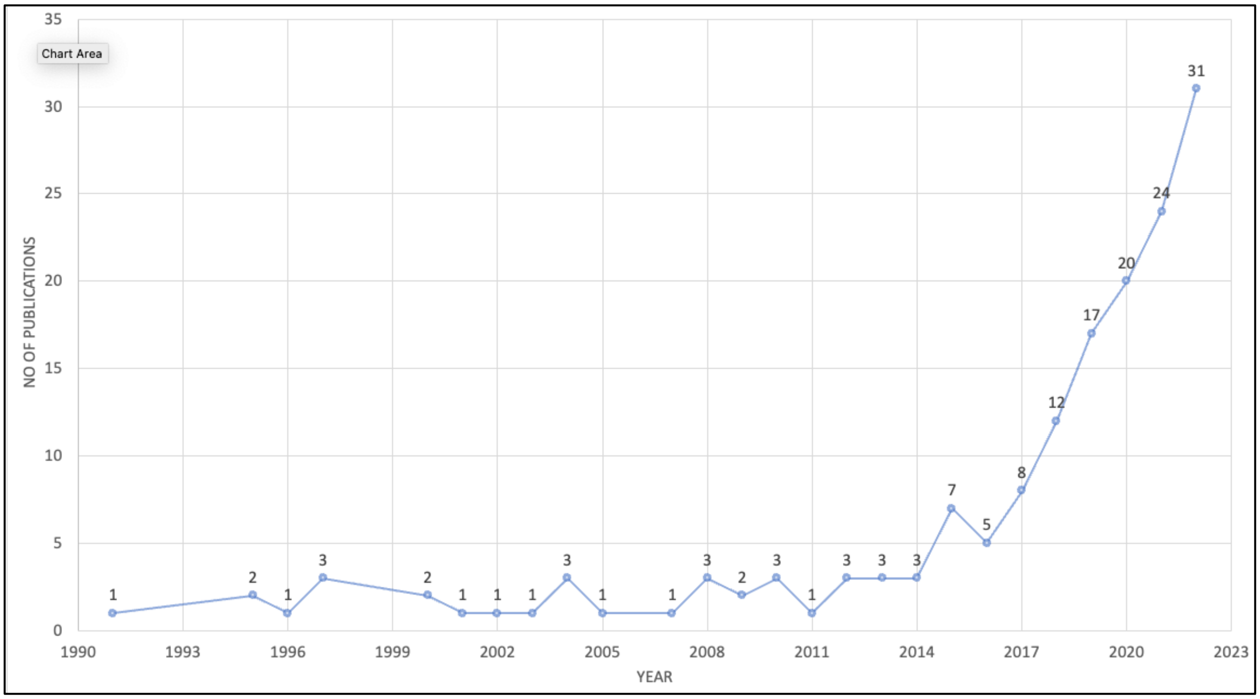

Supplementary Table 1. Comparison of definitions of diagnostic performance metrics used in Computer/Engineering literature and Clinical Epidemiology literature.

	Formulae	Computer science interpretation	Epidemiology interpretation
**Recall or Sensitivity (True Positive Rate)**	= TP / TP + FN	Recall refers to the proportion of actual positive cases that are correctly identified by the algorithm.	Sensitivity refers to the ability of a diagnostic test to correctly identify individuals with the disease or condition.
**Specificity (True Negative Rate)**	= TN / TN + FP	Specificity measures the proportion of actual negative cases that are correctly identified by the algorithm	Specificity refers to the ability of a diagnostic test to correctly identify individuals without the disease or condition
**Precision (Positive Predictive Value)**	= TP/ TP + FP	Precision refers to the proportion of true positive predictions among all positive predictions made by the algorithm.	Positive predictive value refers to the proportion of individuals correctly identified as having the disease among all individuals identified as positive by the test.
**Accuracy**	= TP + TN / Total prediction	Accuracy measures the overall correctness of the predictions made by the algorithm.	Accuracy represents the overall correctness of the diagnostic test results.
**F1 Score**	= 2x (precision * recall)/ (precision + recall)	The F1 score is the harmonic mean of precision and recall, providing a single metric that balances both precision and recall	-
**AUC or AUROC**	N/A	AUC represents the ability of a diagnostic test to discriminate between individuals with and without a particular disease or condition. The higher AUC value, the better discriminatory ability of the test.	

Abbreviation: AUC: Area under the curve, AUROC: Area under the Receiver operating curve. The ROC curve plots the true positive rate (sensitivity) against the false positive rate (1 - specificity) at various threshold. NA: not applicable.

<u>Supplementary Table 2. Summary of commonly used open-source ECG databases utilized for generating AI algorithms for myocardial ischaemia detection</u>

	No. of lead	No. of ECGs	No. of patients	Length	Annotation	Target conditions
**PTB-XL**	12-lead	21799 ECGs	18869	10s	2 cardiologists	71 different ECG statements
**European ST-T Database**	Ambulatory ECG	NS; 2 hrs duration for each ECG.	79 (70 males and 8 females)	30s to several minutes	2 cardiologists; disagreements were resolved with 3rd cardiologists	ST and T-wave changes; 367 episodes of ST segment change, and 401 episodes of T-wave change
**PhysioNet/BIH ST change Database**	Exercise stress tests	28 ECGs	NS	Variable	NS	Mostly ST depression; 5 records of ST elevations were from long-term ECG recordings

Abbreviation: NS: Not specified

PhysioNet contains multiple free-accessed ECG databases.

Supplementary Table 3. Study Characteristics

Study (1st Author)	Year	Country	Study Design	Number of patients in the Derivation cohort (n)	Number of Patients in the Validation Cohort (n)	Age (mean (SD))	Female (%)	Known CAD (%)	Known LBBB (%)	Known AF (%)	Previous MI (%)	ECG Sources/Inclusion criteria	Type of ECG	Type of Database	Preprocessing	Type of Preprocessing
Baxt	1991	USA	R	351	N/A	51.6-52.4	42	N/A	N/A	N/A	10.9	In-Patient	12-lead ECG		No	
Bosnjak et al.	1995	Venezuela	R	9		N/A	N/A	N/A	N/A	N/A	N/A	In-Patient	2-lead ECG	European Society of Cardiology ST-T Database	Yes	Multistate Kalman filter
Silipo et al.	1995	Italy	R	105 recordings	100 recordings	N/A	N/A	N/A	N/A	N/A	N/A	In-Patient	12-lead ECG	European Society of Cardiology ST-T Database	No	
Kaiser et al.	1996	Germany	R	404	292	N/A	N/A	N/A	N/A	N/A	N/A	In-Patient	12-lead ECG	N/A	No	
Polak et al.	1997	Canada	R	100	50	N/A	54	N/A	N/A	N/A	N/A	In-Patient	12-lead ECG		No	
Heden et al.	1997	Sweden	R	1120 recordings		71 (12)	38	N/A	N/A	N/A	N/A	In-Patient	12-lead ECG		No	
Maglaveras et al.	1997	Macedonia	R	160	N/A	N/A	N/A	N/A	N/A	N/A	N/A	In-Patient	12-lead ECG	European Society of Cardiology ST-T Database	Yes	Denoising
Bezerianos et al.	2000	Greece	R	79		N/A	N/A	N/A	N/A	N/A	N/A	In-Patient	2-lead ECG	European Society of Cardiology ST-T Database	Yes	Bazett formula (ST-T segment preprocessor)
Garcia et al.	2000	Spain	R	368 segment episodes (no patient#)	250	N/A	N/A	N/A	N/A	N/A	N/A	In-Patient	2-lead ECG	European Society of Cardiology ST-T Database	Yes	Baseline wander attenuation, rejection of noisy beats
Papaloukas et al.	2001	Greece	R	90		N/A	N/A	N/A	N/A	N/A	N/A	In-Patient	2-lead ECG	European Society of Cardiology ST-T Database	Yes	Baseline wander attenuation, noise elimination
Baxt et al.	2002	USA	R	361		61.1 (13.9)	45.9	50.7	3.3	N/A	21.9	In-Patient	12-lead ECG		No	
Langley et al.	2003	UK	R	N/A	N/A	N/A	N/A	N/A	N/A	N/A	N/A	In-Patient	12-lead ECG	Physionet ST Changes Database	No	
Haraldsson et al.	2004	Sweden	R	1499	739	70.8 (12.4)	48.4	N/A	N/A	N/A	N/A	In-Patient	12-lead ECG		Yes	QRS duration and amplitude shift preprocessing parameters

Heden et al.	2004	Sweden	R	738	369	N/A	N/A	N/A	N/A	N/A	N/A	In-Patient	12-lead ECG		No	
Andreao et al.	2004	Brazil	R	120 episodes	N/A	N/A	N/A	N/A	N/A	N/A	N/A	In-Patient	12-lead ECG	European Society of Cardiology ST-T Database	No	
Yang et al.	2005	Scotland	R	100	108	N/A	N/A	N/A	N/A	N/A	N/A	In-Patient	12-lead ECG		No	
Mohebbi et al.	2007	Iran	R	48	N/A	N/A	N/A	N/A	N/A	N/A	N/A	In-Patient	12-lead ECG	European Society of Cardiology ST-T Database	Yes	Pan-Tompkins Algorithm
Mohebbi et al.	2007	Iran	R	12000 beats	6047 beats	N/A	N/A	N/A	N/A	N/A	N/A	In-Patient	12-lead ECG	European Society of Cardiology ST-T Database	Yes	Denoising, Pan-Tompkins Algorithm, segmentation
Olsson et al.	2008	Sweden	R	120		77.4 (9.1)	38.33	N/A	6.8	N/A	N/A	In-Patient	12-lead ECG		No	
Zizzo et al.	2008	United Kingdom	R	48		N/A	N/A	N/A	N/A	N/A	N/A	In-Patient	2-lead ECG	European Society of Cardiology ST-T Database	Yes	Denoising filter
Afsar et al.	2008	Pakistan	R	90	N/A	N/A	N/A	N/A	N/A	N/A	N/A	In-Patient	12-lead ECG	European Society of Cardiology ST-T Database	Yes	Denoising
Jayachandran et al.	2009	India	R	8	2	60	0	N/A	N/A	N/A	N/A	In-Patient	12-lead ECG	N/A	Yes	Discrete Wavelet Transform
Dranca et al.	2009	Spain	R	80		N/A	N/A	N/A	N/A	N/A	N/A	In-Patient	3-lead ECG	LTST Database	Yes	ECGPUWAVE tool
Faganeli et al.	2010	Slovenia	R	86		N/A	N/A	N/A	N/A	N/A	N/A	In-Patient	3-lead ECG	LTST Database	Yes	Derivation of the Karhunen-Loeve Transform, measurement of ST
Khoshnoud et al.	2010	Iran	R	90 signals	30 signals	N/A	N/A	N/A	N/A	N/A	N/A	In-Patient	12-lead ECG	European Society of Cardiology ST-T Database	Yes	Multiresolution Wavelet Transform
Bakhshipour et al.	2010	Iran	R	90	N/A	N/A	N/A	N/A	N/A	N/A	N/A	In-Patient	12-lead ECG	European Society of Cardiology ST-T Database	Yes	Wavelet Transform
Sankari et al.	2011	USA	R	60	20	N/A	N/A	N/A	N/A	N/A	N/A	In-Patient	12-lead ECG	N/A	No	
Forberg et al.	2012	Sweden	R	3000		70.1(0.6)	45	N/A	N/A	N/A	N/A	In-Patient	12-lead ECG		No	
Dhawan et al.	2012	USA	R	201		57.2(13.2)	34.75	N/A	N/A	N/A	N/A	Out-Patient	12-lead ECG		Yes	ECG extraction preprocessing filter

Park et al.	2012	South Korea	R	90		N/A	N/A	N/A	N/A	N/A	N/A	In-Patient	2-lead ECG	European Society of Cardiology ST-T Database	Yes	DB-8 Wavelet Filtering
Wilson et al.	2013	USA	R	305		56.8(15.8)	35.6	N/A	N/A	N/A	N/A	In-Patient	12-lead ECG		No	
Keshtkar et al.	2013	Iran	R	100		N/A	N/A	N/A	N/A	N/A	N/A	In-Patient	15-lead ECG	PTC Dataset	Yes	High pass filter; Low pass bidirectional Butterworth filters
Pelaez et al.	2013	Spain	R	800	N/A	N/A	N/A	N/A	N/A	N/A	N/A	In-Patient	12-lead ECG	Gen Med Database	Yes	Wavelet Transform
Liu et al.	2014	China	R	148		N/A	N/A	N/A	N/A	N/A	N/A	In-Patient	12-lead ECG	PTB Dataset	Yes	Discrete Wavelet Transform
Finlay et al.	2014	USA	R	90 records	90 records	57.3 - 59	37.8	N/A	N/A	N/A	N/A	In-Patient	12-lead ECG		No	
Li et al.	2014	Japan	R	16318 beats	23387 beats	N/A	N/A	N/A	N/A	N/A	N/A	In-Patient	12-lead ECG	European Society of Cardiology ST-T Database	No	
Treskes et al.	2015	Netherlands	R	84		57 (16.6)	36	N/A	N/A	N/A	N/A	In-Patient	12-lead ECG		No	
Kora et al.	2015	India	R	148		N/A	N/A	N/A	N/A	N/A	N/A	In-Patient	1-lead ECG	PTB Dataset	Yes	Sgolay FIR smoothing filter
Bansal et al.	2015	India	R	47		N/A	N/A	N/A	N/A	N/A	N/A	In-Patient	2-lead ECG	MIT-BIH ST Change Database	Yes	Elliptical High-Pass filter, Butterworth low-pass filter
Rad et al.	2015	Iran	R	4		N/A	N/A	N/A	N/A	N/A	N/A	In-Patient	2-lead ECG	European Society of Cardiology ST-T Database	No	
Mohanta et al.	2015	Haryana	R	367 episodes	N/A	30-84; 55-71	10.3	N/A	N/A	N/A	N/A	In-Patient	12-lead ECG		Yes	Denoising
Bhaskar	2015	USA	R	224 records	113 records	N/A	N/A	N/A	N/A	N/A	N/A	In-Patient	12-lead ECG	PTB Dataset	Yes	Bandpass filter, Pan-Tompkins Algorithm
Yu et al.	2016	Taiwan	R	78	10	N/A	N/A	N/A	N/A	N/A	N/A	In-Patient	2-lead ECG	European Society of Cardiology ST-T Database	Yes	Discrete Wavelet Transform
Garvey et al.	2016	USA	R	151		60.2	20.7	N/A	N/A	N/A	N/A	Out-Patient	12-lead ECG		No	
Tseng et al.	2016	Taiwan	R	43	5	N/A	N/A	N/A	N/A	N/A	N/A	In-Patient	2-lead ECG	European Society of Cardiology ST-T Database	Yes	Baseline wander removal
Wu et al.	2016	China	R	148	N/A	N/A	N/A	N/A	N/A	N/A	N/A	In-Patient	12-lead ECG	PTB Dataset	No	

Murthy et al.	2016	India	R	2424 beats	280	N/A	N/A	N/A	N/A	N/A	N/A	In-Patient	12-lead ECG	European Society of Cardiology ST-T Database	Yes	Denoising, segmentation
Firoozabadi et al.	2017	USA	R	1171 recordings		N/A	N/A	N/A	N/A	N/A	N/A	Holter	12-lead ECG		No	
Liu et al.	2017	China	R	368		N/A	N/A	N/A	N/A	N/A	N/A	In-Patient	12-lead ECG	PTB Dataset	Yes	Beat segmentation algorithm; Fuzzy Information Granulation filter
Murthy	2017	India	R	404 beats	280 beats	N/A	N/A	N/A	N/A	N/A	N/A	In-Patient	12-lead ECG	European Society of Cardiology ST-T Database	Yes	Soft wavelet-based thresholding denoising technique
Kora	2017	India	R	1800 beats	1006 beats	N/A	N/A	N/A	N/A	N/A	N/A	In-Patient	12-lead ECG	MIT-BIH ST Change Database	No	
Kumar et al.	2017	India	R	148	N/A	N/A	N/A	N/A	N/A	N/A	N/A	In-Patient	1-lead ECG	PTB Dataset	Yes	DB-6, Pan-Tompkins Algorithm
Reasat et al.	2017	Bangladesh	R	549 records	N/A	N/A	N/A	N/A	N/A	N/A	N/A	In-Patient	3-lead ECG	PTB Dataset	Yes	Baseline wander, Savitzky-Golay Filter
Dohare et al.	2017	India	R	549 records	N/A	N/A	N/A	N/A	N/A	N/A	N/A	In-Patient	12-lead ECG	PTB Dataset	No	
Sharma et al.	2017	India	R	3240 segments	N/A	N/A	N/A	N/A	N/A	N/A	N/A	In-Patient	12-lead ECG	PTB Dataset	Yes	Wavelet Decomposition, Denoising, Savitzky-Golay Filter
Sharma et al.	2018	India	R	45656 epochs	5072 epochs	N/A	25.5	N/A	N/A	N/A	N/A	In-Patient	1-lead ECG	PTB-XL Dataset	Yes	DB-6 Wavelet Function, Pan Tompkins Algorithm
Xiao et al.	2018	USA	R	20	15	N/A	N/A	N/A	N/A	N/A	N/A	In-Patient	2-lead ECG	LTST Database	No	
De La Fuentes-Cortes et al.	2018	Mexico	R	64	68	38-87	21	N/A	N/A	N/A	N/A	In-Patient	12-lead ECG	PTB-XL Dataset	No	
Wang et al.	2018	China	R	234452 beats	N/A	N/A	N/A	N/A	N/A	N/A	N/A	In-Patient	1-lead ECG	PTB Dataset	Yes	Baseline wander
Chen et al.	2018	China	R	148	N/A	N/A	N/A	N/A	N/A	N/A	N/A	In-Patient	12-lead ECG	PTB Dataset	No	
Xue et al.	2018	China	R	1714	N/A	N/A	N/A	N/A	N/A	N/A	N/A	In-Patient	12-lead ECG		Yes	Denoising

Davydov et al.	2018	Russia	R	179	N/A	N/A	N/A	N/A	N/A	N/A	N/A	In-Patient	12-lead ECG	PTB Dataset	Yes	Signal Segmentation
Liu et al.	2018	China	R	408 records	N/A	N/A	N/A	N/A	N/A	N/A	N/A	In-Patient	12-lead ECG	PTB Dataset	Yes	Pan-Tompkins Algorithm
Diker et al.	2018	Turkey	R	549 records	N/A	N/A	N/A	N/A	N/A	N/A	N/A	In-Patient	12-lead ECG	PTB Dataset	Yes	Power line interference, denoising
Lui et al.	2018	Hong Kong	R	549 records	N/A	N/A	N/A	N/A	N/A	N/A	N/A	In-Patient	12-lead ECG	PTB Dataset	Yes	Savitzky-Golay, baseline wander
Sopic et al.	2018	Switzerland	R	52	N/A	N/A	N/A	N/A	N/A	N/A	N/A	In-Patient	12-lead ECG	PTB Dataset	Yes	FIR band-pass filter, Pan-Tompkins Algorithm
Sadhukhan et al.	2018	India	R	369 records	N/A	N/A	N/A	N/A	N/A	N/A	N/A	In-Patient	12-lead ECG	PTB Dataset	No	
Avila	2019	USA	P	2		60	0	N/A	N/A	N/A	N/A	Smartwatch	3-lead ECG	N/A	No	
Han et al.	2019	China	R	219	149	N/A	N/A	N/A	N/A	N/A	N/A	In-Patient	12-lead ECG	PTB-XL Dataset	Yes	Discrete Wavelet Transform, Pan-Tompkins Algorithm
Strodthoff et al.	2019	Germany	R	127		61(16)	27.56	N/A	N/A	N/A	N/A	In-Patient	12-lead ECG	PTB-XL Dataset	No	
Chowdhury et al.	2019	Qatar	R	28		N/A	N/A	N/A	N/A	N/A	N/A	In-Patient	12-lead ECG	MIT-BIH ST Change Database	Yes	Signal processing and Wavelet Transformation
Wu et al.	2019	Taiwan	R	27	9	63.1 (13.0)	26	68	N/A	N/A	N/A	In-Patient	12-lead ECG		Yes	Preprocessing filter (unspecified)
Liu et al.	2019	China	R	290		57.2	27.9	N/A	N/A	N/A	N/A	In-Patient	12-lead ECG	PTB Dataset	Yes	Dual-Q TQWT denoising method, Wavelet Packet Tensor Decomposition
Liu et al.	2019	China	R	200		N/A	N/A	N/A	N/A	N/A	N/A	In-Patient	12-lead ECG	PTB Dataset	Yes	Pan-Tompkins Algorithm
Han et al.	2019	China	R	165		N/A	N/A	N/A	N/A	N/A	N/A	In-Patient	12-lead ECG	PTB Dataset	Yes	Wavelet denoising method, Pan-Tompkins algorithm
Muhlestein et al.	2019	USA	P	204		59.8 (15.7)	43	N/A	N/A	N/A	N/A	Out-patient/Smartphone ECG	12-lead ECG		No	

Wang et al.	2019	China	R	148		N/A	N/A	N/A	N/A	N/A	N/A	In-Patient	12-lead ECG	PTB Dataset	Yes	Baseline drift removal and wavelet decomposition
Liu et al.	2019	Dalian	R	52		59.3 (11.4)	26.9	N/A	N/A	N/A	N/A	In-Patient	15-lead ECG	PTB Dataset	Yes	High pass and Low Pass FIR filters
Hillinger et al.	2019	Switzerland	P	2486	1306	48-73	32	33	N/A	N/A	24	In-Patient	12-lead ECG		No	
Sharma et al.	2019	India	R	148	N/A	N/A	N/A	N/A	N/A	N/A	N/A	In-Patient	12-lead ECG	PTB Dataset	Yes	Baseline wander, Savitzky-Golay Filter
She et al.	2019	China	R	2109 signals	N/A	N/A	N/A	N/A	N/A	N/A	N/A	In-Patient	8-lead ECG		Yes	Wavelet Transform
Sahu et al.	2019	India	R	27040 segments	27047 segments	N/A	N/A	N/A	N/A	N/A	N/A	In-Patient	12-lead ECG	PTB Dataset	Yes	Baseline wander, Pan-Tompkins Algorithm
Zhang et al.	2019	China	R	148	N/A	N/A	N/A	N/A	N/A	N/A	N/A	In-Patient	1-lead ECG	PTB Dataset	No	
Zhang et al.	2019	China	R	49278 beats	5475 beats	N/A	N/A	N/A	N/A	N/A	N/A	In-Patient	12-lead ECG	PTB Dataset	Yes	Low pass filter, Pan-Tompkins Algorithm
Darmawahyuni et al.	2019	Indonesia	R	549 records	N/A	N/A	N/A	N/A	N/A	N/A	N/A	In-Patient	12-lead ECG	PTB Dataset	No	
Darmawahyuni et al.	2019	Indonesia	R	439 reocrds	110 records	N/A	N/A	N/A	N/A	N/A	N/A	In-Patient	12-lead ECG	PTB Dataset	No	
Zhang et al.	2019	China	R	549 records	N/A	N/A	N/A	N/A	N/A	N/A	N/A	In-Patient	12-lead ECG	PTB Dataset	Yes	Denoising, beat segmentation
Zhang et al.	2019	China	R	549 records	N/A	N/A	N/A	N/A	N/A	N/A	N/A	In-Patient	12-lead ECG	PTB Dataset	Yes	Power line interference, denoising
Feng et al.	2019	China	R	549	N/A	N/A	N/A	N/A	N/A	N/A	N/A	In-Patient	12-lead ECG	PTB Dataset	Yes	Baseline drift, wavelet transform
Baloglu et al.	2019	Turkey	R	427983 records	91711 records	N/A	N/A	N/A	N/A	N/A	N/A	In-Patient	12-lead ECG	PTB Dataset	No	
Tripathy et al.	2019	India	R	549 records	N/A	N/A	N/A	N/A	N/A	N/A	N/A	In-Patient	12-lead ECG	PTB Dataset	No	
Spaccarotella et al.	2020	Italy	P	100		61 (16)	3 3	N/A	N/A	N/A	15 (15)	Smartwatch	9-lead ECG	N/A	No	
Hao et al.	2020	China	R	483		N/A	N/A	N/A	N/A	N/A	N/A	In-Patient	12-lead ECG		No	
Cho et al.	2020	South Korea	R	2081	200	63.8 (12.3)	7 3	N/A	N/A	N/A	N/A	In-Patient	6-lead ECG		No	

							.<br>3									
Brown et al.	2020	Germany	P	1		61	0	N/A	N/A	N/A	N/A	Smartwatch	1-lead ECG		No	
Makimoto et al.	2020	Germany	R	25	25	N/A	N/A	N/A	N/A	N/A	N/A	In-Patient	12-lead ECG	PTB Dataset	Yes	Preprocessing filter (unspecified)
Zhao et al.	2020	China	R	432	185	72.43(13.28)	45.6	N/A	N/A	N/A	N/A	In-Patient	12-lead ECG		Yes	Finite Impuse Response Filter
Fu et al.	2020	China	R	148		N/A	N/A	N/A	N/A	N/A	N/A	In-Patient	12-lead ECG	PTB Dataset	Yes	Denoising, baseline drift removal, data segmentation
Kapfo et al.	2020	India	R	549 records	N/A	55.5-61.6	27.9	N/A	N/A	N/A	N/A	In-Patient	12-lead ECG	PTB-XL Dataset	Yes	Low-frequency baseline wandering filter
Aufderheide et al.	2020	USA	R	285	N/A	62	45.3	N/A	N/A	N/A	29.2	In-Patient	12-lead ECG		No	
Ibrahim et al.	2020	UAE	R	6716	1679	N/A	N/A	N/A	N/A	N/A	N/A	In-Patient	12-lead ECG	ECG-VIEWII Database	Yes	Synthetic Minority Oversampling Technique
Ogrezeanu et al.	2020	Romania	R	90	N/A	N/A	N/A	N/A	N/A	N/A	N/A	In-Patient	12-lead ECG	European Society of Cardiology ST-T Database	No	
Muminov et al.	2020	Uzbekistan	R	652	N/A	N/A	N/A	N/A	N/A	N/A	N/A	In-Patient	12-lead ECG	PTB Dataset	No	
Swain et al.	2020	India	R	148	N/A	N/A	N/A	N/A	N/A	N/A	N/A	In-Patient	12-lead ECG	PTB Dataset	Yes	Baseline wander
Lin et al.	2020	China	R	148	N/A	N/A	N/A	N/A	N/A	N/A	N/A	In-Patient	12-lead ECG	PTB Dataset	Yes	Denoising, Baseline wander
Wang et al.	2020	China	R	6902 records	2958 records	57.2		N/A	N/A	N/A	N/A	In-Patient	12-lead ECG	PTB Dataset	Yes	Low and high frequency filter
Omar et al.	2020	Bangladesh	R	447 records	N/A	N/A	N/A	N/A	N/A	N/A	N/A	In-Patient	12-lead ECG	PTB Dataset	No	
Zhang et al.	2020	China	R	112	110	N/A	N/A	N/A	N/A	N/A	N/A	In-Patient	12-lead ECG	PTB Dataset	No	
Nasimov et al.	2020	Uzbekistan	R	710369 records	N/A	N/A	N/A	N/A	N/A	N/A	N/A	In-Patient	12-lead ECG	ECG-VIEWII Database	No	
Sridhar et al.	2020	India	R	549 records	N/A	N/A	N/A	N/A	N/A	N/A	N/A	In-Patient	12-lead ECG	PTB Dataset	Yes	Discrete Wavelet Transform
Hussein et al.	2021	Iraq	R	118	15	N/A	N/A	N/A	N/A	N/A	N/A	In-Patient	2-lead ECG	MIT Physionet Database	Yes	Finite Impuse Response Filter
Martin et al.	2021	USA	R	48646 records	3465 records	N/A	N/A	N/A	N/A	N/A	N/A	In-Patient	12-lead ECG	PTB-XL Dataset	Yes	Low-pass filter, Moving average filter

Safdarian et al.	2021	Iran	R	290		N/A	38.76	N/A	N/A	N/A	N/A	In-Patient	12-lead ECG	MIT Physionet Database	Yes	Moving average, Kaiser, Butterworth, Median filters
Liu et al.	2021	China	R	290		N/A	N/A	N/A	N/A	N/A	N/A	In-Patient	12-lead ECG	PTB Dataset	Yes	DB-6 Wavelet Filtering
Liu et al.	2021	Taiwan	R	58056	19743	61.8 (13.8)	16.2	24	N/A	N/A	N/A	In-Patient	12-lead ECG		No	
Tadesse et al.	2021	United Kingdom	R	17381		N/A	N/A	N/A	N/A	N/A	N/A	In-Patient	12-lead ECG		No	
He et al.	2021	China	R	113		N/A	N/A	N/A	N/A	N/A	N/A	In-Patient	12-lead ECG	PTB Dataset	Yes	Wavelet denoising method, Pan-Tompkins algorithm
Chen et al.	2021	China	R	15285	6552	67.3 (13)	60.6	N/A	N/A	N/A	N/A	In-Patient	12-lead ECG	PTB-XL Dataset	Yes	Low-pass filter
Cao et al.	2021	China	R	148		N/A	N/A	N/A	N/A	N/A	N/A	In-Patient	12-lead ECG	PTB Dataset	Yes	Normalization
Han et al.	2021	South Korea	R	65305	11524	59 (16.98)	47.1	N/A	N/A	N/A	N/A	In-Patient	12-lead ECG		No	
Chang et al.	2021	Singapore	R	25187	7196	65.1 (17.9)	44	N/A	N/A	N/A	N/A	In-Patient	12-lead ECG		Yes	Preprocessing filter (unspecified)
Jahmunah et al.	2021	Singapore	R	148		N/A	N/A	N/A	N/A	N/A	N/A	In-Patient	1-lead ECG		No	
Gibson et al.	2021	USA	R	3819	436	59	39.3	N/A	N/A	N/A	N/A	In-Patient	1-lead ECG	LATIN Dataset	Yes	Wavelet system, segmentation
Liu et al.	2021	Taiwan	R	3320		N/A	N/A	N/A	N/A	N/A	N/A	Out-Patient	12-lead ECG		No	
Dai et al.	2021	Taiwan	R	51321 segments	8146 segments	N/A	N/A	N/A	N/A	N/A	N/A	In-Patient	12-lead ECG	PTB Dataset	No	
Faramand et al.	2021	USA	R	291	N/A	63 (13)	26	38	N/A	N/A	N/A	In-Patient	12-lead ECG		No	
Fatimah et al.	2021	India	R	549 records	N/A	55.5-61.6	27.9	N/A	N/A	N/A	N/A	In-Patient	1-lead ECG	PTB Dataset	Yes	Baseline wander, Power-Line Interference
Ma et al.	2021	China	R	549 records	N/A	N/A	N/A	N/A	N/A	N/A	N/A	In-Patient	12-lead ECG	PTB Dataset	No	
Zhao et al.	2021	China	R	110 records	50 records	N/A	N/A	N/A	N/A	N/A	N/A	In-Patient	12-lead ECG	PTB Dataset	Yes	Baseline wander
Yadav et al.	2021	India	R	72 records	74 records	N/A	N/A	N/A	N/A	N/A	N/A	In-Patient	12-lead ECG	PTB Dataset	No	
Dey et al.	2021	Bangladesh	R	517 records	N/A	N/A	N/A	N/A	N/A	N/A	N/A	In-Patient	12-lead ECG	PTB Dataset	Yes	Denoising, Pan-Tompkins Algorithm

Sinha et al.	2021	India	R	549 records	N/A	N/A	N/A	N/A	N/A	N/A	N/A	In-Patient	12-lead ECG	PTB Dataset	Yes	High pass filter, beat segmentation
Liu et al.	2021	China	R	549 records	N/A	N/A	N/A	N/A	N/A	N/A	N/A	In-Patient	12-lead ECG	PTB Dataset	Yes	Global Average Pooling
Martin et al.	2021	USA	R	148	N/A	N/A	N/A	N/A	N/A	N/A	N/A	In-Patient	12-lead ECG	PTB Dataset	No	
Choudhary et al.	2021	India	R	549 Records	N/A	N/A	N/A	N/A	N/A	N/A	N/A	In-Patient	12-lead ECG	PTB Dataset	Yes	Moving average filter
Jikui et al.	2021	China	R	41368 samples	9532 samples	N/A	N/A	N/A	N/A	N/A	N/A	In-Patient	12-lead ECG	PTB Dataset	Yes	Denoising, wavelet transform
Biglar et al.	2021	Switzerland	R	668 images	167 images	68(10)	17	N/A	N/A	N/A	44	In-Patient	12-lead ECG		No	
Kim et al.	2022	South Korea	R	32968	80	64.7 (13.8)	21.2	N/A	N/A	N/A	N/A	In-Patient	12-lead ECG	N/A	No	
Sun et al.	2022	China	R	499	290	N/A	N/A	N/A	N/A	N/A	N/A	In-Patient	12-lead ECG	N/A	No	
Chumachenko et al.	2022	Ukraine	R	5486		N/A	N/A	N/A	N/A	N/A	N/A	In-Patient	12-lead ECG	PTB-XL Dataset	Yes	Noise reduction technique (unspecified)
Wu et al.	2022	China	R	276	155	62.8 (14.8)	49.1	N/A	N/A	N/A	N/A	In-Patient	12-lead ECG	N/A	No	
Chen et al.	2022	China	P	275	10	N/A	N/A	N/A	N/A	N/A	N/A	Out-Patient	12-lead ECG	N/A	No	
Uchiyama et al.	2022	Japan	R	124		N/A	N/A	N/A	N/A	N/A	N/A	In-Patient	12-lead ECG	PTB-XL Dataset	No	
Fang et al.	2022	Taiwan	R	118	30	N/A	N/A	N/A	N/A	N/A	N/A	In-Patient	12-lead ECG	PTB Dataset	Yes	Normalization
Kumar et al.	2022	India	R	8	2	55 (5)	0	N/A	N/A	N/A	N/A	In-Patient	1-lead ECG		Yes	High pass filter
Zhao et al.	2022	China	R	377	200	58 (10)	24.14	N/A	N/A	N/A	N/A	In-Patient	12-lead ECG	PTB Dataset	Yes	High pass filter; Wavelet Transform-based filtering
Jahmunah et al.	2022	Singapore	R	200		N/A	N/A	N/A	N/A	N/A	N/A	In-Patient	12-lead ECG	PTB Dataset	Yes	Reverse Kullback-Leibler Divergence Loss
Gaspardone et al.	2022	Italy	R	314	105	N/A	N/A	N/A	N/A (excluded)	N/A	N/A	In-Patient	12-lead ECG		No	
Cao et al.	2022	China	R	140	60	N/A	N/A	N/A	N/A	N/A	N/A	In-Patient	12-lead ECG	PTB Dataset	Yes	Baseline drift removal and segmentation

Barua et al.	2022	Australia	R	150	50	N/A	N/A	N/A	N/A	N/A	N/A	In-Patient	12-lead ECG	MIT Physionet Database	No	
Sraitih et al.	2022	Morocco	R	106		N/A	N/A	N/A	N/A	N/A	N/A	In-Patient	12-lead ECG	PTB Dataset	Yes	Normalization, Segmentation, Hyperparameters Selection
Gustafsson et al.	2022	Sweden	R	1273	545	66	26.3	20.1	N/A	19.9	8.4	In-Patient	12-lead ECG	PTB-XL Dataset	Yes	Baseline wander removal
Choi et al.	2022	South Korea	R	55451	11883	61.3 (10.5)	22.6	N/A	N/A	N/A	N/A	In-Patient	12-lead ECG		No	
Hammad et al.	2022	Egypt	R	12040	5242	N/A	N/A	N/A	N/A	N/A	N/A	In-Patient	12-lead ECG	PTB-XL Dataset	Yes	Noise band-pass filter
Jahmunah et al.	2022	Singapore	R	118	30	N/A	27	N/A	N/A	N/A	N/A	In-Patient	12-lead ECG	PTB Dataset	Yes	DB-6 Wavelet Filtering
Wu et al.	2022	China	R	377		604 (13.2)	18.3	N/A	0.2	1	N/A	In-Patient	12-lead ECG		Yes	Wavelet decomposition
Choi et al.	2022	South Korea	R	187		64.2 (14.7)	24.6	N/A	N/A	N/A	N/A	In-Patient	12-lead ECG		No	
Saradhi et al.	2022	India	R	240 records	N/A	N/A	N/A	N/A	N/A	N/A	N/A	In-Patient	12-lead ECG	Mendeley Dataset	Yes	Denoising
Pan et al.	2022	China	R	4369 records	N/A	N/A	N/A	N/A	N/A	N/A	N/A	In-Patient	12-lead ECG	PTB Dataset	Yes	Resampling
Kavak et al.	2022	Taiwan	R	270 signals	131 signals	N/A	N/A	N/A	N/A	N/A	N/A	In-Patient	12-lead ECG	Physionet ST Changes Database	Yes	Denoising
Saha	2022	India	R	226	56	N/A	N/A	N/A	N/A	N/A	N/A	In-Patient	12-lead ECG	PTB Dataset	No	
He et al.	2022	China	R	123	42	N/A	N/A	N/A	N/A	N/A	N/A	In-Patient	12-lead ECG	PTB Dataset	Yes	Denoising, hearbeat segmentation
Rajakumar et al.	2022	India	R	N/A	N/A	N/A	N/A	N/A	N/A	N/A	N/A	In-Patient	12-lead ECG	MIT-BIH ST Change Database	Yes	Denoising, segmentation
Ma et al.	2022	China	R	549 records	N/A	N/A	N/A	N/A	N/A	N/A	N/A	In-Patient	12-lead ECG	PTB Dataset	Yes	Denoising, low pass filter
Xu et al.	2022	China	R	33415 records	10443 records	N/A	N/A	N/A	N/A	N/A	N/A	In-Patient	12-lead ECG	PTB Dataset	Yes	Low frequency denoising filter
Mirza et al.	2022	Indonesia	R	384 records	82 records	N/A	N/A	N/A	N/A	N/A	N/A	In-Patient	12-lead ECG	PTB Dataset	Yes	Denoising
Han et al.	2022	China	R	7055	N/A	63.7 (16.4)	43.5	N/A	N/A	N/A	N/A	In-Patient	12-lead ECG		No	
Elmannai et al.	2022	Saudi Arabia	R	11642	2910	N/A	N/A	N/A	N/A	N/A	N/A	In-Patient	12-lead ECG	PTB Dataset	No	
Li et al.	2022	China	R	8377 records	N/A	N/A	N/A	N/A	N/A	N/A	N/A	In-Patient	1-lead ECG	PTB Dataset	No	
Rai et al.	2022	India	R	79358 beats	24800 beats	N/A	N/A	N/A	N/A	N/A	N/A	In-Patient	12-lead ECG	PTB Dataset	No	Filtering, Segmentation

Tseng et al.	2023	Taiwan	R	269	115	60.8 (12.3)	11.1	N/A	N/A (excluded)	N/A	13% (6.3)	In-Patient	12-lead ECG	N/A	Yes	Short-Time Fourier Transform, Continuous Wavelet Transform
Park et al.	2023	South Korea	R	578	145	68	35	69	N/A	N/A	N/A	In-Patient	12-lead ECG	N/A	No	
Olsson de Capretz et al.	2023	Sweden	R	4761	2379	59.1 (18.9)	47.3	N/A	N/A	N/A	N/A	In-Patient	12-lead ECG		Yes	Glasgow Algorithm
Al-Zaiti et al.	2023	USA	R	4026	3287	59(16)	47	23	5.9	2.3	24	In-Patient	12-lead ECG		Yes	Noise, artifact, baseline wander removal filter (unspecified)
Xiao et al.	2023	USA	R	4389 recordings	549 recordings	62	48	N/A	N/A	N/A	N/A	In-Patient	12-lead ECG	PTB-XL Dataset	No	
Qin et al.	2023	China	R	359 pieces	342 pieces	30-75	N/A	N/A	N/A	N/A	N/A	In-Patient	12-lead ECG		Yes	One-Hot coding
Xiong et al.	2023	China	R	148	N/A	N/A	N/A	N/A	N/A	N/A	N/A	In-Patient	12-lead ECG	PTB Dataset	Yes	Baseline wander
Rajput et al.	2023	India	R	212	91	N/A	N/A	N/A	N/A	N/A	N/A	In-Patient	12-lead ECG	Kaggle Database	No	
Firoz et al.	2023	Bangladesh	R	294	37	57.2	27.9	N/A	N/A	N/A	N/A	In-Patient	12-lead ECG	PTB Dataset	No	
Han et al.	2023	China	R	17212 records	N/A	N/A	N/A	N/A	N/A	N/A	N/A	In-Patient	12-lead ECG	PTB-XL Dataset	Yes	Wavelet Denoising, Pan-Tompkins Algorithm
Bhaskarpandit et al.	2023	India	R	500 records	N/A	N/A	N/A	N/A	N/A	N/A	N/A	In-Patient	12-lead ECG	Mendeley Dataset	Yes	Multiscale decomposition
Moghadam et al.	2023	Iran	R	392 records	N/A	N/A	N/A	N/A	N/A	N/A	N/A	In-Patient	12-lead ECG	PTB Dataset	Yes	Baseline wander, denoising

Abbreviation: N/A: Not available, USA: United States, R: Retrospective, P: Prospective, CAD: Coronary artery disease, LBBB: Left bundle branch block, AF: atrial fibrillation, MI: myocardial infraction, ECG: electrocardiograph

Supplementary Table 4. Detailed characteristics of AI algorithms and comparison used in primary studies.

Study (1st author)	Year	Target Condition	Reference Standard	Outcomes and Event Rates	Comparator(s)	Tested AI	Traditional Machine Learning	Signal Processing	Neural Network	Hybrid Network	Others	Details of AI technique used*
Baxt	1991	MI	Traditional ECG reading	N/A	1. Commercial ECG system 2. ECG manually annotated by cardiologists	Neural Network	N	N	Y	N	N	ANN
Bosnjak et al.	1995	Ischemic change	Traditional ECG reading	N/A	1. Commercial ECG system 2. ECG manually annotated by cardiologists	Signal Processing	N	Y	N	N	N	Kalman filter
Silipo et al.	1995	MI	Traditional ECG reading	N/A	1. Commercial ECG system 2. ECG manually annotated by cardiologists	Algorithm-based	N	N	N	N	Y	KLT Transform
Kaiser et al.	1996	MI, LVH	Coronary angiograph, ECG	Anterior, Inferior, Lateral MI= 429/800	1. Commercial ECG system	Algorithm-based	N	N	N	N	Y	Learning of Rules
Polak et al.	1997	Ischemic change	Traditional ECG reading	150/1367 (ischemic)	1. Commercial ECG system 2. ECG manually annotated by cardiologists	Algorithm-based	N	N	N	N	Y	Adaptic logic network algorithm
Heden et al.	1997	MI	Traditional ECG reading	N/A	1. Commercial ECG system 2. ECG manually annotated by cardiologists	Neural Network	N	N	Y	N	N	ANN
Maglaveras et al.	1997	MI	Traditional ECG reading	N/A	1. Commercial ECG system 2. ECG manually annotated by cardiologists	Neural Network	N	N	Y	N	N	Backpropagation NN
Bezerianos et al.	2000	ST changes	Traditional ECG reading	N/A	N/A	Algorithm-based	N	Y	N	N	Y	Self-organizing maps algorithm, Radical Basis Function (RBF) layer
Garcia et al.	2000	ST changes	Traditional ECG reading	N/A	1. Commercial ECG system 2. ECG manually annotated by cardiologists	Algorithm-based	N	N	N	N	Y	Root Mean Square Detection Algorithm
Papaloukas et al.	2001	Ischemic change	Traditional ECG reading	N/A	1. Commercial ECG system 2. ECG manually annotated by cardiologists	Neural Network	N	Y	Y	N	N	ANN, Bayesian regularisation
Baxt et al.	2002	Ischemic change	Troponin, Traditional ECG reading	318/2204 (14.4%- myocardial ischemia)	1. Commercial ECG system 2. ECG manually annotated by cardiologists	Neural Network	N	N	Y	N	N	ANN
Langley et al.	2003	MI	Traditional ECG reading	N/A	1. Commercial ECG system 2. ECG manually annotated by cardiologists	Algorithm-based	N	N	N	N	Y	Ischemic ST change algorithm
Haraldsson et al.	2004	MI	Traditional ECG reading	N/A	1. Commercial ECG system 2. ECG read by cardiologists	Neural Network	N	Y	Y	N	N	ANN, Hermite representation
Heden et al.	2004	MI	Traditional ECG reading	N/A	1. Commercial ECG system 2. ECG manually annotated by cardiologists	Neural Network	N	N	Y	N	N	ANN
Andreao et al.	2004	MI	Traditional ECG reading	N/A	1. Commercial ECG system 2. ECG manually annotated by cardiologists	Algorithm-based	N	N	N	N	Y	Hidden Markov Model Algorithm
Yang et al.	2005	MI	Traditional ECG reading	N/A	1. Commercial ECG system 2. ECG manually annotated by cardiologists	Neural Network	N	Y	Y	N	N	ANN, Glasgow Program, Modified Small Inferior Q criteria

Mohebbi et al.	2007	ST Changes	Traditional ECG reading	N/A	1. Commercial ECG system 2. ECG manually annotated by cardiologists	Algorithm-based	N	N	N	N	Y	ST Algorithm
Mohebbi et al.	2007	MI	Traditional ECG reading	N/A	1. Commercial ECG system 2. ECG manually annotated by cardiologists	Traditional Machine Learning	Y	N	N	N	N	SVM
Olsson et al.	2008	MI	Traditional ECG reading	120/518 (MI)	1. Commercial ECG system 2. ECG manually annotated by cardiologists	Neural Network	N	N	Y	N	N	ANN
Zizzo et al.	2008	Ischemic change	Traditional ECG reading	N/A	1. Commercial ECG system 2. ECG manually annotated by cardiologists	Algorithm-based	N	N	N	N	Y	New ECG monitor algorithm- temporal and spatial analysis
Afsar et al.	2008	MI	Traditional ECG reading	N/A	1. Commercial ECG system 2. ECG manually annotated by cardiologists	Algorithm-based	N	N	N	N	Y	Principal Component Analysis Algorithm
Jayachandran et al.	2009	MI	Traditional ECG reading	N/A	1. Commercial ECG system, 2. ECG read by cardiologist	Algorithm-based	N	N	N	N	Y	Discrete Wavelength Transform
Dranca et al.	2009	Ischemic change	Traditional ECG reading	N/A	1. Commercial ECG system 2. ECG manually annotated by cardiologists	Signal Processing	N	Y	N	N	N	Decision Tree Classifier, bagging
Faganeli et al.	2010	ST changes	Traditional ECG reading	N/A	1. Commercial ECG system 2. ECG manually annotated by cardiologists	Algorithm-based	N	N	N	N	Y	Classification and Regression Tree Algorithm
Khoshnoud et al.	2010	MI	Traditional ECG reading	N/A	1. Commercial ECG system 2. ECG manually annotated by cardiologists	Neural Network	N	N	Y	N	N	Probabilistic Neural Networks
Bakhshipour et al.	2010	MI	Traditional ECG reading	N/A	1. Commercial ECG system 2. ECG manually annotated by cardiologists	Traditional Machine Learning	Y	Y	N	N	N	SVM, Fourier Transform
Sankari et al.	2011	MI	Traditional ECG reading	N/A	1. Commercial ECG system	Algorithm-based	N	N	N	N	Y	HeartSaver medical device algorithm
Forberg et al.	2012	STEMI	Traditional ECG reading	38/560 STEMI (7%)	1. Commercial ECG system 2. ECG manually annotated by cardiologists	Neural Network	N	N	Y	N	N	ANN
Dhawan et al.	2012	MI	Traditional ECG reading	112/201= 55.72%	1. Commercial ECG system 2. ECG manually annotated by cardiologists	Traditional Machine Learning	Y	Y	N	N	N	SVM, Genetic optimization algorithm
Park et al.	2012	Ischemic change	Traditional ECG reading	355/367=96.73%	1. Commercial ECG system 2. ECG manually annotated by cardiologists	Traditional Machine Learning	Y	Y	N	N	N	SVM, kernel density estimation (KDE)
Wilson et al.	2013	STEMI	Traditional ECG reading	7.50%	1. Commercial ECG system 2. ECG manually annotated by cardiologists	Algorithm-based	N	N	N	N	Y	Zoll E Series ECG monitor algorithm
Keshtkar et al.	2013	MI	Traditional ECG reading	N/A	1. Commercial ECG system 2. ECG manually annotated by cardiologists	Neural Network	N	Y	Y	N	N	Probabilistic neural network, discrete wavelength transform
Pelaez et al.	2013	MI	Traditional ECG reading	N/A	1. Commercial ECG system 2. ECG manually annotated by cardiologists	Neural Network	N	N	Y	N	N	ANN
Liu et al.	2014	MI	Traditional ECG reading	N/A	1. Commercial ECG system 2. ECG manually annotated by cardiologists	Algorithm-based	N	N	N	N	Y	PolyECG-S MI detection algorithm, J48 algorithm
Finlay et al.	2014	MI	Traditional ECG reading	N/A	1. Commercial ECG system 2. ECG manually annotated by cardiologists	Algorithm-based	N	N	N	N	Y	Body Suface Potential Maps Algorithm
Li et al.	2014	MI	Traditional ECG reading	N/A	1. Commercial ECG system 2. ECG manually annotated by cardiologists	Algorithm-based	N	N	N	N	Y	Fuzzy Associated Rule Mining Algorithm
Treskes et al.	2015	STEMI	Traditional ECG reading	N/A	1. Commercial ECG system 2. ECG manually annotated by cardiologists	Algorithm-based	N	N	N	N	Y	Leiden ECG Analysis and Decomposition Software algorithm
Kora et al.	2015	MI	Traditional ECG reading	N/A	1. Commercial ECG system 2. ECG manually annotated by cardiologists	Neural Network	N	Y	Y	N	Y	Bat algorithm, Sgolay FIR smoothing filter, Levenburg-Marquardt NN
Bansal et al.	2015	ST changes	Traditional ECG reading	N/A	1. Commercial ECG system 2. ECG manually annotated by cardiologists	Algorithm-based	N	N	N	N	Y	DanTest Bluetooth Sensory Device algorithhm

Rad et al.	2015	MI	Traditional ECG reading	N/A	1. Commercial ECG system 2. ECG manually annotated by cardiologists	Algorithm-based	N	N	N	N	Y	LabView Mobile Module Algorithm
Mohanta et al.	2015	MI	Traditional ECG reading	N/A	1. Commercial ECG system 2. ECG manually annotated by cardiologists	Neural Network	N	N	Y	N	N	Multilayer Feedforward Neural Network
Bhaskar	2015	MI	Traditional ECG reading	N/A	1. Commercial ECG system 2. ECG manually annotated by cardiologists	Neural Network	N	N	Y	N	N	ANN
Yu et al.	2016	MI	Traditional ECG reading	N/A	N/A	Algorithm-based	N	Y	N	N	Y	Discrete Wavelet Transform, MI beat detection algorithm
Garvey et al.	2016	STEMI	Traditional ECG reading	151/500 (30.2%)	1. Commercial ECG system 2. ECG manually annotated by cardiologists	Algorithm-based	N	N	N	N	Y	Diagnostic algirthm
Tseng et al.	2016	Ischemic change	Traditional ECG reading	N/A	1. Commercial ECG system 2. ECG manually annotated by cardiologists	Traditional Machine Learning	Y	N	N	N	N	SVM
Wu et al.	2016	MI	Traditional ECG reading	N/A	1. Commercial ECG system 2. ECG manually annotated by cardiologists	Algorithm-based	N	N	N	N	Y	Multi-Scale Deep Feature Learning
Murthy et al.	2016	MI	Traditional ECG reading	N/A	1. Commercial ECG system 2. ECG manually annotated by cardiologists	Algorithm-based	N	N	N	N	Y	Principal Component Analysis Algorithm
Firoozabadi et al.	2017	STEMI	Traditional ECG reading	N/A	1. Commercial ECG system 2. ECG manually annotated by cardiologists	Algorithm-based	N	N	N	N	Y	STEMI screening algorithm, Signal Quality Indicator (SQI) algorithm
Liu et al.	2017	MI	Traditional ECG reading	N/A	1. Commercial ECG system 2. ECG manually annotated by cardiologists	Neural Network	N	N	Y	N	N	Multi-lead CNN
Murthy	2017	MI	Traditional ECG reading	N/A	1. Commercial ECG system 2. ECG manually annotated by cardiologists	Neural Network	N	Y	Y	N	N	ANN, Independent-component analysis-wavelet packet decomposition
Kora	2017	MI	Traditional ECG reading	N/A	1. Commercial ECG system 2. ECG manually annotated by cardiologists	Neural Network, Algorithm-based	Y	N	Y	N	N	ANN, Hybrid Firefly and Swarm Optimization Algorithm
Kumar et al.	2017	MI	Traditional ECG reading	N/A	1. Commercial ECG system 2. ECG manually annotated by cardiologists	Traditional Machine Learning	Y	Y	N	N	N	SVM, RBF Kernel Function
Reasat et al.	2017	MI	Traditional ECG reading	N/A	1. Commercial ECG system 2. ECG manually annotated by cardiologists	Neural Network	N	N	Y	N	N	CNN
Dohare et al.	2017	MI	Traditional ECG reading	N/A	1. Commercial ECG system 2. ECG manually annotated by cardiologists	Traditional Machine Learning	Y	N	N	N	N	SVM
Sharma et al.	2017	MI	Traditional ECG reading	N/A	1. Commercial ECG system 2. ECG manually annotated by cardiologists	Traditional Machine Learning	Y	N	N	N	N	SVM
Sharma et al.	2018	MI	Traditional ECG reading	N/A	1. Commercial ECG system 2. ECG manually annotated by cardiologists	Algorithm-based	N	Y	N	N	Y	Bioorthogonal filter bank, covex optimization, KNN feature
Xiao et al.	2018	Ischemic change	Traditional ECG reading	N/A	1. Commercial ECG system 2. ECG manually annotated by cardiologists	Neural Network	N	N	Y	N	N	CNN
De La Fuentes-Cortes et al.	2018	MI	Traditional ECG reading	N/A	1. Commercial ECG system 2. ECG manually annotated by cardiologists	Algorithm-based	N	N	N	N	Y	Fuzzy logic algorithm
Wang et al.	2018	MI	Traditional ECG reading	N/A	1. Commercial ECG system 2. ECG manually annotated by cardiologists	Traditional Machine Learning	Y	Y	N	N	N	SVM, kernel function parameter (Gaussian)
Chen et al.	2018	MI	Traditional ECG reading	N/A	1. Commercial ECG system 2. ECG manually annotated by cardiologists	Neural Network	N	N	Y	N	N	MCL-CNN
Xue et al.	2018	MI	Traditional ECG reading	N/A	1. Commercial ECG system 2. ECG manually annotated by cardiologists	Hybrid Network	N	N	Y	Y	N	CNN-LSTM

Davydov et al.	2018	MI	Traditional ECG reading	N/A	1. Commercial ECG system 2. ECG manually annotated by cardiologists	Traditional Machine Learning	Y	N	N	N	N	SVM
Liu et al.	2018	MI	Traditional ECG reading	N/A	1. Commercial ECG system 2. ECG manually annotated by cardiologists	Neural Network	N	N	Y	N	N	Multi-Feature Branch CNN
Diker et al.	2018	MI	Traditional ECG reading	N/A	1. Commercial ECG system 2. ECG manually annotated by cardiologists	Traditional Machine Learning	Y	Y	N	N	N	SVM, Discrete Wavelet Transform
Lui et al.	2018	MI	Traditional ECG reading	N/A	1. Commercial ECG system 2. ECG manually annotated by cardiologists	Hybrid Network	N	N	N	Y	N	CNN-LSTM
Sopic et al.	2018	MI	Traditional ECG reading	Wearables	1. Commercial ECG system 2. ECG manually annotated by cardiologists	Algorithm-based	N	N	N	N	Y	Tarret Platform Algorithm
Sadhukhan et al.	2018	MI	Traditional ECG reading	N/A	1. Commercial ECG system 2. ECG manually annotated by cardiologists	Algorithm-based	N	N	N	N	Y	Harmonic Phase Algorithm
Avila	2019	STEMI	Traditional ECG reading	N/A	1. Commercial ECG system 2.Coronary angiogram readings by experienced physician	Algorithm-based	N	N	N	N	Y	Apple Watch 4 algorithm
Han et al.	2019	MI	Traditional ECG reading	N/A	1. Commercial ECG system 2. ECG manually annotated by cardiologists	Traditional Machine Learning	Y	Y	N	N	N	SVM, Radial Basis Function kernel
Strodthoff et al.	2019	MI	Traditional ECG reading	N/A	1. Commercial ECG system 2. ECG manually annotated by cardiologists	Neural Network	N	N	Y	N	N	CNN
Chowdhury et al.	2019	ST changes	Traditional ECG reading	N/A	1. Commercial ECG system 2. ECG manually annotated by cardiologists	Traditional Machine Learning	Y	Y	N	N	N	SVM, polynomial kernel function
Wu et al.	2019	NSTEMI	Troponin, Traditional ECG reading	N/A	1. Commercial ECG system 2. ECG manually annotated by cardiologists	Neural Network	N	N	Y	N	N	ANN
Liu et al.	2019	MI	Traditional ECG reading	N/A	1. Commercial ECG system 2. ECG manually annotated by cardiologists	Traditional Machine Learning	Y	Y	N	N	N	Decision Tree Classifier, bagging, discrete wavelength packet transformation (DWPT), multilinear principal component analysis (MPCA)
Liu et al.	2019	MI	Traditional ECG reading	N/A	1. Commercial ECG system 2. ECG manually annotated by cardiologists	Neural Network	N	N	Y	N	N	Multi-feature-branch convolutional bidirectional RNN (MFB-CBRNN)
Han et al.	2019	MI	Traditional ECG reading	N/A	1. Commercial ECG system 2. ECG manually annotated by cardiologists	Neural Network	N	N	Y	N	N	Multi-lead residual NN (ML-ResNet)
Muhlestein et al.	2019	STEMI	Traditional ECG reading	57/204=27.9%	1. Commercial ECG system 2. ECG manually annotated by cardiologists	Algorithm-based	N	N	N	N	Y	ST LEUIS smartphone algorithm
Wang et al.	2019	MI	Traditional ECG reading	N/A	1. Commercial ECG system 2. ECG manually annotated by cardiologists	Neural Network	N	N	Y	N	N	Multi-lead ensemble neural network (MENN)
Liu et al.	2019	MI	Traditional ECG reading	N/A	1. Commercial ECG system 2. ECG manually annotated by cardiologists	Traditional Machine Learning	Y	Y	N	N	N	KNN, Dual-Q Tunable Q-Factor Wavelet Transform
Hillinger et al.	2019	STEMI	Traditional ECG reading	MI:438/2486 STEMI:81/2486	1. Commercial ECG system 2. ECG manually annotated by cardiologists	Algorithm-based	N	N	N	N	Y	ECG Digitalized Algorithm
Sharma et al.	2019	MI	Traditional ECG reading	N/A	1. Commercial ECG system 2. ECG manually annotated by cardiologists	Algorithm-based	N	Y	N	N	Y	K-nearest neighbours, Mahalanobis and Euclidian Functions
She et al.	2019	MI	Traditional ECG reading	N/A	1. Commercial ECG system 2. ECG manually annotated by cardiologists	Neural Network	N	N	Y	N	N	Deep Neural Network
Sahu et al.	2019	MI	Traditional ECG reading	N/A	1. Commercial ECG system 2. ECG manually annotated by cardiologists	Traditional Machine Learning	Y	Y	N	N	N	SVM, RBF Kernel Function

Zhang et al.	2019	MI	Traditional ECG reading	N/A	1. Commercial ECG system 2. ECG manually annotated by cardiologists	Algorithm-based	N	N	N	N	Y	Tree Bagger Algorithm
Zhang et al.	2019	MI	Traditional ECG reading	N/A	1. Commercial ECG system 2. ECG manually annotated by cardiologists	Neural Network	N	N	Y	Y	N	CNN-LSTM
Darmawahyuni et al.	2019	MI	Traditional ECG reading	N/A	1. Commercial ECG system 2. ECG manually annotated by cardiologists	Neural Network	N	N	Y	N	N	LSTM Neural Network
Darmawahyuni et al.	2019	MI	Traditional ECG reading	N/A	1. Commercial ECG system 2. ECG manually annotated by cardiologists	Neural Network	N	N	Y	N	N	LSTM Neural Network
Zhang et al.	2019	MI	Traditional ECG reading	N/A	1. Commercial ECG system 2. ECG manually annotated by cardiologists	Neural Network	N	N	Y	N	N	Principal Component Analysis Network
Zhang et al.	2019	MI	Traditional ECG reading	N/A	1. Commercial ECG system 2. ECG manually annotated by cardiologists	Neural Network	N	N	Y	N	N	Bi-LSTM NN
Feng et al.	2019	MI	Traditional ECG reading	N/A	1. Commercial ECG system 2. ECG manually annotated by cardiologists	Hybrid Network	N	N	Y	Y	N	CNN-LSTM
Baloglu et al.	2019	MI	Traditional ECG reading	N/A	1. Commercial ECG system 2. ECG manually annotated by cardiologists	Neural Network	N	N	Y	N	N	CNN
Tripathy et al.	2019	MI	Traditional ECG reading	N/A	1. Commercial ECG system 2. ECG manually annotated by cardiologists	Traditional Machine Learning	Y	Y	N	N	N	SVM, Empirical Wavelet Transform
Spaccarotella et al.	2020	STEMI	Traditional ECG reading	STEMI= 54/100, NSTEMI=27/100	1. Commercial ECG system 2. ECG read by attending physician	Algorithm-based	N	N	N	N	Y	Apple Watch 4 algorithm
Hao et al.	2020	MI	Traditional ECG reading	N/A	1. Commercial ECG system 2. ECG manually annotated by cardiologists	Neural Network	N	Y	Y	N	N	Multi-Branch Fusion Framework (DenseNet), Feature Fusion
Cho et al.	2020	STEMI	Traditional ECG reading	16.1% (MI)	1. Commercial ECG system 2. ECG manually annotated by cardiologists	Hybrid Network	N	N	Y	Y	Y	Deep learning based algorithm, Variational autoencoder (VAE)
Brown et al.	2020	STEMI	Traditional ECG reading	N/A	1. Commercial ECG system 2. ECG manually annotated by cardiologists	Algorithm-based	N	N	N	N	Y	Apple Watch 5 algorithm
Makimoto et al.	2020	MI	Traditional ECG reading	N/A	1. Commercial ECG system 2. ECG manually annotated by cardiologists	Neural Network	N	N	Y	N	N	CNN
Zhao et al.	2020	STEMI	Traditional ECG reading	N/A	1. Commercial ECG system 2. ECG manually annotated by cardiologists	Algorithm-based	N	N	N	N	Y	Deep learning based algorithm
Fu et al.	2020	MI	Traditional ECG reading	N/A	1. Commercial ECG system 2. ECG manually annotated by cardiologists	Hybrid Network	N	N	N	Y	N	Multi-lead attention, CNN, bidirectional gated recurrent unit (BiGRU) framework
Kapfo et al.	2020	MI	Traditional ECG reading	N/A	1. Commercial ECG system 2. ECG manually annotated by cardiologists	Traditional Machine Learning	Y	N	N	N	N	SVM, KNN
Aufderheide et al.	2020	MI	Traditional ECG reading	N/A	1. Commercial ECG system 2. ECG manually annotated by cardiologists	Algorithm-based	N	N	N	N	Y	ST Algorithm
Ibrahim et al.	2020	MI	Traditional ECG reading	N/A	1. Commercial ECG system 2. ECG manually annotated by cardiologists	Neural Network	N	N	Y	N	N	CNN
Ogrezeanu et al.	2020	MI	Traditional ECG reading	N/A	1. Commercial ECG system 2. ECG manually annotated by cardiologists	Neural Network	N	N	Y	N	N	Conv1D Network
Muminov et al.	2020	MI	Traditional ECG reading	N/A	1. Commercial ECG system 2. ECG manually annotated by cardiologists	Neural Network	N	N	Y	N	N	CNN
Swain et al.	2020	MI	Traditional ECG reading	N/A	1. Commercial ECG system 2. ECG manually annotated by cardiologists	Algorithm-based	N	N	N	N	Y	Stockwell Transform Algorithm

Lin et al.	2020	MI	Traditional ECG reading	N/A	1. Commercial ECG system 2. ECG manually annotated by cardiologists	Algorithm-based	N	N	N	N	Y	KNN Algorithm
Wang et al.	2020	MI	Traditional ECG reading	N/A	1. Commercial ECG system 2. ECG manually annotated by cardiologists	Algorithm-based	N	N	N	N	Y	Random Forest Algorithm
Omar et al.	2020	MI	Traditional ECG reading	N/A	1. Commercial ECG system 2. ECG manually annotated by cardiologists	Hybrid Network	N	N	N	Y	N	CNN, Bi-LSTM
Zhang et al.	2020	MI	Traditional ECG reading	N/A	1. Commercial ECG system 2. ECG manually annotated by cardiologists	Neural Network	N	N	Y	N	N	MDD Net
Nasimov et al.	2020	MI	Traditional ECG reading	N/A	1. Commercial ECG system 2. ECG manually annotated by cardiologists	Neural Network	N	N	Y	N	N	CNN
Sridhar et al.	2020	MI	Traditional ECG reading	N/A	1. Commercial ECG system 2. ECG manually annotated by cardiologists	Traditional Machine Learning	Y	N	N	N	N	SVM
Hussein et al.	2021	Ischemic change	Traditional ECG reading	N/A	1. Commercial ECG system 2. ECG manually annotated by cardiologists	Traditional Machine Learning	Y	Y	N	N	N	SVM, Choi-Williams time-frequency distribution
Martin et al.	2021	MI	Traditional ECG reading	N/A	1. Commercial ECG system 2. ECG manually annotated by cardiologists	Neural Network	N	N	Y	N	N	Long-Short Term Memory (RNN)
Safdarian et al.	2021	MI	Traditional ECG reading	N/A	1. Commercial ECG system 2. ECG manually annotated by cardiologists	Traditional Machine Learning	Y	Y	N	N	N	SVM, Grasshopper optimization algorithm
Liu et al.	2021	MI	Traditional ECG reading	N/A	1. Commercial ECG system 2. ECG manually annotated by cardiologists	Neural Network	N	N	Y	N	N	EvoMBN (evolving multi-branch network)
Liu et al.	2021	MI	Traditional ECG reading	N/A	1. Commercial ECG system 2. ECG manually annotated by cardiologists	Neural Network	N	N	Y	N	N	ECG12Net
Tadesse et al.	2021	MI	Traditional ECG reading	N/A	1. Commercial ECG system 2. ECG manually annotated by cardiologists	Neural Network	N	Y	Y	N	N	MNasNet, LSTM method
He et al.	2021	MI	Traditional ECG reading	N/A	1. Commercial ECG system 2. ECG manually annotated by cardiologists	Neural Network	N	N	Y	N	N	Multi-branch lead attention neural network (MFB-LANN)
Chen et al.	2021	MI	Traditional ECG reading	N/A	1. Commercial ECG system 2. ECG manually annotated by cardiologists	Neural Network	N	N	N	N	N	ResNet
Cao et al.	2021	MI	Traditional ECG reading	N/A	1. Commercial ECG system 2. ECG manually annotated by cardiologists	Neural Network	N	N	Y	N	N	Multi-channel lightweight model (ML-Net)
Han et al.	2021	MI	Traditional ECG reading	2465/76829 = 3.21%	1. Commercial ECG system 2. ECG manually annotated by cardiologists	Neural Network	N	N	Y	N	N	CNN
Chang et al.	2021	MI	Traditional ECG reading	N/A	1. Commercial ECG system 2. ECG manually annotated by cardiologists	Neural Network	N	N	Y	N	N	LSTM
Jahmunah et al.	2021	MI	Traditional ECG reading	N/A	1. Commercial ECG system 2. ECG manually annotated by cardiologists	Neural Network	N	Y	Y	N	N	CNN, Gabor filter
Gibson et al.	2021	STEMI	Traditional ECG reading	50%	1. Commercial ECG system 2. ECG manually annotated by cardiologists	Neural Network	N	N	Y	N	N	1D CNN
Liu et al.	2021	MI	Traditional ECG reading	2.22% (74/3320)	1. Commercial ECG system 2. ECG manually annotated by cardiologists	Algorithm-based	N	N	N	N	Y	AI-S algorithm
Dai et al.	2021	MI	Traditional ECG reading	N/A	1. Commercial ECG system 2. ECG manually annotated by cardiologists	Neural Network	N	N	Y	N	N	ResNet
Faramand et al.	2021	STEMI	Traditional ECG reading	STEMI: 53/143	1. Commercial ECG system 2. ECG manually annotated by cardiologists	Algorithm-based	N	N	N	N	Y	ST Algorithm
Fatimah et al.	2021	MI	Traditional ECG reading	N/A	1. Commercial ECG system 2. ECG manually annotated by cardiologists	Algorithm-based	N	N	N	N	Y	KNN Algorithm

Ma et al.	2021	MI	Traditional ECG reading	N/A	1. Commercial ECG system 2. ECG manually annotated by cardiologists	Neural Network	N	N	Y	N	N	Convolutional Dendrite Net
Zhao et al.	2021	MI	Traditional ECG reading	N/A	1. Commercial ECG system 2. ECG manually annotated by cardiologists	Neural Network	N	N	N	Y	N	CNN-LSTM
Yadav et al.	2021	MI	Traditional ECG reading	N/A	1. Commercial ECG system 2. ECG manually annotated by cardiologists	Neural Network	N	N	Y	N	N	CNN
Dey et al.	2021	MI	Traditional ECG reading	N/A	1. Commercial ECG system 2. ECG manually annotated by cardiologists	Hybrid Network	N	N	N	Y	N	CNN, Bi-LSTM
Sinha et al.	2021	MI	Traditional ECG reading	N/A	1. Commercial ECG system 2. ECG manually annotated by cardiologists	Traditional Machine Learning	Y	N	N	N	N	SVM
Liu et al.	2021	MI	Traditional ECG reading	N/A	1. Commercial ECG system 2. ECG manually annotated by cardiologists	Neural Network	N	N	Y	N	N	Evolving Multi-Branch Network
Martin et al.	2021	MI	Traditional ECG reading	N/A	1. Commercial ECG system 2. ECG manually annotated by cardiologists	Neural Network	N	N	Y	N	N	LSTM Neural Network
Choudhary et al.	2021	MI	Traditional ECG reading	N/A	1. Commercial ECG system 2. ECG manually annotated by cardiologists	Neural Network	N	N	Y	N	N	1D CNN
Jikui et al.	2021	MI	Traditional ECG reading	N/A	1. Commercial ECG system 2. ECG manually annotated by cardiologists	Neural Network	N	N	Y	N	N	CNN
Biglar et al.	2021	MI	Traditional ECG reading	N/A	1. Commercial ECG system 2. ECG manually annotated by cardiologists	Neural Network	N	N	Y	N	N	CNN
Kim et al.	2022	MI	Cardiac troponin assays, traditional ECG interpretation	STEMI: 54/80 (67.5%)	1. Commercial ECG system 2.Coronary angiogram labelings by ED doctors, verified by interventional cardiologist	Neural Network	N	N	Y	N	N	CNN, part of deep learning system Quantitative ECG (QCG)
Sun et al.	2022	MI	Coronary angiography, Thrombolysis in Myocardial Infarction flow grade (TIMI)		1. Commercial ECG system 2.Coronary angiogram readings by experienced physician	Traditional Machine Learning	Y	Y	N	N	N	SVM, Radial Basis Function kernel, Linear kernel, Boosting/Bagging tree
Chumachenko et al.	2022	MI	Troponin, Traditional ECG reading	N/A	1. Commercial ECG system 2. ECG read by cardiologists	Traditional Machine Learning	Y	Y	N	N	N	Random Forest decision tree classifier
Wu et al.	2022	ST changes	Traditional ECG reading	N/A	1. Commercial ECG system 2. ECG read by cardiologists	Neural Network	N	N	Y	N	N	ECG Bidirectional Transformer Network
Chen et al.	2022	STEMI	Traditional ECG reading	10/275 patients STEMI	1. Commercial ECG system 2. ECG read by attending physician	Hybrid Network	N	N	Y	Y	N	CNN-LSTM
Uchiyama et al.	2022	MI	Traditional ECG reading	N/A	1. Commercial ECG system 2. ECG manually annotated by cardiologists	Neural Network	N	N	Y	N	N	CNN
Fang et al.	2022	MI	Traditional ECG reading	N/A	1. Commercial ECG system 2. ECG manually annotated by cardiologists	Neural Network	N	N	Y	N	N	Multi-VCG deep CNN
Kumar et al.	2022	MI	Traditional ECG reading	10/70 (MI)	1. Commercial ECG system 2. ECG manually annotated by cardiologists	Neural Network	N	N	Y	N	N	ANN

Zhao et al.	2022	MI	Traditional ECG reading	N/A	1. Commercial ECG system 2. ECG manually annotated by cardiologists	Traditional Machine Learning	Y	Y	N	N	N	SVM, SampEN (sample entropy)
Jahmunah et al.	2022	MI	Traditional ECG reading	N/A	1. Commercial ECG system 2. ECG manually annotated by cardiologists	Neural Network	N	N	Y	N	N	DenseNet
Gaspardone et al.	2022	STEMI	Traditional ECG reading	N/A	1. Commercial ECG system 2. ECG manually annotated by cardiologists	Algorithm-based	N	N	N	N	Y	Decision tree analysis algorithm
Cao et al.	2022	MI	Traditional ECG reading	N/A	1. Commercial ECG system 2. ECG manually annotated by cardiologists	Neural Network	N	Y	Y	N	N	SENnet, Gradient class activation mapping algorithm
Barua et al.	2022	MI	Traditional ECG reading	N/A	1. Commercial ECG system 2. ECG manually annotated by cardiologists	Algorithm-based	N	N	N	N	Y	Multilevel unbalanced pooling tree transformation (MUPTT)
Sraitih et al.	2022	MI	Traditional ECG reading	70%	1. Commercial ECG system 2. ECG manually annotated by cardiologists	Traditional Machine Learning	Y	Y	N	N	N	SVM, K-nearest neighbours (KNN), random forest (RF)
Gustafsson et al.	2022	MI	Traditional ECG reading	N/A	1. Commercial ECG system 2. ECG manually annotated by cardiologists	Neural Network	N	N	Y	N	N	ResNet
Choi et al.	2022	STEMI	Troponin, Traditional ECG reading	N/A	1. Commercial ECG system 2. ECG manually annotated by cardiologists	Neural Network	N	N	Y	N	N	ResNet
Hammad et al.	2022	MI	Traditional ECG reading	N/A	1. Commercial ECG system 2. ECG manually annotated by cardiologists	Neural Network	Y	N	Y	N	N	CNN, SVM
Jahmunah et al.	2022	MI	Traditional ECG reading	N/A	1. Commercial ECG system 2. ECG manually annotated by cardiologists	Neural Network	N	N	Y	N	N	DenseNet
Wu et al.	2022	STEMI	Traditional ECG reading	315/793=39.72%	1. Commercial ECG system 2. ECG manually annotated by cardiologists	Hybrid Network	N	N	N	Y	N	CNN-LSTM
Choi et al.	2022	MI	Traditional ECG reading	N/A	1. Commercial ECG system 2. ECG manually annotated by cardiologists	Neural Network	N	N	Y	N	N	CNN binary classifier
Saradhi et al.	2022	MI	Traditional ECG reading	N/A	1. Commercial ECG system 2. ECG manually annotated by cardiologists	Traditional Machine Learning	Y	N	N	N	N	Ensemble Learning
Pan et al.	2022	MI	Traditional ECG reading	N/A	1. Commercial ECG system 2. ECG manually annotated by cardiologists	Neural Network	N	N	Y	N	N	MCA-Net (2D CNN)
Kavak et al.	2022	STEMI	Traditional ECG reading	N/A	1. Commercial ECG system 2. ECG manually annotated by cardiologists	Neural Network	N	N	Y	N	N	2D-CNN
Saha	2022	MI	Traditional ECG reading	N/A	1. Commercial ECG system 2. ECG manually annotated by cardiologists	Algorithm-based	N	N	N	N	Y	KNN Algorithm
He et al.	2022	MI	Traditional ECG reading	N/A	1. Commercial ECG system 2. ECG manually annotated by cardiologists	Neural Network	N	N	Y	N	N	DenseNet
Rajakumar et al.	2022	MI	Traditional ECG reading	N/A	1. Commercial ECG system 2. ECG manually annotated by cardiologists	Neural Network	N	N	Y	N	N	Probabilistic Neural Networks
Ma et al.	2022	MI	Traditional ECG reading	N/A	1. Commercial ECG system 2. ECG manually annotated by cardiologists	Neural Network	N	N	Y	N	N	CDDNet
Xu et al.	2022	MI	Traditional ECG reading	N/A	1. Commercial ECG system 2. ECG manually annotated by cardiologists	Neural Network	N	N	Y	N	N	Bi-LSTM NN
Mirza et al.	2022	MI	Traditional ECG reading	N/A	1. Commercial ECG system 2. ECG manually annotated by cardiologists	Neural Network	N	N	Y	N	N	1D CNN
Han et al.	2022	MI	Traditional ECG reading	N/A	1. Commercial ECG system 2. ECG manually annotated by cardiologists	Neural Network	N	N	Y	N	N	DenseNet

Elmannai et al.	2022	MI	Traditional ECG reading	N/A	1. Commercial ECG system 2. ECG manually annotated by cardiologists	Neural Network	N	N	Y	N	N	CNN
Li et al.	2022	MI	Traditional ECG reading	N/A	1. Commercial ECG system 2. ECG manually annotated by cardiologists	Neural Network	N	N	Y	N	N	Generative Adversarial Network
Rai et al.	2022	MI	Traditional ECG reading	N/A	1. Commercial ECG system 2. ECG manually annotated by cardiologists	Hybrid Network	N	N	Y	Y	N	CNN-LSTM
Tseng et al.	2023	MI	Cardiac troponin assays, traditional ECG interpretation	LAD: 208/384, LCX: 44/384, RCA: 132/384	1. Commercial ECG system 2.Coronary angiogram readings by interventional cardiologists	Neural Network	N	Y	Y	N	N	CNN, Fourier Transform, Continuous (Morlet) Wavelet Transform, Tierala's Criteria
Park et al.	2023	ACS	Traditional ECG reading	N/A	1. Commercial ECG system 2. ECG read by cardiologists	Neural Network	N	Y	Y	N	N	ResNet-based CNN, QcG Analyzer, Adam optimizer
Olsson de Capretz et al.	2023	MI	Traditional ECG reading	11.10%	1. Commercial ECG system 2. ECG manually annotated by cardiologists	Neural Network	N	Y	Y	N	N	ANN, Glasgow Program, Logistic regression
Al-Zaiti et al.	2023	MI	Troponin, Traditional ECG reading	13.70%	1. Commercial ECG system 2. ECG manually annotated by cardiologists	Algorithm-based	N	N	N	N	Y	Random forest model, Tree SHAP algorithms, OMI scoring
Xiao et al.	2023	MI	Traditional ECG reading	N/A	1. Commercial ECG system 2. ECG manually annotated by cardiologists	Hybrid Network	N	N	N	Y	N	1D CNN, xResNet
Qin et al.	2023	NSTEMI	Traditional ECG reading	N/A	1. Commercial ECG system 2. ECG manually annotated by cardiologists	Algorithm-based	N	N	N	N	Y	RF, SelectKBest, XGBoost Algorithms
Xiong et al.	2023	MI	Traditional ECG reading	N/A	1. Commercial ECG system 2. ECG manually annotated by cardiologists	Neural Network	N	N	Y	N	N	DenseNet, Gated Recursive Unit
Rajput et al.	2023	MI	Traditional ECG reading	N/A	1. Commercial ECG system 2. ECG manually annotated by cardiologists	Algorithm-based	N	N	N	N	Y	Logistic Regression
Firoz et al.	2023	MI	Traditional ECG reading	N/A	1. Commercial ECG system 2. ECG manually annotated by cardiologists	Hybrid Network	N	N	Y	Y	N	CNN-LSTM
Han et al.	2023	MI	Traditional ECG reading	N/A	1. Commercial ECG system 2. ECG manually annotated by cardiologists	Neural Network	N	N	Y	N	N	ResNet
Bhaskarpandit et al.	2023	MI	Traditional ECG reading	N/A	1. Commercial ECG system 2. ECG manually annotated by cardiologists	Neural Network	N	N	Y	N	N	Eigendomain-based deep representation learning
Moghadam et al.	2023	MI	Traditional ECG reading	N/A	1. Commercial ECG system 2. ECG manually annotated by cardiologists	Algorithm-based	N	N	N	N	Y	Tree Bagger Algorithm

Abbreviation: N/A: Not available, CAD: Coronary artery disease, LBBB: Left bundle branch block, AF: atrial fibrillation, MI: myocardial infraction, STEMI: ST elevation MI, NSTMI, Non-ST elevation MI, , ACS: Acute Coronary Syndrome, ECG: electrocardiograph, LVH: Left Ventricular Hypertrophy

Supplementary Table 5. Detailed Diagnostic Test Accuracy of AI algorithms of primary studies.

Author	Year	AI Category	Sensitivity	Specificity	PPV	NPV	Accuracy	AUC	Diagnostic Performance	Other major findings
Baxt	1991	ANN	97.2	96.2	N/A	N/A	N/A	N/A		
Bosnjak et al.	1995	CNN	89.6	N/A	84.3	N/A			N/A	
Silipo et al.	1995		77	N/A	86	N/A	N/A	N/A		
Kaiser et al.	1996		76.2	98.1	98.6	N/A			N/A	59 rules for anterior MI, 53 rules for inferior MI
Polak et al.	1997		72	66	N/A	N/A	67		Accuracy = 67%	
Heden et al.	1997		47.1	95.2	N/A	N/A			N/A	
Maglaveras et al.	1997	ANN	88.6	78.4	N/A	N/A	N/A			
Bezerianos et al.	2000	CNN	73.6	N/A	68.3	N/A				
Garcia et al.	2000	ANN	85	N/A	86	N/A			N/A	
Papaloukas et al.	2001	SVM	90	N/A	89	N/A			N/A	
Baxt et al.	2002	ANN	88.1	86.2	N/A	N/A			N/A	
Langley et al.	2003		82.3	90.2	N/A	N/A	88.4			
Haraldsson et al.	2004		63.3	85	N/A	N/A		83.4	ROC = 83.4	
Heden et al.	2004		81	95	N/A	N/A			N/A	
Andreao et al.	2004		83	N/A	85	N/A	N/A			
Yang et al.	2005	ANN	65	100	N/A	N/A			N/A	
Mohebbi et al.	2007		94.5	N/A	85.0	N/A	N/A			
Mohebbi et al.	2007	SVM	92.1	N/A	90.3	N/A	N/A			
Olsson et al.	2008	ANN	27	98	N/A	N/A			N/A	
Afsar et al.	2008	DenseNet	90.8	N/A	89.2	N/A			N/A	
Zizzo et al.	2008		85	N/A	93	N/A			N/A	
Jayachandran et al.	2009	ANN	N/A	N/A	N/A	N/A	95		Overall accuracy= 95%	

Dranca et al.	2009	ANN	68.3	N/A	74.9	N/A			N/A	
Faganeli et al.	2010	ANN	93.8	92.6	N/A	N/A		97.4	AUC=97.4	
Khoshnoud et al.	2010	ANN	96.7	89.2	N/A	N/A	90.75			
Bakhshipour et al.	2010		98.4	99.5	N/A	N/A	98.2			
Sankari et al.	2011		95	100	N/A	N/A			N/A	
Forberg et al.	2012	ANN	90	68	18	99		93	AUROC =0.93	
Dhawan et al.	2012		86.8	91.1	86.7	91.3			N/A	
Park et al.	2012		94.1	92.3	N/A	N/A			N/A	
Wilson et al.	2013		95.6	94.6	59.5	99.6	93.8		Accuracy =93.8%	
Keshtkar et al.	2013	LSTM	93	86	N/A	N/A	89.5		Accuracy =89.5%	
Pelaez et al.	2013	ANN	80.8	88.5	N/A	N/A	N/A	N/A		
Liu et al.	2014	SSVM	87.7	56.7	N/A	N/A	80.7		Accuracy =80.7%	
Finlay et al.	2014		84.4	86.7	N/A	N/A	N/A			
Li et al.	2014		83.4	80.7	N/A	N/A	81.4	N/A		
Treskes et al.	2015		70.2	89.1	N/A	N/A			N/A	
Bansal et al.	2015		94	72.6	N/A	N/A	99.3		Accuracy =99.32%	
Kora et al.	2015		93.3	92.2	N/A	N/A	98.9		Accuracy =98.9%	
Rad et al.	2015		98	93.3	N/A	N/A			N/A	
Mohanta et al.	2015	ANN	91	N/A	94	N/A	N/A	N/A		
Bhaskar	2015	ANN	83.9	70.9	N/A	N/A	82.1			
Garvey et al.	2016		79	97	22.9	99.8		88	ROC=0.88	
Yu et al.	2016	SVM	95.2	90.5	N/A	N/A	91.8		Overall accuracy = 91.80	Using MI beats and episodes to develop algorithm effective
Tseng et al.	2016	ResNet	94.8	99.5	99.9	N/A			N/A	
Wu et al.	2016		99.6	99.8	N/A	N/A	N/A			

Murthy et al.	2016		96.2	N/A	93.8	N/A	90.51	N/A		
Firoozabadi et al.	2017	ANN	87	96	N/A	N/A			N/A	
Liu et al.	2017		95.4	97.4	N/A	N/A	96		Accuracy=96%	
Murthy	2017	ANN	97.2	N/A	99.6	N/A	96.5			
Kora	2017	ANN	100.0	98.7	N/A	N/A	99.3			
Kumar et al.	2017		99.1	97.4	N/A	N/A	98.3	N/A		
Reasat et al.	2017	CNN	85.3	84.1	N/A	N/A	84.5	N/A		
Dohare et al.	2017		96.7	100	N/A	N/A	98.3			
Sharma et al.	2017	SVM	99.4	98.3	98.4	N/A	98.8	99.94		
Xiao et al.	2018	SVM	84.4	84.9	N/A	N/A				
Sharma et al.	2018	ResNet	99.8	99.1	99.8	N/A		1	AUC=1, F1= 0.9976	
De La Fuentes-Cortes et al.	2018		96.4	96.9	N/A	N/A		96.5	AUC=0.965	
Wang et al.	2018		99.7	N/A	N/A	N/A	99.8	N/A		
Chen et al.	2018	CNN	93.7	97.3	N/A	N/A	96.2	95.5		
Xue et al.	2018	CNN-LSTM	91.7	81.5	N/A	N/A	89			
Davydov et al.	2018		87	84.8	N/A	N/A	86.0			
Liu et al.	2018	CNN	100.0	100.0	N/A	N/A	100.0			
Diker et al.	2018		87.0	88.6	N/A	N/A	87.8	N/A		
Lui et al.	2018		92.4	97.7	97.2	N/A	N/A	N/A	F1=94.6%	
Sopic et al.	2018		N/A	N/A	N/A	N/A	90	N/A		
Sadhukhan et al.	2018		96.6	92.7	N/A	N/A	95.6			
Avila	2019	SVM	N/A	N/A	N/A	N/A	100		Overall accuracy =100%	Apple Watch 4 matched STEMI diagnosis
Chowdhury et al.	2019		92	89	N/A	N/A	87.1		Accuracy=87.1%	
Wu et al.	2019		90.9	93.3	76.9	97.7	92.9	98.4	AUROC =98.4%, Accuracy =92.86%	

Muhlestein et al.	2019		89	84	70	95			N/A	
Liu et al.	2019		96.7	83.7	N/A	N/A	83.6		Accuracy =83.6%	
Liu et al.	2019	LSTM	93.4	80.9	96.1	N/A	91.3		Accuracy = 91.32%	
Han et al.	2019		94.9	97.4	N/A	N/A	95.5		Accuracy =95.5%	
Wang et al.	2019	ResNet	98.4	97.5	N/A	N/A		97.9	AUC=97.9%	
Liu et al.	2019	CNN	81.7	94.1	N/A	N/A	86.3		Accuracy=0.863	
Han et al.	2019	CNN, SVM	99.6		99.7	N/A				
Strodthoff et al.	2019	DenseNet	93.3	89.7	93.6	N/A			N/A	
Hillinger et al.	2019		35	99	54	N/A	35			
Sharma et al.	2019		98.6	99.4	99.4	N/A	99	99		
She et al.	2019		92.5	96.3	N/A	N/A	95.2			
Sahu et al.	2019		98.9	99.9	98.9	N/A	99.0			
Zhang et al.	2019		100.0	99.5	99.9	N/A	N/A			
Zhang et al.	2019	CNN-LSTM	99.9	100.0	100.0	N/A	100.0			
Darmawahyuni et al.	2019		98.5	98.0	95.7	N/A	N/A	N/A	F1=96.3	
Darmawahyuni et al.	2019		91	91	N/A	N/A	90	N/A		
Zhang et al.	2019		93.91	89.2	N/A	N/A	93.17	N/A		
Zhang et al.	2019		95.58	90.48	N/A	N/A	94.77	N/A		
Feng et al.	2019	CNN-LSTM	98.2	86.5	N/A	N/A	95.4	N/A	F1=96.8	
Baloglu et al.	2019	CNN	99	N/A	N/A	N/A	99.8	N/A		
Tripathy et al.	2019		99.9	99.6	N/A	N/A	99.7			
Spaccarotella et al.	2020		93	95	N/A	N/A			N/A	Apple Watch 4 multichannel detection agreement with standard ECG
Hao et al.	2020		96.4	95.9	N/A	N/A	94.7		Accuracy=94.7, F1= 93.8%	Assess ECG based on printed/screenshot ECG not device limited
Cho et al.	2020		89.2	92	36.9	99.4		97.4	AUROC=0.97	
Brown et al.	2020		N/A	N/A	N/A	N/A	100		Accuracy = 100%	

Zhao et al.	2020		96.75	99.2	87.05	N/A	99.01	99.54	Accuracy=99.01%, AUROC=0.995	
Makimoto et al.	2020		86	76	79	85	81		F1=0.82, accuracy=0.81	
Fu et al.	2020	CNN	97.1	93.3	N/A	N/A	96.5		Accuracy=96.5%	
Kapfo et al.	2020		99.9	99.9	N/A	N/A	99.9			
Aufderheide et al.	2020		90	56	40	94	N/A	N/A		
Ibrahim et al.	2020	CNN	88.1	93.2	N/A	N/A	89.1			
Ogrezeanu et al.	2020	CNN	89.1	82.7	81.7	N/A	85.71		F1=0.852	
Muminov et al.	2020	CNN	N/A	98.2	N/A	N/A	98.4			
Swain et al.	2020		100.0	99.3	N/A	N/A	99.9			
Lin et al.	2020		99.8	99.8	N/A	N/A	99.6			
Wang et al.	2020		83.9	97.7	N/A	N/A	85.8	N/A	F1=83.9	
Omar et al.	2020	CNN-LSTM	100	98.2	N/A	N/A	98.5	N/A	F1=99.1	
Zhang et al.	2020		89.2	97.8	92.2	N/A	96.9			
Nasimov et al.	2020	CNN	95	80	93	N/A	91.1	N/A	F1=94	
Sridhar et al.	2020		98.9	93.8	N/A	N/A	97.96	N/A		
Hussein et al.	2021		99.5	98.4	N/A	N/A	99.09		Accuracy =99.1%	
Safdarian et al.	2021	SVM	100	100	N/A	N/A	100		Accuracy=100%	
Liu et al.	2021		89.7	94.6	50.6	N/A		97.6	AUC=0.976	
Tadesse et al.	2021	CNN	67.6	83.3	89.8	N/A	72.5		Accuracy=72.5%	
Han et al.	2021		71.8	86.6	8.1	99.5		99	AUROC=0.88	
Chang et al.	2021	CNN	87	N/A	95.2	N/A	98.7	99.7	AUC=0.997, accuracy=0.987	
Jahmunah et al.	2021		100.0	100.0	99.6	N/A	100.0		Accuracy=99.95%	
Gibson et al.	2021	SVM	96.3	96.8	N/A	N/A	96.5		Accuracy=96.5%	
Liu et al.	2021	CNN	82.2	99.8	N/A	N/A		99.6	AUC=0.996	
Bigler et al.	2021	SVM	93	92	N/A	N/A		92.4	AUC=0.924	

He et al.	2021		99.6	99.9	99.6	N/A	99.6		Accuracy=99.6%	
Cao et al.	2021	CNN	94.3	97.72	N/A	N/A	96.7	96.7	Accuracy=96.7%, AUC=96.7%	
Martin et al.	2021	CNN-LSTM	75.85	83.02	N/A	N/A	77.1		Accuracy=77.1%	
Chen et al.	2021	CNN	82.4	95	82.7	N/A				
Dai et al.	2021	ResNet	99.0	99.9	98.4	N/A	99.6			
Faramand et al.	2021		62.9	95.6	47.6	97.6	93.7			
Fatimah et al.	2021		100.0	100.0	N/A	N/A	100.0			
Ma et al.	2021		98.17	99.22	N/A	N/A	99.0	N/A		
Zhao et al.	2021	CNN-LSTM	90.9	94.9	N/A	N/A	94			
Yadav et al.	2021	CNN	99.9	99.65	N/A	N/A	99.8			
Dey et al.	2021	CNN-LSTM	99.2	99.66	99.9	N/A	99.3	N/A	F1=99.5	
Sinha et al.	2021	SVM	98.2	99.3	N/A	N/A	989.0			
Liu et al.	2021		98.5	90.0	98.0	N/A	97.1	N/A	F1=98.3	
Martin et al.	2021		91.9	80.8	N/A	N/A	89.6	N/A		
Choudhary et al.	2021		93.4	84.6	N/A	N/A	92.3			
Jikui et al.	2021	CNN	99.77	99.4	N/A	N/A	99.5	N/A		
Biglar et al.	2021	CNN	93	92	N/A	N/A	N/A	92.4		
Kim et al.	2022	ANN	98.1	76.9	89.8	95.2		94.7	AUC of QCG score = 94.7	
Sun et al.	2022		90.5	92.9	N/A	N/A	91.11		Overall accuracy = 91.11	Boosting and Bagging algorithm with SVM better than Linear and RBF kernels
Wu et al.	2022		93.1	96	60	N/A				
Chen et al.	2022		94.1	99.4	N/A	N/A		99.7	AUROC = 0.997	Real-time AI assisted remote detection of STEMI possible
Kumar et al.	2022		100	100	N/A	N/A	100		Accuracy = 100%	
Gaspardone et al.	2022		96	93	93	96	88		(for LAD) Overall accuracy=88%	
Cao et al.	2022		99.9	100.0	N/A	N/A	100.0		Accuracy=99.98%	
Barua et al.	2022		99.9	N/A	100.0	N/A	99.9		Accuracy=99.94%	
Choi et al.	2022		97.4	99.2	20.2	99.9		99.8	AUROC=0.998	

Wu et al.	2022		97	97	99	99	98		Accuracy=98%	
Choi et al.	2022		85.4	82.4	83.7	84.2		91.9	AUC=0.919	
Fang et al.	2022	CNN	97.3	90.8	N/A	N/A	95.7	98.6	AUROC= 98.61%, Accuracy=95.65%	3D ECG with DNN for MI identification
Zhao et al.	2022	SVM	90.3	90.5	N/A	N/A	90.3	90.4	AUC=0.904, accuracy= 0.903	
Jahmunah et al.	2022		91.7	99.5	72.6	N/A	87.6		Accuracy=87.60%	
Sraitih et al.	2022		70	N/A	73	N/A	74		Accuracy=74%	
Jahmunah et al.	2022	CNN-LSTM	90.4	98.7	N/A	N/A	98.9		Overall accuracy=98.9%	Inferior MI location
Chumachenko et al.	2022	CNN	N/A	N/A	N/A	N/A	97.8		Overall accuracy = 97.774%	
Uchiyama et al.	2022	ResNet, CNN	99.9	99.7	N/A	N/A	99.8		Accuracy=99.82%	Detect MI without preprocessing images
Gustafsson et al.	2022	ANN	N/A	N/A	N/A	N/A		99.1	AUROC=0.991	
Hammad et al.	2022		98.8	N/A	98.9	N/A	99.6		Accuracy=99.62%	
Saradhi et al.	2022		92	N/A	94	N/A	93.7		F1=0.93	
Pan et al.	2022	CNN	88.6	96.6	N/A	N/A	93.7	N/A		
Kavak et al.	2022	CNN	96.2	89.4	N/A	N/A	96.3	96.2	F1=0.926	
Saha	2022		90	91	N/A	N/A	N/A	N/A	F1=0.9	
He et al.	2022	DenseNet	95.9	99.2	95.8	N/A	96.1		F1= 95.85	
Rajakumar et al.	2022		98.3	N/A	N/A	N/A	98.5	N/A		
Ma et al.	2022		97.6	99.1	N/A	N/A	98.5	N/A		
Xu et al.	2022		98.5	99.7	98.4	99.7	99.6	N/A		
Mirza et al.	2022		99.9	100.0	99.91	N/A	100.0	N/A	F1=99.91	
Han et al.	2022	DenseNet	90.5	97.3	N/A	N/A	91.9	N/A	F1=90.43	
Elmannai et al.	2022	CNN	99	N/A	99	N/A	99.7		F1=99	
Li et al.	2022		99.3	98.7	99.1	N/A	99.1	N/A	F1=99.24	
Rai et al.	2022	CNN-LSTM	98.8	98.8	99.3	N/A	99.7		F1=99.03	

Tseng et al.	2023	CNN	LAD: 81.7, LCX: 94.2, RCA: 64.5	LAD: 71.8, LCX: 94.20, RCA: 69.7	LAD: 74.3, LCX: 45.3, RCA: 69.7	N/A	69.9		Overall accuracy = 69.92	Preprocessing ECG signals with CWT was superior than STFT
Park et al.	2023		75.8	75.6	87.2	58.6	75.7		F1= 0.81, Accuracy = 75.7%	
Olsson de Capretz et al.	2023	CNN	99.5	98.2	69.8	99.9			N/A	
Al-Zaiti et al.	2023	SVM	86	98	54	99		87.3	AUROC =0.873	
Xiao et al.	2023	ResNet, CNN	69.9	92.9	76.8	N/A	87.1	91.5	AUROC=0.915, Accuracy=87.1%	
Qin et al.	2023		98	94	N/A	N/A	95			
Xiong et al.	2023	DenseNet	99.94	99.96	N/A	N/A	100.0			
Rajput et al.	2023		86	80	N/A	N/A	80.2		F1=0.83	
Firoz et al.	2023	CNN-LSTM	100	88	N/A	N/A	97.7		F1=0.94	
Han et al.	2023	ResNet	96.2	97.6	N/A	96.55	N/A		F1=97.65	
Bhaskarpandit et al.	2023		99.0	99.49	N/A	N/A	99.0	N/A		
Moghadam et al.	2023		99.7	96.3	96.1	N/A	96.5		F1= 97.88	

Abbreviation: **CNN** – Convolutional Neural Network; **SVM** – Support Vector Machine; **LSTM** – Long Short-Term Memory; **SSVM** – Structured Support Vector Machine; **ResNet** – Residual Neural Network; **ANN** – Artificial Neural Network; **DenseNet** – Densely Connected Convolutional Network; **AUC** – Area Under the Curve; **AUROC** – Area Under the Receiver Operating Characteristic Curve; **F1** – F1 Score (harmonic mean of precision and recall); **n/a** – Not Applicable; **LAD** – Left Anterior Descending artery; **LCX** – Left Circumflex artery;**RCA** – Right Coronary Artery.

Suppleentary Information 1. Search strategy

Q1. Artificial Intelligence in ECG Monitoring to eliminate Artifacts

Q2. Artificial Intelligence in ECG Monitoring for Myocardial Infarction

Q3. Artificial Intelligence in ECG Monitoring for Atrial Fibrillation

---

Database: Ovid MEDLINE(R) ALL <1946 to May 10, 2023>
Search Strategy:
1 exp algorithms/ or algorithm$.mp. or (artificial$ adj3 intelligence$).tw,kf. (638480)
2 ((bat or evolutionary or gravitational$ afj2 search$ or (imperialist$ adj2 competitive$) or firefly or (swarm$ adj2 intelligence$) or memetic$ or heuristic$) adj3 algorithm$).tw,kf. (4622)
3 ((simulated$ adj2 annealing$) or (Tabu$ adj2 search$)).tw. (3832)
4 (metaheuristic$ or (computer$ adj3 heuristic$) or meta-heuristic$ or hyper?heuristic$).tw,kf. (1437)
5 ((automated$ adj3 reasoning$) or (ambient$ adj3 intelligence$) or ((multicriteria$ or (multiple$ adj2 criteria$) or multi-criteria$) adj3 decision$) or ((multicriteria$ or (multiple$ adj2 criteria$) or (multiple$ adj3 objective$) or multiobjective$ or multi-objective$ or multi-criteria$) adj3 optimization$)).tw. (4714)
6 ((machine$ adj2 learning$) or (deep$ adj2 learning$) or (computational$ adj3 Intelligence$) or (predictive$ adj3 analytic*)).tw,kf. (125718)
7 ((support$ adj2 vector$) or (convolution$ adj3 neural$ adj3 net$1) or (neural$ adj2 network*)).tw,kf. (117184)
8 (bioinformatics$ or bayesian$).tw,kf. (144615)
9 ((computer$ adj3 vision$) or (visuali$ adj5 (cluster$ or heat?map$))).tw,kf. (9538)
10 exp Wearable Electronic Devices/ or *Smartphone/ or (mobile$ phone$ or smartphon$ or smartwatch$ or (Apple$ adj2 Watch$) or (smart$ adj2 (device$ or phone$ or watch$ or wristband$ or wrist-band$ or wrist$)) or chatbot$ or chat-bot$ or ChatGPT or Chat-GPT).tw,kf. (57503)
11 (device$ adj2 (haptic$ or wearable$ or vibro?tactile$ or wristband$ or wrist-band$)).tw,kf. (8709)
12 exp Telemetry/ or (eHealth or e-Health or mHealth or m-health or telehealth$ or tele-health$ or telemonitor$ or tele-monitor$).tw,kf. (48314)
13 or/1-12 (931855)
14 exp *Electrocardiography/ (77589)
15 (electrocardiograph$ or ekg or ecg or electrocardiogram$ or cardioscope$).ti. or (electrocardiograph$ or ekg or ecg or electrocardiogram$ or cardioscope$).ab. /freq=2 (79683)
16 or/14-15 (116333)

17 **artifacts/** or (artefact$ or artifact$).tw,kf. (86518)
18 13 and 16 and 17 (976)
19 limit 18 to "all adult (19 plus years)" (214)
20 limit 18 to "all child (0 to 18 years)" (32)
21 18 not (20 not (19 and 20)) (961)
22 (pediatr$ or paediatr$ or child$ or adolescent$ or infan$ or newborn$ or neonat$).ti. (1573734)
23 21 not 22 (960)
24 23 not (exp Animals/ not (Human/ and exp Animals/)) (906)
25 24 not (animal$1 or mice or rat or rats or cat$1 or cattle$1 or dog$1 or goat$1 or horse$1 or rabbit$1 or sheep$1 or swine$1 or pig$1 or piglet* or canine$1 or feline$1 or porcine$ or calf or primate* or rodent$ or hamster$ or lamb$1 or monkey$1 or murine or veterinar*).ti. (902)
26 exp "Sensitivity and Specificity"/ or false negative reactions/ or false positive reactions/ or diagnostic errors/ or (sensitiv$ or specificity or distinguish$ or differentiat$ or enhancement or identif$ or detect$ or diagnos$ or accur$).tw,kf. or (predictive adj4 value$).tw,kf. or (false adj (positive$ or negative$)).tw,kf. or (receiver operat$ adj (characteristic$ or curve or analysis)).tw,kf. or (ROC or SROC).tw,kf. [diagnostic filter_MEDLINE] (10706920)
27 25 and 26 **(741)**

28 exp **Myocardial Infarction/** or (infarct$ adj2 myocardial$).tw. or AMI.ti. or AMI.ab. /freq=2 (282699)
29 Myocardial Ischemia/ or ((ischemia$ or ischaemia$) adj2 myocardial$).tw,kf. (68174)
30 or/28-29 (332430)

31 13 and 16 and 30 (1164)
32 limit 31 to "all adult (19 plus years)" (480)
33 limit 31 to "all child (0 to 18 years)" (29)
34 31 not (33 not (32 and 33)) (1162)
35 (pediatr$ or paediatr$ or child$ or adolescent$ or infan$ or newborn$ or neonat$).ti. (1573734)
36 34 not 35 (1161)
37 36 not (exp Animals/ not (Human/ and exp Animals/)) (1119)
38 37 not (animal$1 or mice or rat or rats or cat$1 or cattle$1 or dog$1 or goat$1 or horse$1 or rabbit$1 or sheep$1 or swine$1 or pig$1 or piglet* or canine$1 or feline$1 or porcine$ or calf or primate* or rodent$ or hamster$ or lamb$1 or monkey$1 or murine or veterinar*).ti. (1116)
39 exp "Sensitivity and Specificity"/ or false negative reactions/ or false positive reactions/ or diagnostic errors/ or (sensitiv$ or specificity or distinguish$ or differentiat$ or enhancement or identif$ or detect$ or diagnos$ or accur$).tw,kf. or (predictive adj4 value$).tw,kf. or (false adj (positive$ or negative$)).tw,kf. or (receiver operat$ adj (characteristic$ or curve or analysis)).tw,kf. or (ROC or SROC).tw,kf. [diagnostic filter_MEDLINE] (10706920)
40 38 and 39 **(943)**

41 **atrial fibrillation/** or atrial flutter/ (73422)
42 ((atrial$ or auricular$) adj3 (fibrillation$ or flutter$)).tw,kf. (96182)
43 ((AVAF or AF) and (atrial or flutter)).tw,kf. or (atrial or flutter).ab. /freq=2 (86740)
44 or/41-43 (135959)
45 13 and 16 and 44 (1505)
46 limit 45 to "all adult (19 plus years)" (579)
47 limit 45 to "all child (0 to 18 years)" (49)
48 45 not (47 not (46 and 47)) (1497)
49 (pediatr$ or paediatr$ or child$ or adolescent$ or infan$ or newborn$ or neonat$).ti. (1573734)
50 48 not 49 (1496)
51 50 not (exp Animals/ not (Human/ and exp Animals/)) (1472)
52 51 not (animal$1 or mice or rat or rats or cat$1 or cattle$1 or dog$1 or goat$1 or horse$1 or rabbit$1 or sheep$1 or swine$1 or pig$1 or piglet* or canine$1 or feline$1 or porcine$ or calf or primate* or rodent$ or hamster$ or lamb$1 or monkey$1 or murine or veterinar*).ti. (1470)
53 exp "Sensitivity and Specificity"/ or false negative reactions/ or false positive reactions/ or diagnostic errors/ or (sensitiv$ or specificity or distinguish$ or differentiat$ or enhancement or identif$ or detect$ or diagnos$ or accur$).tw,kf. or (predictive adj4 value$).tw,kf. or (false adj (positive$ or negative$)).tw,kf. or (receiver operat$ adj (characteristic$ or curve or analysis)).tw,kf. or (ROC or SROC).tw,kf. [diagnostic filter_MEDLINE] (10706920)
54 52 and 53 **(1295)**

55 **ST Elevation Myocardial Infarction**/ (7412)
56 (STEMI or (((ST-segment$ or ST segment$) adj4 elevat$) or (ST adj3 elevat$) or (non-ST adj3 elevat$) or (ST-elevation$ or non-ST-elevation$))).tw,kf. (37193)
57 or/55-56 (37972)
58 13 and 16 and 57 (339)
59 13 and 16 and 57 (339)
60 limit 59 to "all adult (19 plus years)" (167)
61 limit 59 to "all child (0 to 18 years)" (7)
62 59 not (61 not (60 and 61)) (338)
63 (pediatr$ or paediatr$ or child$ or adolescent$ or infan$ or newborn$ or neonat$).ti. (1573734)
64 62 not 63 (338)
65 64 not (exp Animals/ not (Human/ and exp Animals/)) (335)
66 65 not (animal$1 or mice or rat or rats or cat$1 or cattle$1 or dog$1 or goat$1 or horse$1 or rabbit$1 or sheep$1 or swine$1 or pig$1 or piglet* or canine$1 or feline$1 or porcine$ or calf or primate* or rodent$ or hamster$ or lamb$1 or monkey$1 or murine or veterinar*).ti. (334)
67 exp "Sensitivity and Specificity"/ or false negative reactions/ or false positive reactions/ or diagnostic errors/ or (sensitiv$ or specificity or distinguish$ or differentiat$ or enhancement or identif$ or detect$ or diagnos$ or accur$).tw,kf. or (predictive adj4 value$).tw,kf. or (false adj (positive$ or negative$)).tw,kf. or (receiver operat$ adj (characteristic$ or curve or analysis)).tw,kf. or (ROC or SROC).tw,kf. [diagnostic filter_MEDLINE] (10706920)
68 66 and 67 (307)

69 27 or 40 or 54 or 68 **(2863)**

Database: Embase Classic+Embase <1947 to 2023 May 10>
Search Strategy:
1 exp *algorithm/ or exp *machine learning/ or exp *artificial intelligence/ or algorithm$.tw,kw. or (artificial$ adj3 intelligence$).tw,kw. (618220)
2 ((bat or evolutionary or gravitational$ afj2 search$ or (imperialist$ adj2 competitive$) or firefly or (swarm$ adj2 intelligence$) or memetic$ or heuristic$) adj3 algorithm$).tw,kw. (4783)
3 ((simulated$ adj2 annealing$) or (Tabu$ adj2 search$)).tw. (3865)
4 (metaheuristic$ or (computer$ adj3 heuristic$) or meta-heuristic$ or hyper?heuristic$).tw,kw. (1419)
5 ((automated$ adj3 reasoning$) or (ambient$ adj3 intelligence$) or ((multicriteria$ or (multiple$ adj2 criteria$) or multi-criteria$) adj3 decision$) or ((multicriteria$ or (multiple$ adj2 criteria$) or (multiple$ adj3 objective$) or multiobjective$ or multi-objective$ or multi-criteria$) adj3 optimization$)).tw. (5421)
6 ((machine$ adj2 learning$) or (deep$ adj2 learning$) or (computational$ adj3 Intelligence$) or (predictive$ adj3 analytic*)).tw,kw. (139052)
7 ((support$ adj2 vector$) or (convolution$ adj3 neural$ adj3 net$1) or (neural$ adj2 network*)).tw,kw. (137258)
8 (bioinformatics$ or bayesian$).tw,kw. (174829)
9 ((computer$ adj3 vision$) or (visuali$ adj5 (cluster$ or heat?map$))).tw,kw. (9854)
10 exp *wearable computer/ or *exp mobile phone/ or (mobile$ phone$ or smartphon$ or smartwatch$ or (Apple$ adj2 Watch$) or (smart$ adj2 (device$ or phone$ or watch$ or wristband$ or wrist-band$ or wrist$)) or chatbot$ or chat-bot$ or ChatGPT or Chat-GPT).tw,kw. (51488)
11 (device$ adj2 (haptic$ or wearable$ or vibro?tactile$ or wristband$ or wrist-band$)).tw,kw. (8801)
12 exp *telehealth/ or exp telemedicine/ or (eHealth or e-Health or mHealth or m-health or telehealth$ or tele-health$ or telemonitor$ or tele-monitor$).tw,kw. (96311)
13 or/1-12 (997920)
14 *electrocardiography/ or ambulatory electrocardiography/ or electrocardiography monitoring/ (69426)
15 (electrocardiograph$ or ekg or ecg or electrocardiogram$ or cardioscope$).ti. or (electrocardiograph$ or ekg or ecg or electrocardiogram$ or cardioscope$).ab. /freq=2 (121178)
16 or/14-15 (155092)

17 **exp artifact/** or artifact reduction/ or (artefact$ or artifact$).tw,kw. (110551)
18 13 and 16 and 17 (971)
19 limit 18 to (adult or aged) (275)
20 limit 18 to (embryo or infant or child) (19)
21 18 not (20 not (19 and 20)) (960)
22 (pediatr$ or paediatr$ or child$ or adolescent$ or infan$ or newborn$ or neonat$).ti. (2065586)
23 21 not 22 (950)
24 23 not ((exp animal/ or nonhuman/) not exp human/) (904)
25 24 not (animal$1 or mice or rat or rats or cat$1 or cattle$1 or dog$1 or goat$1 or horse$1 or rabbit$1 or sheep$1 or swine$1 or pig$1 or piglet* or canine$1 or feline$1 or porcine$ or calf or primate* or rodent$ or hamster$ or lamb$1 or monkey$1 or murine or veterinar*).ti. (898)
26 "sensitivity and specificity"/ or laboratory diagnosis/ or prediction/ or "prediction and forecasting"/ or receiver operating characteristic/ or roc curve/ or diagnostic accuracy/ or diagnostic value/ or reliability/ or (sensitiv$ or specificity or distinguish$ or differentiat$ or enhancement or identif$ or detect$ or diagnos$ or accur$).tw,kw. or (predictive adj4 value$).tw,kw. or (false adj (positive$ or negative$)).tw,kw. or (receiver operat$ adj (characteristic$ or curve or analysis)).tw,kw. or (ROC or SROC).tw,kw. [diagnostic filter_EMBASE] (14657461)
27 25 and 26 **(753)**

28 exp ***heart infarction/** or (infarct$ adj2 myocardial$).tw. or AMI.ti. or AMI.ab. /freq=2 (383499)
29 exp *heart muscle ischemia/ or ((ischemia$ or ischaemia$) adj2 myocardial$).tw,kw. (80680)
30 or/28-29 (441825)
31 13 and 16 and 30 (1139)
32 limit 31 to (adult or aged) (535)
33 limit 31 to (embryo or infant or child) (4)

34 31 not (33 not (32 and 33)) (1139)
35 (pediatr$ or paediatr$ or child$ or adolescent$ or infan$ or newborn$ or neonat$).ti. (2065586)
36 34 not 35 (1139)
37 36 not ((exp animal/ or nonhuman/) not exp human/) (1121)
38 37 not (animal$1 or mice or rat or rats or cat$1 or cattle$1 or dog$1 or goat$1 or horse$1 or rabbit$1 or sheep$1 or swine$1 or pig$1 or piglet* or canine$1 or feline$1 or porcine$ or calf or primate* or rodent$ or hamster$ or lamb$1 or monkey$1 or murine or veterinar*).ti. (1117)
39 "sensitivity and specificity"/ or laboratory diagnosis/ or prediction/ or "prediction and forecasting"/ or receiver operating characteristic/ or roc curve/ or diagnostic accuracy/ or diagnostic value/ or reliability/ or (sensitiv$ or specificity or distinguish$ or differentiat$ or enhancement or identif$ or detect$ or diagnos$ or accur$).tw,kw. or (predictive adj4 value$).tw,kw. or (false adj (positive$ or negative$)).tw,kw. or (receiver operat$ adj (characteristic$ or curve or analysis)).tw,kw. or (ROC or SROC).tw,kw. [diagnostic filter_EMBASE] (14657461)
40 38 and 39 **(1009)**

41 exp ***atrial fibrillation/** or *heart atrium flutter/ or *heart atrium fibrillation/ (100951)
42 ((atrial$ or auricular$) adj3 (fibrillation$ or flutter$)).tw,kw. (172326)
43 ((AVAF or AF) and (atrial or flutter)).tw,kw. or (atrial or flutter).ab. /freq=2 (155381)
44 or/41-43 (223225)
45 13 and 16 and 44 (1877)
46 limit 45 to (adult or aged) (1012)
47 limit 45 to (embryo or infant or child) (24)
48 45 not (47 not (46 and 47)) (1868)
49 (pediatr$ or paediatr$ or child$ or adolescent$ or infan$ or newborn$ or neonat$).ti. (2065586)
50 48 not 49 (1863)
51 50 not ((exp animal/ or nonhuman/) not exp human/) (1840)
52 51 not (animal$1 or mice or rat or rats or cat$1 or cattle$1 or dog$1 or goat$1 or horse$1 or rabbit$1 or sheep$1 or swine$1 or pig$1 or piglet* or canine$1 or feline$1 or porcine$ or calf or primate* or rodent$ or hamster$ or lamb$1 or monkey$1 or murine or veterinar*).ti. (1832)
53 "sensitivity and specificity"/ or laboratory diagnosis/ or prediction/ or "prediction and forecasting"/ or receiver operating characteristic/ or roc curve/ or diagnostic accuracy/ or diagnostic value/ or reliability/ or (sensitiv$ or specificity or distinguish$ or differentiat$ or enhancement or identif$ or detect$ or diagnos$ or accur$).tw,kw. or (predictive adj4 value$).tw,kw. or (false adj (positive$ or negative$)).tw,kw. or (receiver operat$ adj (characteristic$ or curve or analysis)).tw,kw. or (ROC or SROC).tw,kw. [diagnostic filter_EMBASE] (14657461)
54 52 and 53 **(1668)**

55 **ST segment elevation myocardial infarction**/ (51497)
56 (STEMI or (((ST-segment$ or ST segment$) adj4 elevat$) or (ST adj3 elevat$) or (non-ST adj3 elevat$) or (ST-elevation$ or non-ST-elevation$))).tw,kw. (72314)
57 or/55-56 (84972)
58 13 and 16 and 57 (556)
59 13 and 16 and 57 (556)
60 limit 59 to (adult or aged) (285)
61 limit 59 to (embryo or infant or child) (1)
62 59 not (61 not (60 and 61)) (556)
63 (pediatr$ or paediatr$ or child$ or adolescent$ or infan$ or newborn$ or neonat$).ti. (2065586)
64 62 not 63 (556)
65 64 not ((exp animal/ or nonhuman/) not exp human/) (554)
66 65 not (animal$1 or mice or rat or rats or cat$1 or cattle$1 or dog$1 or goat$1 or horse$1 or rabbit$1 or sheep$1 or swine$1 or pig$1 or piglet* or canine$1 or feline$1 or porcine$ or calf or primate* or rodent$ or hamster$ or lamb$1 or monkey$1 or murine or veterinar*).ti. (551)
67 "sensitivity and specificity"/ or laboratory diagnosis/ or prediction/ or "prediction and forecasting"/ or receiver operating characteristic/ or roc curve/ or diagnostic accuracy/ or diagnostic value/ or reliability/ or (sensitiv$ or specificity or distinguish$ or differentiat$ or enhancement or identif$ or detect$ or diagnos$ or accur$).tw,kw. or (predictive adj4 value$).tw,kw. or (false adj (positive$ or negative$)).tw,kw. or (receiver operat$ adj (characteristic$ or curve or analysis)).tw,kw. or (ROC or SROC).tw,kw. [diagnostic filter_EMBASE] (14657461)
68 66 and 67 **(506)**

69 27 or 40 or 54 or 68 **(3334)**

Scopus 1,777 documents found in **Scopus**

Year 1958-2023 **1,777** documents found

(TITLE ((detect* OR predict* OR identif* OR explor* OR classif* OR locali* OR diagnos* OR record* OR determin* OR distinguish* OR differentiat* OR accur* OR recogni* OR analys*)) AND TITLE (ischem* OR ischaemia* OR myocard* OR st-elevat* OR st-segment* OR st-t-segment* OR mi-detect* OR stemi OR non-st-elevation* OR heart-infarction* OR atrial-fibrillation* OR atrial-flutter* OR atrium-flutter* OR atrium-fibrillation*) AND TITLE-ABS-KEY ((electrocardiograph* OR ekg OR ecg OR ecgs OR mi-beat* OR ischaemic-beat* OR ischemic-beat* OR electrocardiogram* OR cardioscope*)) AND TITLE (algorithm* OR artificial-intelligence* OR ambient-intelligence* OR computational OR machine-learning* OR deep-learning* OR deep-knowledge* OR deep-cnn* OR neural-network* OR thinking-network* OR siamese-network* OR deep OR deepmi* OR hybrid-network* OR attention-network* OR multi-instance-learning* OR multi-task-learning* OR ensemble-learning* OR deterministic-learning* OR multi-branch-network* OR deep-lstm-network* OR multilayer-network* OR transformer-network* OR temporal-convolutional-network* OR semi-supervised-learning* OR densenet* OR optimal-features* OR automatic OR automated OR smartwatch* OR smart-watch* OR apple-watch OR handheld* OR hand-held* OR smartphone* OR smart-phone* OR smart-device* OR smart-wristband* OR smart-wrist-band* OR smartwristband* OR smart-wrist* OR interpretable-ensemble-tree* OR telemedicine* OR cnn OR network OR application OR biomedical OR signal* OR bioinformatics* OR bayesian* OR chatbot* OR chat-bot* OR chatgpt* OR chat-gpt* OR haptic* OR wearable* OR w-ecg* OR vibro-tactile* OR vibrotactile* OR computer* OR meta-heuristic* OR metaheuristic* OR resnet* OR srtnet* OR wavelet-transform* OR karhunen-loeve-transform* OR bidirectional-transform* OR transformer-based* OR real-time-frequency* OR correcting-window* OR remote-health-monitoring-system* OR difference-vector* OR support-vector-machine* OR classifier-ensemble* OR ensemble-neural-classifier* OR automatic-detection* OR auto-detection* OR heartsaver* OR mobile* OR wristband* OR movement-activity-recognition* OR pre-mobile* OR apps)) AND (LIMIT-TO (LANGUAGE , "English"))

1,341 records found in Compendex for 1884-2023:

Year 1976-2023 n=1341 records found (2013-2022_926 1976-2021_415

(((((((detect* OR predict* OR identif* OR explor* OR classif* OR locali* OR diagnos* OR record* OR determin* OR distinguish* OR differentiat* OR accur* OR recogni* OR analys*)) WN TI) AND (((ischem* OR ischaemia* OR myocard* OR st-elevat* OR st-segment* OR ST-T-segment* OR MI-detect* OR stemi OR non-st-elevation* OR heart-infarction* OR atrial-fibrillation* OR atrial-flutter* OR atrium-flutter* OR atrium-fibrillation*)) WN ALL)) AND (((electrocardiograph* OR ekg OR ecg OR ecgs OR MI-beat* OR ischaemic-beat* OR ischemic-beat* OR electrocardiogram* OR cardioscope*)) WN ALL)) AND (((algorithm* OR artificial-intelligence* OR ambient-intelligence* OR computational OR machine-learning*

OR deep-learning* OR deep-knowledge* OR deep-cnn* OR neural-network* OR thinking-network* OR siamese-network* OR deep OR deepmi* OR hybrid-network* OR attention-network* OR multi-instance-learning* OR multi-task-learning* OR ensemble-learning* OR deterministic-learning* OR Multi-branch-Network* OR Deep-LSTM-network* OR multilayer-network* OR Transformer-network* OR Temporal-Convolutional-Network* OR semi-supervised-learning* OR densenet* OR optimal-features* OR automatic OR automated OR smartwatch* OR smart-watch* OR apple-watch OR handheld* OR hand-held* OR smartphone* OR smart-phone* OR smart-device* OR smart-wristband* OR smart-wrist-band* OR smartwristband* OR smart-wrist* OR interpretable-ensemble-tree* OR telemedicine* OR cnn OR network OR application OR biomedical OR signal* OR bioinformatics* OR bayesian* OR chatbot* OR chat-bot* OR chatgpt* OR chat-gpt* OR haptic* OR wearable* OR W-ECG* OR vibro-tactile* OR vibrotactile* OR computer* OR meta-heuristic* OR metaheuristic* OR resnet* OR srtnet* OR wavelet-transform* OR Karhunen-Loeve-transform* OR Bidirectional-Transform* OR transformer-based* OR real-time-frequency* OR correcting-window* OR Remote-health-monitoring-system* OR difference-vector* OR support-vector-machine* OR classifier-ensemble* or ensemble-neural-classifier* OR automatic-detection* OR auto-detection* OR HeartSaver* OR Mobile* OR wristband* OR movement-activity-recognition* OR Pre-Mobile* OR Apps)) WN TI)) NOT ((animal* or mice or rat or rats or cat or cats or cattle* or dog or dogs or goat or goats or horse horses or rabbit* or sheep* or swine* or pig or pigs or piglet* or canine* or feline* or porcine* or calf or primate* or rodent* or hamster* or lamb or lambs or monkey* or murine or veterinar* or pediatr* or paediatr* or child* or adolescent* or infan* or newborn* or neonat*) WN TI)) + {english} WN LA

Database: CINAHL Complete n=286 results

*Query* *Results*

S6 S5 NOT ((MH "Animals+") NOT ((MH "Human") AND (MH "Animals+"))) Limiters - English Language View Results (286)

S5 (TI (algorithm* OR artificial-intelligence* OR ambient-intelligence* OR computational OR machine-learning* OR deep-learning* OR deep-knowledge* OR deep-cnn* OR neural-network* OR thinking-network* OR siamese-network* OR deep OR deepmi* OR hybrid-network* OR attention-network* OR multi-instance-learning* OR multi-task-learning* OR ensemble-learning* OR deterministic-learning* OR Multi-branch-Network* OR Deep-LSTM-network* OR multilayer-network* OR Transformer-network* OR Temporal-Convolutional-Network* OR semi-supervised-learning* OR densenet* OR optimal-features* OR automatic OR automated OR smartwatch* OR smart-watch* OR apple-watch OR handheld* OR hand-held* OR smartphone* OR smart-phone* OR smart-device* OR smart-wristband* OR smart-wrist-band* OR smartwristband* OR smart-wrist* OR interpretable-ensemble-tree* OR telemedicine* OR cnn OR network OR application OR biomedical OR signal* OR bioinformatics* OR bayesian* OR chatbot* OR chat-bot* OR chatgpt* OR chat-gpt* OR haptic* OR wearable* OR W-ECG* OR vibro-tactile* OR vibrotactile* OR computer* OR meta-heuristic* OR metaheuristic* OR resnet* OR srtnet* OR wavelet-transform* OR Karhunen-Loeve-transform* OR Bidirectional-Transform* OR transformer-based* OR real-time-frequency* OR correcting-window* OR Remote-health-monitoring-

system* OR difference-vector* OR support-vector-machine* OR classifier-ensemble* or ensemble-neural-classifier* OR automatic-detection* OR auto-detection* OR HeartSaver* OR Mobile* OR wristband* OR movement-activity-recognition* OR Pre-Mobile* OR Apps)) Limiters - English Language View Results (286)

S4 TI (algorithm* OR artificial-intelligence* OR ambient-intelligence* OR computational OR machine-learning* OR deep-learning* OR deep-knowledge* OR deep-cnn* OR neural-network* OR thinking-network* OR siamese-network* OR deep OR deepmi* OR hybrid-network* OR attention-network* OR multi-instance-learning* OR multi-task-learning* OR ensemble-learning* OR deterministic-learning* OR Multi-branch-Network* OR Deep-LSTM-network* OR multilayer-network* OR Transformer-network* OR Temporal-Convolutional-Network* OR semi-supervised-learning* OR densenet* OR optimal-features* OR automatic OR automated OR smartwatch* OR smart-watch* OR apple-watch OR handheld* OR hand-held* OR smartphone* OR smart-phone* OR smart-device* OR smart-wristband* OR smart-wrist-band* OR smartwristband* OR smart-wrist* OR interpretable-ensemble-tree* OR telemedicine* OR cnn OR network OR application OR biomedical OR signal* OR bioinformatics* OR bayesian* OR chatbot* OR chat-bot* OR chatgpt* OR chat-gpt* OR haptic* OR wearable* OR W-ECG* OR vibro-tactile* OR vibrotactile* OR computer* OR meta-heuristic* OR metaheuristic* OR resnet* OR srtnet* OR wavelet-transform* OR Karhunen-Loeve-transform* OR Bidirectional-Transform* OR transformer-based* OR real-time-frequency* OR correcting-window* OR Remote-health-monitoring-system* OR difference-vector* OR support-vector-machine* OR classifier-ensemble* or ensemble-neural-classifier* OR automatic-detection* OR auto-detection* OR HeartSaver* OR Mobile* OR wristband* OR movement-activity-recognition* OR Pre-Mobile* OR Apps) View Results (232,102)

S3 TI (electrocardiograph* OR ekg OR ecg OR ecgs OR MI-beat* OR ischaemic-beat* OR ischemic-beat* OR electrocardiogram* OR cardioscope*) OR AB (electrocardiograph* OR ekg OR ecg OR ecgs OR MI-beat* OR ischaemic-beat* OR ischemic-beat* OR electrocardiogram* OR cardioscope*) View Results (31,746)

S2 TI (ischem* OR ischaemia* OR myocard* OR st-elevat* OR st-segment* OR ST-T-segment* OR MI-detect* OR stemi OR non-st-elevation* OR heart-infarction* OR atrial-fibrillation* OR atrial-flutter* OR atrium-flutter* OR atrium-fibrillation*) View Results (108,371)

S1 TI (detect* OR predict* OR identif* OR explor* OR classif* OR locali* OR diagnos* OR record* OR determin* OR distinguish* OR differentiat* OR accur* OR recogni* OR analys*) View Results (921,989)

Supplementary Information 2. Citations of all included studies

1 Zhang G, Si Y, Yang W, Wang D. A robust multilevel dwt densely network for cardiovascular disease classification. *Sensors* 2020; **20**: 4777
2 Tseng L-M, Chuang C-Y, Chua S-K, Tseng VS. Identification of Coronary Culprit Lesion in ST Elevation Myocardial Infarction by Using Deep Learning. *IEEE Journal of Translational Engineering in Health and Medicine* 2022; **11**: 70-9
3 Kim D, Hwang JE, Cho Y, et al. A retrospective clinical evaluation of an artificial intelligence screening method for early detection of STEMI in the emergency department. *Journal of Korean medical science* 2022; **37**
4 Sun Q, Liang C, Chen T, et al. Early detection of myocardial ischemia in 12-lead ECG using deterministic learning and ensemble learning. *Computer Methods and Programs in Biomedicine* 2022; **226**: 107124
5 Kaiser W, Faber TS, Findeis M. Automatic learning of rules: a practical example of using artificial intelligence to improve computer-based detection of myocardial infarction and left ventricular hypertrophy in the 12-lead ECG. *Journal of electrocardiology* 1996; **29**: 17-20
6 Yu S-N, Tsai P-C. Myocardial ischemic beat and episode detection based on morphology and correcting window method. 3465-8
7 Afsar FA, Arif M, Yang J. Detection of ST segment deviation episodes in ECG using KLT with an ensemble neural classifier. *Physiological measurement* 2008; **29**: 747
8 Avila CO. Novel use of Apple Watch 4 to obtain 3-lead electrocardiogram and detect cardiac ischemia. *The Permanente Journal* 2019; **23**
9 Spaccarotella CAM, Polimeni A, Migliarino S, et al. Multichannel electrocardiograms obtained by a smartwatch for the diagnosis of ST-segment changes. *JAMA cardiology* 2020; **5**: 1176-80
10 Bezerianos A, Vladutu L, Papadimitriou S. Hierarchical state space partitioning with a network self-organising map for the recognition of ST-T segment changes. *Medical and Biological Engineering and Computing* 2000; **38**: 406-15
11 Chumachenko D, Butkevych M, Lode D, Frohme M, Schmailzl KJG, Nechyporenko A. Machine learning methods in predicting patients with suspected myocardial infarction based on short-time HRV data. *Sensors* 2022; **22**: 7033
12 Wu S, Cao Q, Chen Q, et al. Using Multi-Task Learning-Based Framework to Detect ST-Segment and J-Point Deviation From Holter. *Frontiers in Physiology* 2022; **13**: 912739
13 Sankari Z, Adeli H. HeartSaver: A mobile cardiac monitoring system for auto-detection of atrial fibrillation, myocardial infarction, and atrio-ventricular block. *Computers in biology and medicine* 2011; **41**: 211-20
14 Jayachandran ES, Joseph K P, Acharya U R. Analysis of myocardial infarction using discrete wavelet transform. *Journal of medical systems* 2010; **34**: 985-92
15 Park J, Yoon Y, Cho Y, Kim J. Feasibility of Artificial Intelligence–Based Electrocardiography Analysis for the Prediction of Obstructive Coronary Artery Disease in Patients With Stable Angina: Validation Study. *JMIR cardio* 2023; **7**: e44791
16 Chen K-W, Wang Y-C, Liu M-H, et al. Artificial intelligence-assisted remote detection of ST-elevation myocardial infarction using a mini-12-lead electrocardiogram device in prehospital ambulance care. *Frontiers in Cardiovascular Medicine* 2022; **9**: 1001982
17 García J, Sornmo L, Olmos S, Laguna P. Automatic detection of ST-T complex changes on the ECG using filtered RMS difference series: application to ambulatory ischemia monitoring. *IEEE Transactions on Biomedical Engineering* 2000; **47**: 1195-201
18 Haraldsson H, Edenbrandt L, Ohlsson M. Detecting acute myocardial infarction in the 12-lead ECG using Hermite expansions and neural networks. *Artificial intelligence in medicine* 2004; **32**: 127-36

19 Han C, Shi L. Automated interpretable detection of myocardial infarction fusing energy entropy and morphological features. *Computer methods and programs in biomedicine* 2019; **175**: 9-23
20 Strodthoff N, Strodthoff C. Detecting and interpreting myocardial infarction using fully convolutional neural networks. *Physiological measurement* 2019; **40**: 015001
21 Yang T-F, Devine B, Macfarlane PW. Use of artificial neural networks within deterministic logic for the computer ECG diagnosis of inferior myocardial infarction. *Journal of Electrocardiology* 1994; **27**: 188-93
22 Sharma M, San Tan R, Acharya UR. A novel automated diagnostic system for classification of myocardial infarction ECG signals using an optimal biorthogonal filter bank. *Computers in biology and medicine* 2018; **102**: 341-56
23 Hao P, Gao X, Li Z, Zhang J, Wu F, Bai C. Multi-branch fusion network for Myocardial infarction screening from 12-lead ECG images. *Computer methods and programs in biomedicine* 2020; **184**: 105286
24 Uchiyama R, Okada Y, Kakizaki R, Tomioka S. End-to-End Convolutional Neural Network Model to Detect and Localize Myocardial Infarction Using 12-Lead ECG Images without Preprocessing. *Bioengineering* 2022; **9**: 430
25 Chowdhury MEH, Alzoubi K, Khandakar A, et al. Wearable real-time heart attack detection and warning system to reduce road accidents. *Sensors* 2019; **19**: 2780
26 Polak MJ, Zhou SH, Rautaharju PM, Armstrong WW, Chaitman BR. Using automated analysis of the resting twelve-lead ECG to identify patients at risk of developing transient myocardial ischaemia-an application of an adaptive logic network. *Physiological Measurement* 1997; **18**: 317
27 Olsson SE, Ohlsson M, Öhlin H, Edenbrandt L. Neural networks–a diagnostic tool in acute myocardial infarction with concomitant left bundle branch block. *Clinical physiology and functional imaging* 2002; **22**: 295-9
28 Fang R, Lu C-C, Chuang C-T, Chang W-H. A visually interpretable detection method combines 3-D ECG with a multi-VGG neural network for myocardial infarction identification. *Computer Methods and Programs in Biomedicine* 2022; **219**: 106762
29 Kumar R, Aggarwal Y, Kumar Nigam V. Heart rate dynamics in the prediction of coronary artery disease and myocardial infarction using artificial neural network and support vector machine. *Journal of Applied Biomedicine* 2022; **20**: 70-9
30 Faganeli J, Jager F. Automatic classification of transient ischaemic and transient non-ischaemic heart-rate related ST segment deviation episodes in ambulatory ECG records. *Physiological measurement* 2010; **31**: 323
31 Cho Y, Kwon J-m, Kim K-H, et al. Artificial intelligence algorithm for detecting myocardial infarction using six-lead electrocardiography. *Scientific reports* 2020; **10**: 20495
32 Wilson RE, Kado HS, Percy RF, et al. An algorithm for identification of ST-elevation myocardial infarction patients by emergency medicine services. *The American Journal of Emergency Medicine* 2013; **31**: 1098-102
33 Treskes RW, Ter Haar CC, Man S, et al. Performance of ST and ventricular gradient difference vectors in electrocardiographic detection of acute myocardial ischemia. *Journal of electrocardiology* 2015; **48**: 498-504
34 Wu C-C, Hsu W-D, Islam MM, et al. An artificial intelligence approach to early predict non-ST-elevation myocardial infarction patients with chest pain. *Computer methods and programs in biomedicine* 2019; **173**: 109-17
35 Dranca L, Goni A, Illarramendi A. Real-time detection of transient cardiac ischemic episodes from ECG signals. *Physiological measurement* 2009; **30**: 983

36 Hedén B, Edenbrandt L, Haisty Jr WK, Pahlm O. Artificial neural networks for the electrocardiographic diagnosis of healed myocardial infarction. *The American journal of cardiology* 1994; **74**: 5-8
37 Forberg JL, Khoshnood A, Green M, et al. An artificial neural network to safely reduce the number of ambulance ECGs transmitted for physician assessment in a system with prehospital detection of ST elevation myocardial infarction. *Scandinavian Journal of Trauma, Resuscitation and Emergency Medicine* 2012; **20**: 1-9
38 Papaloukas C, Fotiadis DI, Likas A, Michalis LK. An ischemia detection method based on artificial neural networks. *Artificial intelligence in medicine* 2002; **24**: 167-78
39 Zhao X, Zhang J, Gong Y, et al. Reliable detection of myocardial ischemia using machine learning based on temporal-spatial characteristics of electrocardiogram and vectorcardiogram. *Frontiers in physiology* 2022; **13**: 854191
40 Stark K, Czermak T, Massberg S, Orban M. Watch out for ST-elevation myocardial infarction: a case report of ST-elevation in single-lead electrocardiogram tracing of a smartwatch. *European Heart Journal: Case Reports* 2020; **4**: 1
41 Makimoto H, Höckmann M, Lin T, et al. Performance of a convolutional neural network derived from an ECG database in recognizing myocardial infarction. *Scientific reports* 2020; **10**: 8445
42 Liu J, Zhang C, Zhu Y, Ristaniemi T, Parviainen T, Cong F. Automated detection and localization system of myocardial infarction in single-beat ECG using Dual-Q TQWT and wavelet packet tensor decomposition. *Computer methods and programs in biomedicine* 2020; **184**: 105120
43 Liu W, Wang F, Huang Q, Chang S, Wang H, He J. MFB-CBRNN: A hybrid network for MI detection using 12-lead ECGs. *IEEE journal of biomedical and health informatics* 2019; **24**: 503-14
44 Hussein AF, Hashim SJ, Rokhani FZ, Wan Adnan WA. An automated high-accuracy detection scheme for myocardial ischemia based on multi-lead long-interval ECG and Choi-Williams time-frequency analysis incorporating a multi-class SVM classifier. *Sensors* 2021; **21**: 2311
45 Jahmunah V, Ng EYK, Tan R-S, Oh SL, Acharya UR. Uncertainty quantification in DenseNet model using myocardial infarction ECG signals. *Computer Methods and Programs in Biomedicine* 2023; **229**: 107308
46 Han C, Shi L. ML–ResNet: A novel network to detect and locate myocardial infarction using 12 leads ECG. *Computer methods and programs in biomedicine* 2020; **185**: 105138
47 Keshtkar A, Seyedarabi H, Sheikhzadeh P, Rasta SH. Discriminant analysis between myocardial infarction patients and healthy subjects using Wavelet Transformed signal averaged electrocardiogram and probabilistic neural network. *Journal of Medical Signals & Sensors* 2013; **3**: 225-30
48 Zizzo C, Hassani A, Turner D. Automatic detection and imaging of ischemic changes during electrocardiogram monitoring. *IEEE Transactions on Biomedical Engineering* 2008; **55**: 1243-7
49 Martin H, Morar U, Izquierdo W, Cabrerizo M, Cabrera A, Adjouadi M. Real-time frequency-independent single-Lead and single-beat myocardial infarction detection. *Artificial intelligence in medicine* 2021; **121**: 102179
50 Gaspardone C, Romagnolo D, Fasolino A, et al. A comprehensive and easy-to-use ECG algorithm to predict the coronary occlusion site in ST-segment elevation myocardial infarction. *American Heart Journal* 2023; **255**: 94-105
51 Safdarian N, Nezhad SYD, Dabanloo NJ. Detection and classification of myocardial infarction with support vector machine classifier using grasshopper optimization algorithm. *Journal of Medical Signals & Sensors* 2021; **11**: 185-93

52 Baxt WG, Shofer FS, Sites FD, Hollander JE. A neural network aid for the early diagnosis of cardiac ischemia in patients presenting to the emergency department with chest pain. *Annals of emergency medicine* 2002; **40**: 575-83
53 Muhlestein JB, Anderson JL, Bethea CF, et al. Feasibility of combining serial smartphone single-lead electrocardiograms for the diagnosis of ST-elevation myocardial infarction. *American heart journal* 2020; **221**: 125-35
54 Liu W, Ji J, Chang S, Wang H, He J, Huang Q. EvoMBN: evolving multi-branch networks on myocardial infarction diagnosis using 12-lead electrocardiograms. *Biosensors* 2021; **12**: 15
55 Cao Y, Liu W, Zhang S, et al. Detection and localization of myocardial infarction based on multi-scale resnet and attention mechanism. *Frontiers in Physiology* 2022; **13**: 783184
56 Zhao Y, Xiong J, Hou Y, et al. Early detection of ST-segment elevated myocardial infarction by artificial intelligence with 12-lead electrocardiogram. *International Journal of Cardiology* 2020; **317**: 223-30
57 Kora P, Kalva SR. Improved Bat algorithm for the detection of myocardial infarction. *SpringerPlus* 2015; **4**: 1-18
58 Barua PD, Aydemir E, Dogan S, et al. Multilevel hybrid accurate handcrafted model for myocardial infarction classification using ECG signals. *International Journal of Machine Learning and Cybernetics* 2023; **14**: 1651-68
59 Firoozabadi R, Gregg RE, Babaeizadeh S. Intelligent use of advanced capabilities of diagnostic ECG algorithms in a monitoring environment. *Journal of Electrocardiology* 2017; **50**: 615-9
60 Liu W, Zhang M, Zhang Y, et al. Real-time multilead convolutional neural network for myocardial infarction detection. *IEEE journal of biomedical and health informatics* 2017; **22**: 1434-44
61 Dhawan A, Wenzel B, George S, Gussak I, Bojovic B, Panescu D. Detection of acute myocardial infarction from serial ECG using multilayer support vector machine. 2704-7
62 Park J, Pedrycz W, Jeon M. Ischemia episode detection in ECG using kernel density estimation, support vector machine and feature selection. *Biomedical engineering online* 2012; **11**: 1-22
63 Sraitih M, Jabrane Y, Hajjam El Hassani A. A robustness evaluation of machine learning algorithms for ECG myocardial infarction detection. *Journal of Clinical Medicine* 2022; **11**: 4935
64 de Capretz PO, Björkelund A, Björk J, et al. Machine learning for early prediction of acute myocardial infarction or death in acute chest pain patients using electrocardiogram and blood tests at presentation. *BMC medical informatics and decision making* 2023; **23**: 25
65 Al-Zaiti S, Martin-Gill C, Zègre-Hemsey J, et al. Machine Learning for the ECG Diagnosis and Risk Stratification of Occlusion Myocardial Infarction at First Medical Contact. *Research Square* 2023
66 Gustafsson S, Gedon D, Lampa E, et al. Development and validation of deep learning ECG-based prediction of myocardial infarction in emergency department patients. *Scientific Reports* 2022; **12**: 19615
67 Choi HY, Kim W, Kang GH, et al. Diagnostic accuracy of the deep learning model for the detection of ST elevation myocardial infarction on electrocardiogram. *Journal of Personalized Medicine* 2022; **12**: 336
68 Liu W-C, Lin C-S, Tsai C-S, et al. A deep learning algorithm for detecting acute myocardial infarction: Deep learning model to detect AMI. *EuroIntervention* 2021; **17**: 765
69 Bosnjak A, Bevilacqua G, Passariello G, Mora F, Sanso B, Carrault G. An approach to intelligent ischaemia monitoring. *Medical and Biological Engineering and Computing* 1995; **33**: 749-56

70 Tadesse GA, Javed H, Weldemariam K, et al. DeepMI: Deep multi-lead ECG fusion for identifying myocardial infarction and its occurrence-time. *Artificial Intelligence in Medicine* 2021; **121**: 102192
71 He Z, Yuan Z, An P, Zhao J, Du B. MFB-LANN: A lightweight and updatable myocardial infarction diagnosis system based on convolutional neural networks and active learning. *Computer Methods and Programs in Biomedicine* 2021; **210**: 106379
72 Bansal A, Kumar S, Bajpai A, et al. Remote health monitoring system for detecting cardiac disorders. *IET Systems Biology* 2015; **9**: 309-14
73 Hammad M, Chelloug SA, Alkanhel R, et al. Automated detection of myocardial infarction and heart conduction disorders based on feature selection and a deep learning model. *Sensors* 2022; **22**: 6503
74 Chen X, Guo W, Zhao L, et al. Acute myocardial infarction detection using deep learning-enabled electrocardiograms. *Frontiers in cardiovascular medicine* 2021; **8**: 654515
75 Fu L, Lu B, Nie B, Peng Z, Liu H, Pi X. Hybrid network with attention mechanism for detection and location of myocardial infarction based on 12-lead electrocardiogram signals. *Sensors* 2020; **20**: 1020
76 Cao Y, Wei T, Zhang B, et al. ML-Net: Multi-channel lightweight network for detecting myocardial infarction. *IEEE Journal of Biomedical and Health Informatics* 2021; **25**: 3721-31
77 Liu B, Liu J, Wang G, et al. A novel electrocardiogram parameterization algorithm and its application in myocardial infarction detection. *Computers in biology and medicine* 2015; **61**: 178-84
78 Jahmunah V, Ng EYK, Tan R-S, Oh SL, Acharya UR. Explainable detection of myocardial infarction using deep learning models with Grad-CAM technique on ECG signals. *Computers in Biology and Medicine* 2022; **146**: 105550
79 Han C, Song Y, Lim H-S, et al. Automated detection of acute myocardial infarction using asynchronous electrocardiogram signals—preview of implementing artificial intelligence with multichannel electrocardiographs obtained from Smartwatches: retrospective study. *Journal of Medical Internet Research* 2021; **23**: e31129
80 Chang K-C, Hsieh P-H, Wu M-Y, et al. Usefulness of multi-labelling artificial intelligence in detecting rhythm disorders and acute ST-elevation myocardial infarction on 12-lead electrocardiogram. *European Heart Journal-Digital Health* 2021; **2**: 299-310
81 Xiao R, Ding C, Hu X, et al. Integrating multimodal information in machine learning for classifying acute myocardial infarction. *Physiological Measurement* 2023; **44**: 044002
82 Hedén B, Öhlin H, Rittner R, Edenbrandt L. Acute myocardial infarction detected in the 12-lead ECG by artificial neural networks. *Circulation* 1997; **96**: 1798-802
83 Wang HM, Zhao W, Jia DY, et al. Myocardial infarction detection based on multi-lead ensemble neural network. 2614-7
84 Jahmunah V, Ng EYK, San TR, Acharya UR. Automated detection of coronary artery disease, myocardial infarction and congestive heart failure using GaborCNN model with ECG signals. *Computers in biology and medicine* 2021; **134**: 104457
85 Garvey JL, Zegre-Hemsey J, Gregg R, Studnek JR. Electrocardiographic diagnosis of ST segment elevation myocardial infarction: an evaluation of three automated interpretation algorithms. *Journal of electrocardiology* 2016; **49**: 728-32
86 Wu L, Huang G, Yu X, et al. Deep learning networks accurately detect ST-segment elevation myocardial infarction and culprit vessel. *Frontiers in cardiovascular medicine* 2022; **9**: 797207
87 Choi YJ, Park MJ, Ko Y, et al. Artificial intelligence versus physicians on interpretation of printed ECG images: Diagnostic performance of ST-elevation myocardial infarction on electrocardiography. *International journal of cardiology* 2022; **363**: 6-10

88 Gibson CM, Mehta S, Ceschim MRS, et al. Evolution of single-lead ECG for STEMI detection using a deep learning approach. *International journal of cardiology* 2022; **346**: 47-52
89 Liu J, Zhang C, Ristaniemi T, Cong F. Detection of myocardial infarction from multi-lead ECG using dual-Q tunable Q-factor wavelet transform. 1496-9
90 Rad MZ, Ghuchani SR, Bahaadinbeigy K, Khalilzadeh MM. Real time recognition of heart attack in a smart phone. *Acta Informatica Medica* 2015; **23**: 151
91 Liu W-C, Lin C, Lin C-S, et al. An artificial intelligence-based alarm strategy facilitates management of acute myocardial infarction. *Journal of Personalized Medicine* 2021; **11**: 1149
92 Tseng Y-L, Lin K-S, Jaw F-S. Comparison of Support-Vector Machine and Sparse Representation Using a Modified Rule-Based Method for Automated Myocardial Ischemia Detection. *Computational and Mathematical Methods in Medicine* 2016; **2016**: 9460375
93 Xiao R, Xu Y, Pelter MM, Mortara DW, Hu X. A deep learning approach to examine ischemic ST changes in ambulatory ECG recordings. *AMIA Summits on Translational Science Proceedings* 2018; **2018**: 256
94 Bigler MR, Seiler C. Detection of myocardial ischemia by intracoronary ECG using convolutional neural networks. *Plos one* 2021; **16**: e0253200
95 De La Fuente-Cortes G, Diaz-Mendez A, Gonzalez-Diaz VR. A fully integrated fuzzy logic algorithm for ischemic heartbeat classification. 873-6
96 Murthy HSN, Meenakshi M. Computer aided diagnosis of myocardial ischemic beats using ICA and WPD. *J Adv Res Dyn Control Syst* 2019; **11**: 214-20
97 Kora P. ECG based myocardial infarction detection using hybrid firefly algorithm. *Computer methods and programs in biomedicine* 2017; **152**: 141-8
98 Hillinger P, Strebel I, Abächerli R, et al. Prospective validation of current quantitative electrocardiographic criteria for ST-elevation myocardial infarction. *International journal of cardiology* 2019; **292**: 1-12
99 Qin L, Qi Q, Aikeliyaer A, Hou WQ, Zuo CX, Ma X. Machine learning algorithm can provide assistance for the diagnosis of non-ST-segment elevation myocardial infarction. *Postgraduate medical journal* 2023; **99**: 442-54
100 Silipo R, Laguna P, Narchesi C, Mark RG. ST-T segment change recognition using artificial neural networks and principal component analysis. 213-6
101 Kapfo A, Dandapat S, Kumar Bora P. Automated detection of myocardial infarction from ECG signal using variational mode decomposition based analysis. *Healthcare Technology Letters* 2020; **7**: 155-60
102 Gururaj V, Shankar SP, Bharadwaj A. Electrocardiogram based cardiovascular disease detection with Ensemble Learning Classifier. 48-53
103 Baxt WG. Use of an artificial neural network for the diagnosis of myocardial infarction. *Annals of internal medicine* 1991; **115**: 843-8
104 Aufderheide TP, Xue Q, Dhala AA, Reddy S, Kuhn EM. The added diagnostic value of automated QT-dispersion measurements and automated ST-segment deviations in the electrocardiographic diagnosis of acute cardiac ischemia. *Journal of electrocardiology* 2000; **33**: 329-39
105 Pan W, An Y, Guan Y, Wang J. MCA-net: A multi-task channel attention network for Myocardial infarction detection and location using 12-lead ECGs. *Computers in Biology and Medicine* 2022; **150**: 106199
106 Sharma LD, Sunkaria RK. Myocardial infarction detection and localization using optimal features based lead specific approach. *Irbm* 2020; **41**: 58-70
107 Kumar M, Pachori RB, Acharya UR. Automated diagnosis of myocardial infarction ECG signals using sample entropy in flexible analytic wavelet transform framework. *Entropy* 2017; **19**: 488

108 Wang Y, Zhu W, Ma G, Chen X, Wang L. Detection and Classification of Anterior Myocardial Infarction Using Combined Linear and Nonlinear Features of ECG Signals. 154-7
109 Chen Y, Chen H, He Z, Yang C, Cao Y. Multi-channel lightweight convolution neural network for anterior myocardial infarction detection. 572-8
110 Xue H, Wang C, Tang M, Deng M. Deep Convolutional and Recurrent Neural Networks for Detection of Myocardial Ischemia Using Cardiodynamics gram. 623-8
111 Ibrahim L, Mesinovic M, Yang K-W, Eid MA. Explainable prediction of acute myocardial infarction using machine learning and shapley values. *Ieee Access* 2020; **8**: 210410-7
112 Dai H, Hwang H-G, Tseng VS. Convolutional neural network based automatic screening tool for cardiovascular diseases using different intervals of ECG signals. *Computer Methods and Programs in Biomedicine* 2021; **203**: 106035
113 Xiong P, Yang L, Zhang J, et al. Detection of inferior myocardial infarction based on multi branch hybrid network. *Biomedical Signal Processing and Control* 2023; **84**: 104725
114 Maglaveras N, Stamkopoulos T, Pappas C, Strintzis M. Use of neural networks in detection of ischemic episodes from ECG leads. 518-24
115 Khoshnoud S, Teshnehlab M, Shoorehdeli MA. Probabilistic neural network oriented classification methodology for Ischemic Beat detection using multi resolution Wavelet analysis. 1-4
116 She J, Zhang J, Liu Z. Recognition of Myocardial Ischemia Electrocardiogram Signal Based on Deep Learning. 1-6
117 Ogrezeanu I, Stoian D, Turcea A, Itu LM. Deep learning based myocardial ischemia detection in ECG signals. 250-3
118 Langley P, Bowers EJ, Wild J, et al. An algorithm to distinguish ischaemic and non-ischaemic ST changes in the Holter ECG. 239-42
119 Bakhshipour A, Pooyan M, Mohammadnejad H, Fallahi A. Myocardial ischemia detection with ECG analysis, using Wavelet Transform and Support Vector Machines. 1-4
120 Mohanta S, Dasgupta K. A Framework for Ischemic Beat Detection using Multi-Layer Feedforward Neural Network and Principal Component Analysis (IBD-MLFFNN-PCA). 77-84
121 Rajput KSC, Kumar M, Kodipalli A, Rohini BR, Rao T, Pushpalatha V. Prediction of Myocardial Infraction Using Machine Learning Algorithms. 326-32
122 Faramand Z, Helman S, Ahmad A, et al. Performance and limitations of automated ECG interpretation statements in patients with suspected acute coronary syndrome. *Journal of electrocardiology* 2021; **69**: 45-50
123 Muminov B, Nasimov R, Mirzahalilov S, Sayfullaeva N, Gadoyboyeva N. Localization and classification of myocardial infarction based on artificial neural network. 245-9
124 Firoz M, Faiz R, Alam NN, Imam MH. Detection of myocardial infarction using hybrid CNN-LSTM model. 254-8
125 Fatimah B, Singh P, Singhal A, Pramanick D, Pachori RB. Efficient detection of myocardial infarction from single lead ECG signal. *Biomedical Signal Processing and Control* 2021; **68**: 102678
126 Mohebbi M, Moghadam HA, Teshnehlab M. An Automated System for On-line Monitoring and Detection of ST Changes in ECG Signal. 1-4
127 Kavak S, Chiu X-D, Yen S-J, Chen MY-C. Application of CNN for detection and localization of STEMI using 12-lead ECG images. *Ieee Access* 2022; **10**: 38923-30
128 Davydov NS, Khramov AG. Myocardial infarction detection using wavelet analysis of ECG signal. 31-7
129 Saha S. Classifiers based on machine learning for detection of myocardial infarction. 1509-14

130 Sahu G, Ray KC. An Efficient Signal Processing Technique for Automated Myocardial Infarction Detection. 93-8
131 Zhang J, Lin F, Xiong P, et al. Automated detection and localization of myocardial infarction with staked sparse autoencoder and treebagger. *IEEE Access* 2019; **7**: 70634-42
132 Wu JF, Bao YL, Chan S-C, Wu HC, Zhang L, Wei X-G. Myocardial infarction detection and classification—A new multi-scale deep feature learning approach. 309-13
133 Liu S, Bin G, Wu S, Zhou Z, Bin G. Detection and Location of Myocardial Infarction from Electrocardiogram Signals Using Median Complexes and Convolutional Neural Networks. 1018-30
134 Han C, Sun J, Bian Y, Que W, Shi L. Automated detection and localization of myocardial infarction with interpretability analysis based on deep learning. *IEEE Transactions on Instrumentation and Measurement* 2023; **72**: 1-12
135 Liu W, Huang Q, Chang S, Wang H, He J. Multiple-feature-branch convolutional neural network for myocardial infarction diagnosis using electrocardiogram. *Biomedical Signal Processing and Control* 2018; **45**: 22-32
136 Swain SS, Patra D, Singh YO. Automated detection of myocardial infarction in ECG using modified Stockwell transform and phase distribution pattern from time-frequency analysis. *Biocybernetics and Biomedical Engineering* 2020; **40**: 1174-89
137 He Z, Yuan S, Zhao J, et al. A novel myocardial infarction localization method using multi-branch DenseNet and spatial matching-based active semi-supervised learning. *Information Sciences* 2022; **606**: 649-68
138 Ma X, Fu X, Sun Y, Wang N, Ning X, Gao Y. Convolutional dendrite net detects myocardial infarction based on ecg signal measured by flexible sensor. 1-4
139 Murthy HN, Meenakshi M. Efficient algorithm for early detection of myocardial ischemia using pca based features. *Indian J Sci Technol* 2016; **9**: 1-13
140 Zhang X, Li R, Hu Q, Zhou B, Wang Z. A new automatic approach to distinguish myocardial infarction based on LSTM. 1-3
141 Rajakumar G, Nagaraju V, Bapu BRT, Malar PSR, Krishnan RS, Narayanan KL. Myocardial Infarction Analysis Using Deep Learning Neural Network Based on Image Processing Approach. *Distributed Computing and Optimization Techniques: Select Proceedings of ICDCOT 2021*: Springer, 2022; 635-44
142 Lin Z, Gao Y, Chen Y, Ge Q, Mahara G, Zhang J. Automated detection of myocardial infarction using robust features extracted from 12-lead ECG. *Signal, Image and Video Processing* 2020; **14**: 857-65
143 Mohebbi M, Moghadam HA. An algorithm for automated detection of ischemic ECG beats using support vector machines. 1-4
144 Zhao T, Deng M, Lin P, Wang J, Cao J. Classification of myocardial infarction based on ECG signals and multi-network stacking model. 7266-70
145 Bhaskar NA. Performance analysis of support vector machine and neural networks in detection of myocardial infarction. *Procedia Computer Science* 2015; **46**: 20-30
146 Yadav SS, More SB, Jadhav SM, Sutar SR. Convolutional neural networks based diagnosis of myocardial infarction in electrocardiograms. 581-6
147 Wang Z, Qian L, Han C, Shi L. Application of multi-feature fusion and random forests to the automated detection of myocardial infarction. *Cognitive Systems Research* 2020; **59**: 15-26
148 Dey M, Omar N, Ullah MA. Temporal feature-based classification into myocardial infarction and other cvds merging cnn and bi-lstm from ecg signal. *IEEE Sensors Journal* 2021; **21**: 21688-95

149 Sinha N, Das A. Identification and localization of myocardial infarction based on analysis of ECG signal in cross spectral domain using boosted SVM classifier. *IEEE Transactions on Instrumentation and Measurement* 2021; **70**: 1-9
150 Reasat T, Shahnaz C. Detection of inferior myocardial infarction using shallow convolutional neural networks. 718-21
151 Omar N, Dey M, Ullah MA. Detection of myocardial infarction from ECG signal through combining CNN and Bi-LSTM. 395-8
152 Ma X, Fu X, Sun Y, Wang N, Gao Y. Application of convolutional dendrite net for detection of myocardial infarction using ecg signals. *IEEE Sensors Journal* 2022; **23**: 460-9
153 Finlay DD, Guldenring D, Bond RR, Daly MJ. An algorithm for the detection of ST segment elevation relating to induced ischemia in body surface potential maps. 361-4
154 Li T, Dong F, Hirota K. Fuzzy association rule mining based myocardial ischemia diagnosis on ECG signal. *Journal of Advanced Computational Intelligence and Intelligent Informatics* 2015; **19**: 217-24
155 Martin H, Izquierdo W, Cabrerizo M, Cabrera A, Adjouadi M. Near real-time single-beat myocardial infarction detection from single-lead electrocardiogram using Long Short-Term Memory Neural Network. *Biomedical Signal Processing and Control* 2021; **68**: 102683
156 Xu W, Wang L, Wang B, Cheng W. Intelligent Recognition Algorithm of Multiple Myocardial Infarction Based on Morphological Feature Extraction. *Processes* 2022; **10**: 2348
157 Diker A, Cömert Z, Avci E, Velappan S. Intelligent system based on Genetic Algorithm and support vector machine for detection of myocardial infarction from ECG signals. 1-4
158 Darmawahyuni A, Nurmaini S, Sukemi, et al. Deep learning with a recurrent network structure in the sequence modeling of imbalanced data for ECG-rhythm classifier. *Algorithms* 2019; **12**: 118
159 Darmawahyuni A, Nurmaini S. Deep learning with long short-term memory for enhancement myocardial infarction classification. 19-23
160 Zhang G, Si Y, Wang D, Yang W, Sun Y. Automated detection of myocardial infarction using a gramian angular field and principal component analysis network. *IEEE Access* 2019; **7**: 171570-83
161 Lui HW, Chow KL. Multiclass classification of myocardial infarction with convolutional and recurrent neural networks for portable ECG devices. *Informatics in Medicine Unlocked* 2018; **13**: 26-33
162 Zhang Y, Li J. Application of heartbeat-attention mechanism for detection of myocardial infarction using 12-lead ECG records. *Applied Sciences* 2019; **9**: 3328
163 Mirza AH, Nurmaini S, Partan RU. Automatic classification of 15 leads ECG signal of myocardial infarction using one dimension convolutional neural network. *Applied Sciences* 2022; **12**: 5603
164 Bhaskarpandit S, Gade A, Dash S, Dash DK, Tripathy RK, Pachori RB. Detection of myocardial infarction from 12-lead ECG trace images using eigendomain deep representation learning. *IEEE Transactions on Instrumentation and Measurement* 2023; **72**: 1-12
165 Choudhary PS, Dandapat S. Multibranch 1D CNN for detection and localization of myocardial infarction from 12 lead electrocardiogram signal. 1-5
166 Han C, Pan S, Que W, Wang Z, Zhai Y, Shi L. Automated localization and severity period prediction of myocardial infarction with clinical interpretability based on deep learning and knowledge graph. *Expert Systems with Applications* 2022; **209**: 118398
167 Jikui L, Ruxin W, Bo W, Zengding L, Fen M, Ye LI. Myocardial infarction detection and localization with electrocardiogram based on convolutional neural network. *Chinese Journal of Electronics* 2021; **30**: 833-42

168 Elmannai H, Saleh H, Algarni AD, et al. Diagnosis myocardial infarction based on stacking ensemble of convolutional neural network. *Electronics* 2022; **11**: 3976
169 Sopic D, Aminifar A, Aminifar A, Atienza D. Real-time classification technique for early detection and prevention of myocardial infarction on wearable devices. 1-4
170 Dohare AK, Kumar V, Kumar R. Detection of myocardial infarction in 12 lead ECG using support vector machine. *Applied Soft Computing* 2018; **64**: 138-47
171 Nasimov R, Muminov B, Mirzahalilov S, Nasimova N. A new approach to classifying myocardial infarction and cardiomyopathy using deep learning. 1-5
172 Feng K, Pi X, Liu H, Sun K. Myocardial infarction classification based on convolutional neural network and recurrent neural network. *Applied Sciences* 2019; **9**: 1879
173 Baloglu UB, Talo M, Yildirim O, San Tan R, Acharya UR. Classification of myocardial infarction with multi-lead ECG signals and deep CNN. *Pattern recognition letters* 2019; **122**: 23-30
174 Tripathy RK, Bhattacharyya A, Pachori RB. A novel approach for detection of myocardial infarction from ECG signals of multiple electrodes. *IEEE Sensors Journal* 2019; **19**: 4509-17
175 Sridhar C, Lih OS, Jahmunah V, et al. Accurate detection of myocardial infarction using non linear features with ECG signals. *Journal of Ambient Intelligence and Humanized Computing* 2021; **12**: 3227-44
176 Peláez JI, Doña JM, Fornari JF, Serra G. Ischemia classification via ECG using MLP neural networks. *International Journal of Computational Intelligence Systems* 2014; **7**: 344-52
177 Li W, Tang YM, Yu KM, To S. SLC-GAN: An automated myocardial infarction detection model based on generative adversarial networks and convolutional neural networks with single-lead electrocardiogram synthesis. *Information Sciences* 2022; **589**: 738-50
178 Sharma LD, Sunkaria RK. Inferior myocardial infarction detection using stationary wavelet transform and machine learning approach. *Signal, Image and Video Processing* 2018; **12**: 199-206
179 Sadhukhan D, Pal S, Mitra M. Automated identification of myocardial infarction using harmonic phase distribution pattern of ECG data. *IEEE Transactions on Instrumentation and Measurement* 2018; **67**: 2303-13
180 Andreao RV, Dorizzi B, Boudy J, Mota JCM. ST-segment analysis using hidden Markov Model beat segmentation: application to ischemia detection. 381-4
181 Rai HM, Chatterjee K. Hybrid CNN-LSTM deep learning model and ensemble technique for automatic detection of myocardial infarction using big ECG data. *Applied Intelligence* 2022; **52**: 5366-84
182 Moghadam SR, Asl BM. Automatic diagnosis and localization of myocardial infarction using morphological features of ECG signal. *Biomedical Signal Processing and Control* 2023; **83**: 104671

Supplementary Information 3. Detailed Description of AI Technology used in ECG Interpretation.

The specific AI techniques investigated in the primary studies can be broadly categorized into algorithm-based (n =53), traditional machine learning (n=27), signal processing (n=2), neural network (n=95), and hybrid network (n=12). These categories reflect the historical evolution of artificial intelligence (AI) in electrocardiogram (ECG) analysis, particularly for myocardial infarction (MI) or ischemia detection, which has progressed from expert systems and rule-based logic to classical machine learning models, and more recently, to sophisticated deep learning networks.

Rule-based algorithms, also referred to as expert systems, are grounded in predefined criteria or thresholds to interpret ECG changes. For example, a system might flag ST-segment elevation exceeding a particular amplitude or duration as indicative of ischemia. These systems are highly interpretable and customizable for specific clinical priorities (e.g., greater sensitivity to ST depression versus STEMI). However, they lack adaptability to atypical ECG presentations or noisy data and cannot improve over time since they do not learn from input data. Their logic is transparent but rigid, limiting their use in real-world, high-variability clinical environments.

Traditional machine learning techniques, such as support vector machines (SVMs) and decision trees, represent an advancement over rule-based systems by learning patterns from labeled data. These models rely on manually engineered ECG features (e.g., ST slopes, QRS durations) to predict ischemia or classify signals as normal versus abnormal. They offer better generalizability than rule-based approaches and are relatively simple to implement and validate. However, they are constrained by their dependence on expert-driven feature extraction, which introduces human bias and may not capture the complex, non-linear characteristics of ECG signals—particularly in noisy, real-time perioperative contexts.

Artificial Neural Networks (ANNs) move beyond manual feature engineering by learning non-linear relationships and abstract patterns directly from the data. While flexible and capable of handling both structured and unstructured inputs, ANNs are not architecturally optimized for spatially or temporally structured data like ECGs. They require large volumes of data to perform well and can be less effective when applied to spatially localized features critical for detecting subtle ischemic patterns.

Convolutional Neural Networks (CNNs) represent a major leap forward for ECG analysis. Specifically designed for grid-like data, such as images or waveform signals, CNNs employ convolutional layers that apply filters to detect spatial patterns within the ECG, such as ST-segment elevations or depressions. CNNs eliminate the need for manual feature extraction, thereby reducing human bias and improving efficiency. Their strengths include fast training, automatic feature learning, and high effectiveness in ECG classification tasks. However, their limited ability to model long-range temporal dependencies and their performance degradation in highly noisy signals are noted limitations.

Residual Networks (ResNet) extend the CNN architecture by incorporating hundreds of layers connected via "residual blocks" or skip connections. These skip connections allow the model to bypass certain layers, enabling the training of deeper networks without the usual degradation in performance. ResNets are particularly adept at capturing subtle and complex ECG patterns that may be distributed across longer sequences, thereby increasing their generalizability to real-

world ECG variability. Nevertheless, ResNet applications in perioperative or real-time settings remain underexplored, and their high computational demand may limit clinical deployment.

Densely Connected Convolutional Networks (DenseNet) further improve upon CNN architectures by promoting direct connections between all layers, thereby enhancing gradient flow and mitigating the vanishing gradient problem. This design ensures efficient learning even in very deep networks, making DenseNets suitable for processing complex ECG data with greater stability.

Hybrid networks, such as the CNN-LSTM (Long Short-Term Memory) architecture, represent the most recent and promising advancement in AI for MI detection. This architecture combines the spatial pattern recognition capabilities of CNNs with the temporal sequence learning strength of LSTMs. LSTM units are particularly effective in modeling time-dependent changes and overcoming the vanishing gradient problem. Together, the hybrid network allows for both nuanced feature extraction and dynamic pattern recognition across time—key to enhancing sensitivity and specificity in ischemia detection in continuously monitored ECGs.

These diverse AI architectures not only reflect a technological progression but also address different clinical and operational challenges in ECG-based ischemia detection. While rule-based systems offer interpretability and speed, neural and hybrid networks bring adaptability, precision, and scalability to complex ECG interpretation, paving the way for more intelligent and robust decision-support tools in perioperative and acute care settings.
